# Development of a Tunable Photoreactor for High-Throughput Experimentation

**DOI:** 10.1021/jacs.6c07607

**Published:** 2026-09-09

**Authors:** Reem Nsouli, Hai N. Tran, Robbie S. Sidhu, Hannah G. Ford, Andrew V. Tran, Harpreet Kaur, Gaurav Galiyan, Laura K. G. Ackerman-Biegasiewicz

**Affiliations:** † Department of Chemistry, 1371 Emory University, Atlanta, Georgia 30322, United States; ‡ Singh Instrument, Livermore, California 94551, United States; § School of Molecular Sciences, 7864 Arizona State University, Tempe, Arizona 85281, United States

## Abstract

Photochemistry has revolutionized modern chemical synthesis, offering new modes of bond activation and construction under mild conditions. Yet, the systematic study of light as a tunable reaction variable remains underdeveloped, as commercial high-throughput experimentation (HTE) platforms lack the flexibility to independently tune light parameters. These constraints hinder the discovery, optimization, and understanding of light-dependent reactivity, which is critical for identifying novel photocatalysts and transformations. To meet this need in photochemistry, we disclose the development of a tunable 24-well photoreactor that enables independent control of wavelength, light intensity, and irradiation time per well to efficiently screen light-driven reactions. Herein we showcase benchmarking studies with Fe, Ce, and Cu visible light-induced homolysis (VLIH) chemistry, demonstrating that the photoreactor can facilitate excellent reproducibility across wells, attain comparable yields to commercial Kessil lamps or CFLs, and enable parallel time course studies at different wavelengths and intensities. To showcase the significance of exploring light parameters in HTE, the tunable platform was leveraged to identify multivariable correlations when selecting ideal catalyst conditions. The decarboxylative coupling between gemfibrozil and benzyl acrylate to access a quaternary center was examined with FeCl_3_ and various ligands under UV (λ_max_ = 395 nm), blue (λ_max_ = 454 nm), and broad-spectrum white light. This study revealed wavelength- and catalyst-dependent reactivity patterns, whereby the use of different ligands required varied wavelengths for optimal yields, underscoring the importance of using multidimensional light screening for reaction discovery.

## Graphical abstract

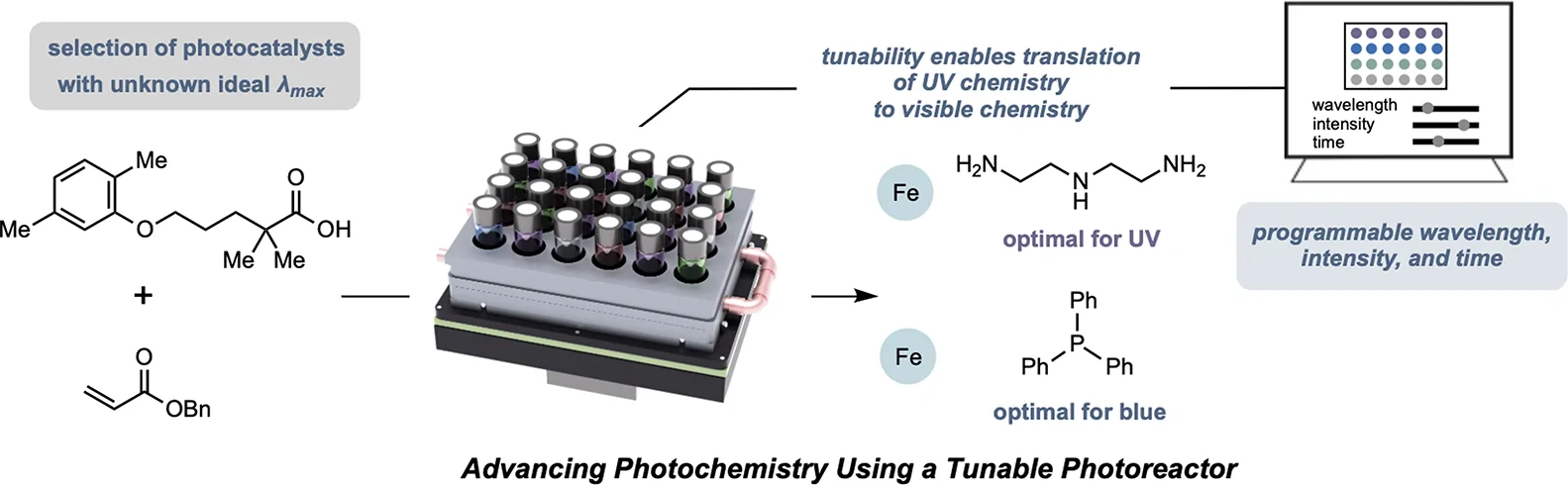

Light-driven transformations have become an indispensable and versatile tool in modern synthetic chemistry.^1–3^ Photochemical methods have broad applications across diverse fields, including polymer science, molecular photoswitches, and catalysis (Figure 1a).^4–6^ The ability of photons to access energetically distinct pathways has enabled new modes of reactivity. Tuning light variables, including wavelength and intensity, has been leveraged to tune reactivity in photocatalysis.^7–11^ For example, the reactivity of organic dyes can be modulated by simply changing the wavelength of irradiation.^12–15^ This concept has also been showcased for visible light-induced homolysis (VLIH) photocatalysts,^16^ which rely on the formation of photocatalytic species *in situ* which are not well-characterized.^17,18^ These examples reinforce the importance of identifying the optimal irradiation parameters for productive and selective transformations.

**Figure 1:**
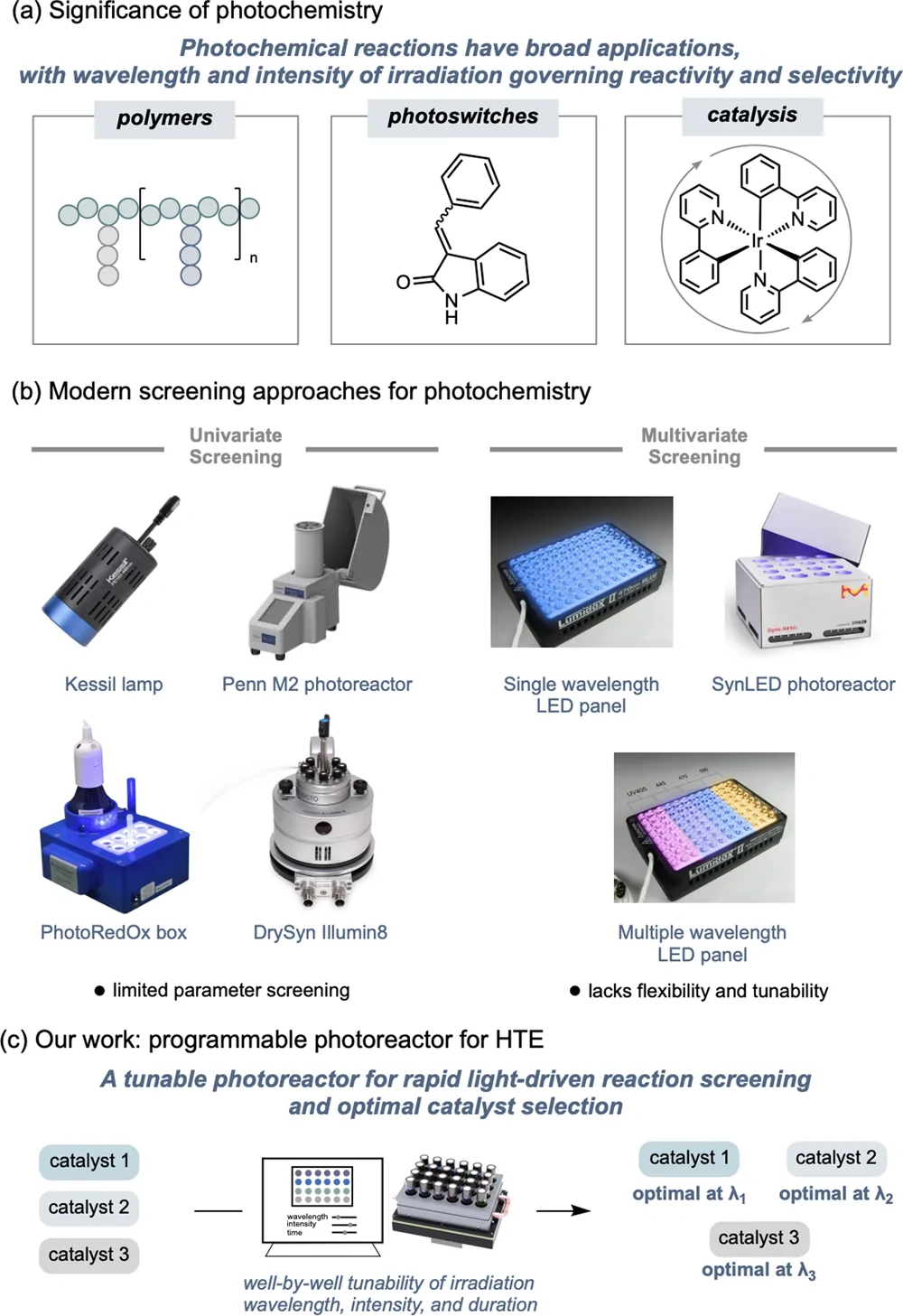
Advancing photochemistry through photoreactor design. (a) The significance of photochemistry across diverse fields. (b) Commercial platforms for screening light-driven transformations and their limitations. (c) The design of a tunable 24-well photoreactor to accelerate and diversify photochemical screening. Images of instruments have been used with permission from respective manufacturers.

Traditionally, the discovery and optimization of photoredox reactions have relied on univariate screening with fixed wavelength light sources (Figure 1b).^19,20^ However, this approach can be time-consuming and limited, especially when exploring new catalyst classes or conditions. Thus, the integration of photoredox catalysis with high-throughput experimentation (HTE) has emerged as a powerful strategy.^21,22^ Several commercial platforms^23–25^ now enable parallel photochemical screening of multiple reactions under a single wavelength. For higher throughput, Analytical Sales and Services has developed a suite of photochemical HTE platforms that extend this capability to 96-well formats (Figure 1b).^25,26^ These configurations allow for multivariate screening across wavelength domains but still operate under fixed, nonprogrammable wavelengths and intensities. Consequently, this restricts systematic exploration of how light parameters influence reactivity, making parallel evaluation under varying irradiation conditions challenging and prone to inconsistency. In contrast, advances in optogenetics and bioengineering have advanced the development of highly tunable platforms that enable versatility in light-associated parameters.^27–29^ However, these systems operate at light intensities several orders of magnitude lower than what is required for efficient photocatalysis (typically in μW/cm^2^ compared to tens to hundreds of mW/cm^2^). While several platforms overcome these limitations (see SI, section II),^25,30^ these solutions are not commercial and/or do not allow for maximized tunability of wavelength parameters (Figure 1b).

To address this challenge, we developed a modular 24-well photoreactor (24-well plate) that offers independent control over the irradiation wavelength, intensity, and time on a per-well basis (Figure 2a) and seamlessly integrates with existing automation platforms and analytical workflows (Figures S2 and S3). A custom LED panel beneath the plate is equipped with high-power RGBW LEDs (red (λ_max_ = 623 nm), green (λ_max_ = 525 nm), blue (λ_max_ = 454 nm), and broad-spectrum white) and an additional UV LED (λ_max_ = 395 nm) at each well position. The panel is operated by an integrated software that enables independent programming of light parameters per-LED (Figure S4), providing a unique platform to systematically examine the relationship between wavelength and other reaction parameters, ideal for design of experiments (DoE) optimization campaigns.^31,32^

**Figure 2:**
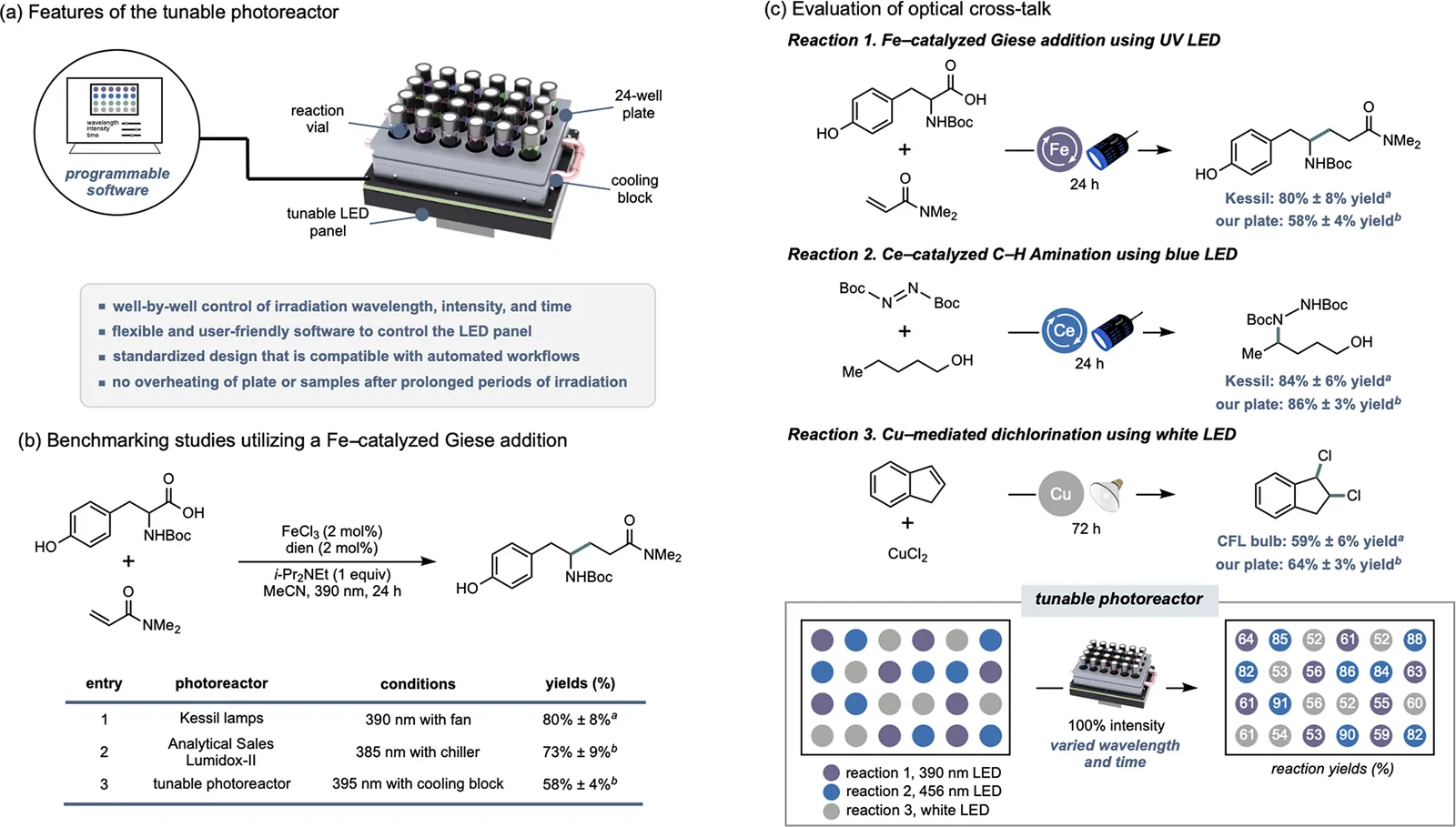
Development of a tunable photoreactor. (a) Features of the developed 24-well tunable photoreactor. (b) Benchmarking studies to compare reaction performance to the commercial Kessil PR160 lamp and Analytical Sales platform for a Fe-catalyzed Giese addition reaction. (c) Evaluation of optical cross-talk via randomized screening of three VLIH-mediated transformations that require different wavelengths and time of irradiation using Fe (UV LED, 24 h), Ce (blue LED, 24 h), and Cu (white LED, 72 h). ^a^ *n* = 10. ^b^ *n* = 24.

To begin evaluating the robustness of the tunable photoreactor, we examined our previously reported Fe-catalyzed decarboxylative Giese addition methodology (Figure 2b).^33^ The coupling of Boc-Tyr–OH and *N,N*-dimethylacrylamide was tested, providing access to a γ-amino amide motif which can serve as a key scaffold for diversification or in medicinally relevant compounds.^34^ At 395 nm and 100% intensity of irradiation, the desired coupled product was obtained in yields averaging 58% ± 4% (n = 24) (Figure S13). Notably, the observed yields were comparable to reactions using the Analytical Sales Lumidox II LED array (385 nm) for the temperature-controlled photoreactor (73% ± 9% yield, n = 24). It is likely that the difference between 390 nm Kessil lamp yields (80% ± 8% yield, n = 10) and the analytical sales plate and the tunable photoreactor result from differences in temperature control with a fan versus a chiller. Furthermore, the tunable photoreactor was utilized to evaluate other medicinally relevant transformations that require different wavelengths of irradiation, further demonstrating its synthetic utility and providing the opportunity for wavelength-dependent selectivity. A Ce-catalyzed 1,5-HAT promoting amination from free alcohols under blue light irradiation^35^ and a Cu-mediated dichlorination of alkenes under white light^36^ were selected as benchmark reactions (Figure 2b). These reactions delivered yields comparable to the 456 nm Kessil lamps (86% ± 3% yield, n = 24 and 84% ± 6% yield, n = 10, respectively) and CFLs (64% ± 3% yield, n = 24 and 59% ± 6% yield, n = 10, respectively) and exhibited excellent reproducibility across the full plate (Figures S14 and S16). Notably, consistent reaction performances were observed across the plate even when the set of experiments was conducted by a distinct researcher (84% ± 5% yield, n = 24), further confirming the robustness and reliability of the platform (Figure S9).

It is worth noting that for photocatalytic reactions, light irradiation can also induce photothermal heating which can impact reaction yield or kinetics. Thus, we conducted a series of experiments to assess temperature uniformity across the custom-designed photoreactor. To evaluate temperature fluctuations, MeCN was irradiated at 454 nm and the solution temperature was monitored across the plate at various time points (Table S1). It was observed that the temperature of reactions stabilized after 30 min of irradiation and the average temperature after 6 h was 30.1 °C ± 0.3 °C. Additionally, minimal discrepancy in temperature was noted when operating the plate using different wavelengths (Table S6) or different intensities of light (Table S7) in a single experiment.

Given the ability to customize wavelength per experiment, running diverse reactions within a single experiment is an attractive application of the tunable platform. Therefore, we subsequently examined interwell optical isolation by randomly distributing the three VLIH-mediated transformations using Fe (395 nm LED, 24 h),^33^ Ce (454 nm LED, 24 h),^35^ and Cu (white LED, 72 h)^36^ salts simultaneously across the plate (Figure 2c). The start and stop times of these reactions could be programmed independently with the implemented software. All the reactions conducted led to the desired product in yields identical to those obtained when each reaction was individually examined across the plate. These results demonstrate a novel experimental design for light-driven reactivity by enabling simultaneous evaluation of distinct reactions with enhanced flexibility and negligible interference between wells.

To gain insights into photochemical transformations, reaction progress and on/off studies are key experiments that provide information about reaction induction periods, catalyst turnover, mechanism, and byproduct generation. However, photochemical time-course studies remain challenging, as many photocatalysts are sensitive to oxygen and moisture, leading to reproducibility issues upon taking sequential aliquots from a single sample. Alternatively, time-course acquisition across multiple identical reactions often results in inconsistency due to variability in irradiation intensity and temperature control across conventional light sources.^37–41^ To address this limitation and showcase the utility of programmable irradiation time, we conducted a time-course study using the previously described model reactions. By terminating reactions at different time points, we obtained reaction progress profiles for three distinct reactions within a single experimental setup (Figure 3a). Importantly, this modular layout can be readily reconfigured to accommodate specific experimental requirements. Collectively, this approach allows for efficient data collection for several reactions simultaneously without the need for sequential sampling and can be easily translated to kinetic experiments. Notably, the Ce-catalyzed and the Cu-mediated reactions exhibited no induction period, with rapid product formation within the first 2 h. In contrast, the Fe-catalyzed reaction displayed a slower initial rate followed by gradual product generation within 16 h.

**Figure 3:**
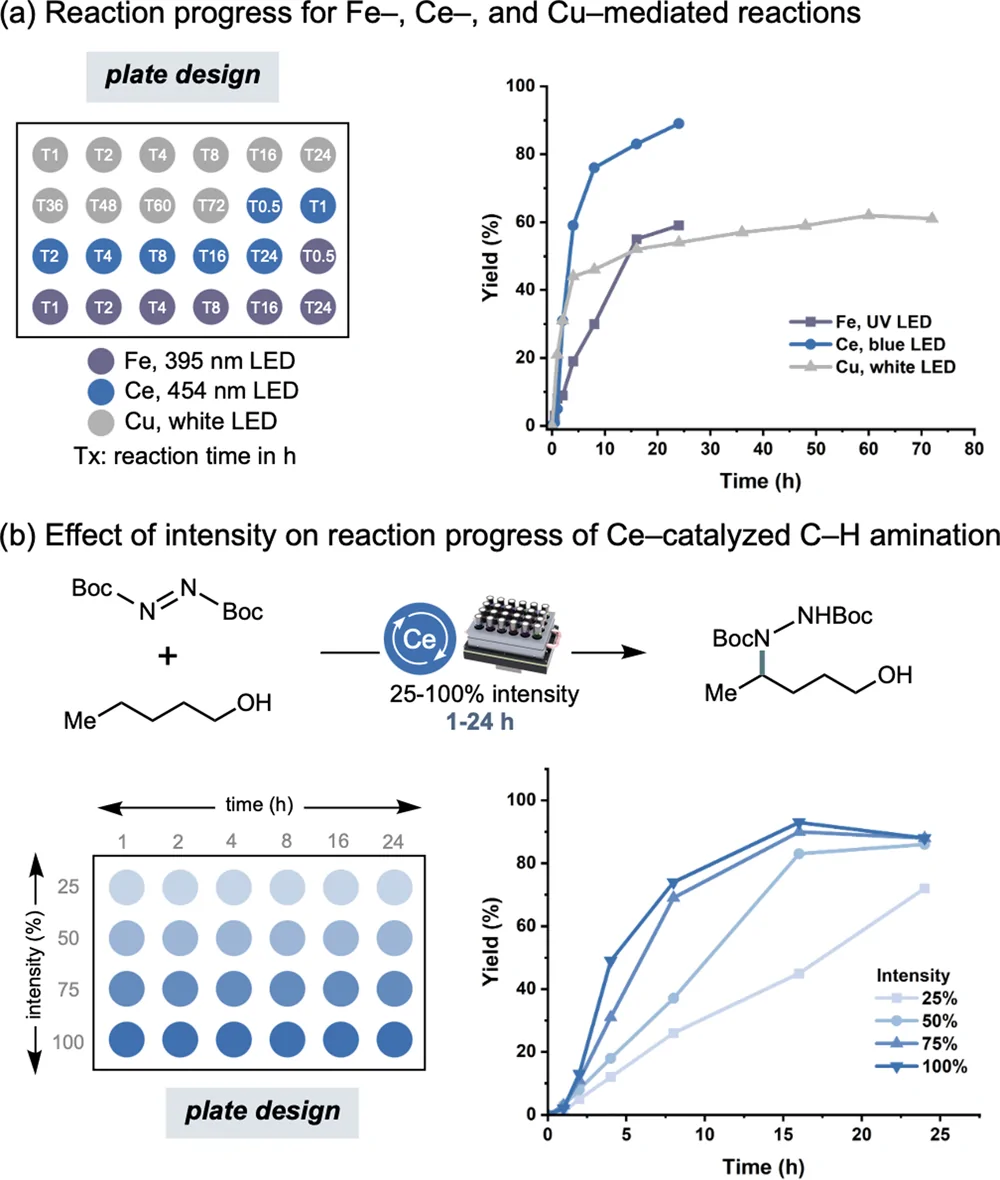
Evaluating reaction profiles using the tunable photoreactor. (a) Reaction progress of 3 distinct VLIH-mediated reactions in a single experiment. (b) Effect of wavelength intensity on reaction progress of a Ce-catalyzed C–H amination.

Additionally, for the Ce-catalyzed reaction, the effect of light intensity on reaction rate was evaluated by simultaneously varying intensity and reaction time across rows and columns, respectively (Figure 3b). The modulation of intensity while acquiring time-course data is not readily achievable with conventional setups, where intensity control is limited and experiments are typically conducted sequentially across multiple independent experiments. Therefore, this integrated approach enables rigorous evaluation of reaction progress under systematically varied photon flux, which provides a data-rich foundation for mechanistic insights and optimization. As expected, increased light intensity led to accelerated reaction rates and notably no product decomposition was observed even at high intensity.^39^ Together, these results underscore the utility and reliability of this platform in efficiently screening photochemical reactions, where incorporation of wavelength as a tunable variable provides the opportunity to accelerate reaction exploration.

In medicinal chemistry workflows, the ability to rapidly evaluate reaction conditions and catalysts to identify successful reaction outcomes is valuable. However, VLIH-catalyzed reactions can be highly sensitive to subtle changes in reaction conditions, as the active species are generated In conventional chemistry workflows, the ability to rapidly evaluate reaction conditions and catalysts to identify successful reaction outcomes is valuable. However, with the development of many emerging photocatalytic reactions this ability to tune reaction parameters becomes even more essential. VLIH-catalyzed reactions, for example, can be highly sensitive to subtle changes in reaction conditions, as the active species are generated in situ, vary across substrates, and exhibit photophysical properties that remain largely underexplored (Figure 4a). Therefore, evaluation of wavelength of irradiation along with other variables is important for novel reaction discovery. To probe these effects systematically, we applied the tunable platform to the decarboxylative Giese addition of gemfibrozil and benzyl acrylate.^33^ Using FeCl_3_, eight Fe-ligand combinations were examined under 395 nm, 454 nm, and white LEDs (Figure 4b). This configuration enabled direct comparison of product formation under multiple wavelengths within the same plate. This experiment revealed pronounced ligand- and wavelength- dependent trends. In the absence of ligand, irradiation at 395 nm, 454 nm, and white light generated 45%, 40%, and 20% yield, respectively (Table S8). Using the previously reported optimal ligand **L1**, the reaction proceeded efficiently under 395 nm irradiation, gave low yields under 454 nm light, and showed no reactivity under white light. In contrast to the nitrogen-based ligands tested, phosphine ligands (**L2**, **L5**, and **L8**) showcased modest improvements in yield under 454 nm compared to 395 nm irradiation and moderate yield under white light. This experiment showcases the ability to translate near UV chemistry to the visible, results that would have likely been missed with univariate screening. The ability to modulate conditions on a per-well basis enabled efficient optimization of irradiation parameters in contrast to current state-of-the-art high-throughput photoreactors^26^ and provided insights for reaction development without the need for redundant or sequential screening.

**Figure 4:**
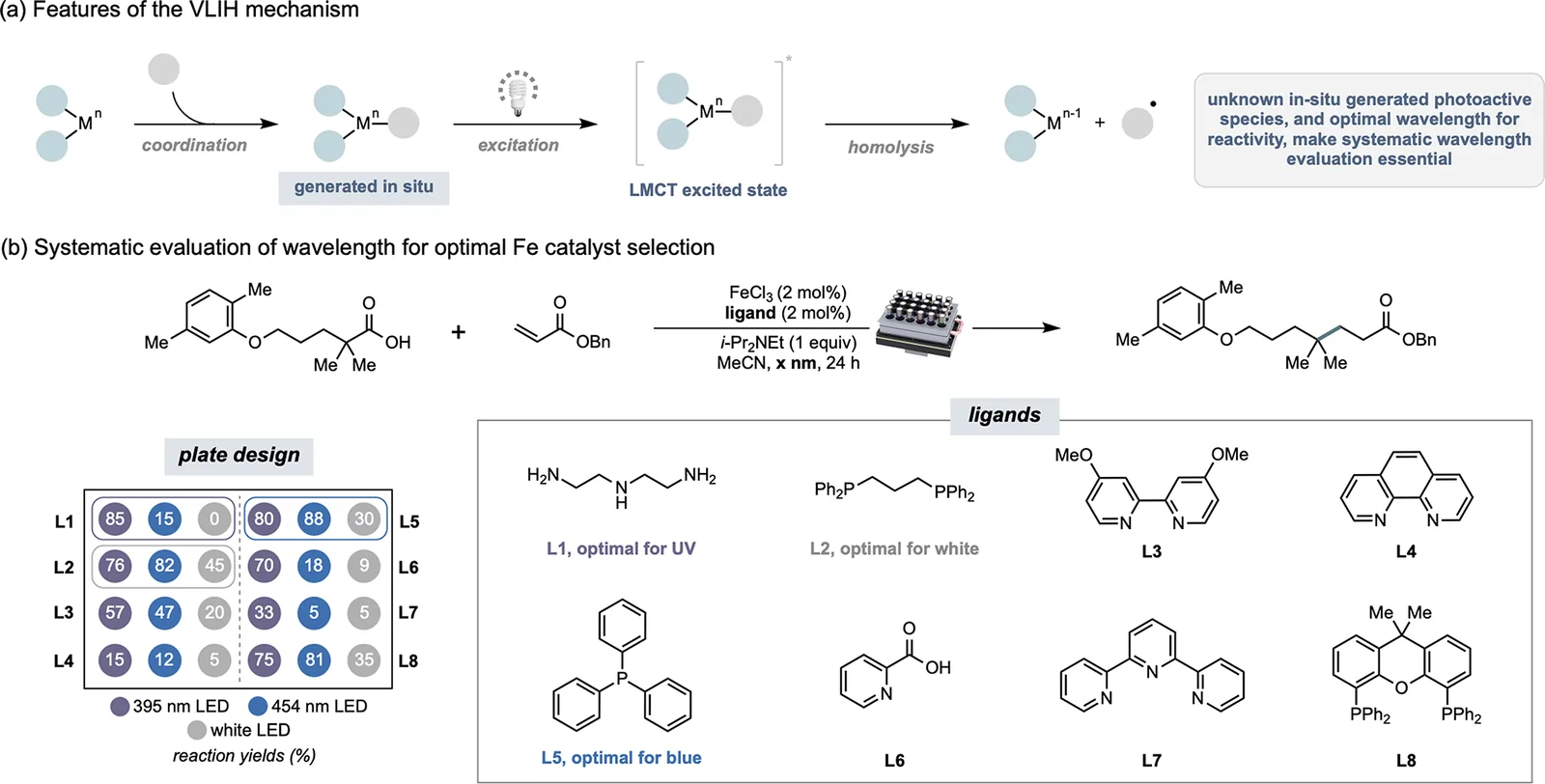
Application of the tunable photoreactor for catalyst and wavelength selection. (a) VLIH mechanism and the significance of systematic wavelength evaluation. (b) Evaluation of Fe-ligand combinations in the decarboxylative Giese addition of gemfibrozil to benzyl acrylate.

In this report we communicate the development of a tunable 24-well photoreactor that enables independent control of wavelength, light intensity, and irradiation time per well to efficiently examine distinct light-driven reactions within a single experiment. Benchmarking studies using VLIH catalysts under 395 nm, 454 nm, or white light demonstrated excellent reproducibility across wells and provided reliable comparison of reaction outcomes to Kessil lamps and CFLs. These results can be extrapolated to explore green and red light-mediated reactions. Moreover, reaction progress can be monitored over time within a single platform to facilitate kinetic studies without significant temperature variation. Notably, the plate design allowed for systematic evaluation of Fe VLIH catalysts, uncovering how different Fe-ligand combinations respond to varying irradiation wavelengths. We envision that this platform will refine aproaches for light-driven reaction optimization and enable access to complex and diverse molecules within a single plate format. Moreover, this work establishes a foundation for the development of higher density 48- and 96-well formats, which have the potential to address the long-standing challenge of achieving data-rich high-throughput experimentation.

## Supplementary Material



## Notes

The Supporting Information includes details about the tunable photoreactor design, software instructions, and experimental procedures. Sourcing and grades of chemicals and materials and characterization of compounds are included. (PDF)
