# Supplementary material for: Development of a Tunable Photoreactor for High-Throughput Experimentation
Source: J Am Chem Soc. 2026 Sep 9;148(37):39440–5. doi: 10.1021/jacs.6c07607 (PMC13614194; doi:10.1021/jacs.6c07607)
Supplement: Supplementary file 1 [file ja6c07607_si_001.pdf]

# Development of a Tunable Photoreactor for High-Throughput Experimentation

Reem Nsouli[‡,1], Hai N. Tran[‡,1], Robbie S. Sidhu[2], Hannah G. Ford[1], Andrew V. Tran[1], Harpreet Kaur[1], Gaurav Galiyan[3], Laura K. G. Ackerman-Biegasiewicz[*,1]

[1]Department of Chemistry, Emory University, Atlanta, GA, 30322, United States
[2]Singh Instrument LLC, Livermore, CA, 94551, United States
[3]School of Molecular Sciences, Arizona State University, Tempe, AZ, 85281, United States

# Supporting Information

# Table of Contents

**I. GENERAL INFORMATION ........ 3**

**II. SURVEY OF CURRENT COMMERCIAL AND CUSTOMIZED PHOTOREACTORS ........ 4**

**III. DESIGN OF THE TUNABLE PHOTOREACTOR ........ 5**

A. Design Strategy ........ 5
B. Design Advances ........ 5
C. Plate Design ........ 6
D. Plate Use ........ 7
E. Software Integration ........ 8
F. Example Procedure for Building a Method Using This Software ........ 8

**IV. EXPERIMENTAL PROCEDURES USING THE TUNABLE PHOTOREACTOR ........ 9**

A. Standardization and Reproducibility ........ 9
B. Benchmarking and Comparison to Conventional Light Sources ........ 13
C. Reaction Progress ........ 23
D. Procedure for Catalyst Selection for Fe–Catalyzed Derivatization of Gemfibrozil 26

**V. UV–VIS SPECTROSCOPY ........ 28**

A. $FeCl_3$ + dien (L1) ........ 28
B. $FeCl_3$ + dppp (L2) ........ 29
C. $FeCl_3$ + $PPh_3$ (L5) ........ 29

**VI. COMPOUND CHARACTERIZATION ........ 31**

**VII. NMR SPECTRA ........ 34**

**VIII. REFERENCES ........ 42**

## I. General Information

**Materials.** Unless otherwise stated, all reagents and substrates are commercial, purchased as reagent grade, and used without further purification. Reagents were sourced from Sigma-Aldrich, Oakwood Chemical, Combi-Blocks, and Ambeed. Unless otherwise noted, anhydrous solvents sourced from Sigma-Aldrich were utilized. $CDCl_3$ was purchased from Sigma-Aldrich and dried over 4 Å molecular sieves. All reactions were conducted under an inert atmosphere by standard Schlenk techniques as noted in the procedures. The 1-dram vial (glass vials, 470151-622) and PTFE-coated stir bar (spinbar micro, 3 x 10 mm, 58948-375) were obtained from VWR® and the septum cap and septum (CG-4910-01 and CV-4080-0013 respectively) were obtained from Chemglass and used as purchased.

**Methods.** Reactions were monitored using thin layer chromatography on glass-backed Uniplate plates with GHLF 250 $\mu$m silica gel. Compounds were visualized using Ultraviolet (UV) light (254 nm) fluorescence quenching, potassium permanganate ($KMnO_4$), or phosphomolybdic acid (PMA) stain. Rotary evaporation was accomplished using either a Heidolph Hei-VAP Core or Hei-VAP Ultimate rotary evaporator at 20-30 °C and <50 torr. Silica gel column chromatography used SiliaFlash P60 silica gel with 230-400 mesh (40-63 $\mu$m particle size).

**Instrumentation.** NMR spectra were recorded on a Bruker spectrometer equipped with a 5 mm iProbe ($^1$H at 400 MHz and 500 MHz and $^{13}$C at 101 MHz). Chemical shifts are reported in parts per million (ppm) downfield from tetramethylsilane and are referenced to a residual NMR solvent signal ($CDCl_3$ = 7.26 ppm for $^1$H and 77.16 ppm for $^{13}$C). NMR data are represented as follows: chemical shift (multiplicity (s = singlet, d = doublet, t = triplet, q = quartet, p = pentet, dd = doublet of doublet, dt = doublet of triplet, m = multiplet), coupling constant in Hertz (Hz), integration). Ultraviolet-visible (UV–Vis) absorbance spectra were collected on a Shimadzu UV-3600 UV–Vis–NIR spectrophotometer. High-resolution mass spectra were obtained on a Thermo Exactive Plus mass spectrometer using APCI and ESI. Masses were measured using either an Accuris instruments model W3101A-120 or VWR model VWR-224AC balance. For the 24-well tunable photoreactor, the chiller utilized was a Heidolph Hei-CHILL 600 model. For measuring reaction temperatures, a ThermoPro TP19HW waterproof thermometer was utilized.

**Light Sources.** For reactions set up using a Kessil lamp, the Kessil PR160L light (purchased from Kessil: https://kessil.com/products/science_main.php) with wavelength of 390 nm or 456 nm at 100% intensity was used. A Kessil fan rig was used to maintain temperatures at approximately 30 °C. For the white light-mediated reaction, an Agrobrite FLC26D 26-Watt Spiral Compact Fluorescent Grow light bulb was utilized, and a Honeywell 10” black turbo force power table fan was used to prevent reaction from overheating. For reactions with the Analytical Sales photoreactor, the 24-well temperature-controlled reactor (https://www.analytical-sales.com/product-category/photoredox-parallel-synthesis/aluminum-reaction-blocks/temperature-controlled-reactor/1-dram-24-position-tcr/) was used with the chiller temperature set to 15 °C. The light source used for that setup was the Lumidox® II 24-well plate with 18 mm spaced LED arrays at a wavelength of 385 nm (https://www.analytical-sales.com/product/lumidox-ii-24-well-18mm-spacing-led-arrays-with-lens-mat-and-solid base-for-1-dram-tcr/). For all other reactions, the 24-well tunable photoreactor (co-designed with and manufactured by Singh Instruments) was used with the intensity and wavelength specified for each procedure and with the chiller temperature set to 25 °C.

## II. Survey of Current Commercial and Customized Photoreactors

Over the past years, significant advances in photoreactor design have enabled rapid parallel screening and optimization of light-driven transformations.[1,2] Academic, industrial, and commercial efforts have focused on developing photoreactor systems capable of delivering controlled and reproducible irradiation conditions across multiple simultaneous reactions. A few representative examples are listed and described briefly below. It is worth noting that this list is not all inclusive.

**Selected Commercial Photoreactor Examples:**

- **PhotoRedOx Box (HepatoChem)[3]:** A modular mirrored-box photoreactor that uses interchangeable LEDs (365 nm to 808 nm) to irradiate reaction vials of a variety of sizes (0.3 mL to 20.0 mL vials). This setup allows for screening one wavelength and intensity at a time. The PhotoRedOx Box TC enables controlled temperatures between 0 ºC and 80 ºC by connecting to a chiller/heater unit.
- **Illumin8 (Asynt)[4]:** An 8-position parallel photoreactor that uses interchangeable LEDs to irradiate reaction vials of a variety of sizes (1.0 mL to 8.0 mL vials). This setup is compatible with a standard hotplate stirrer for stirring and heating (compatibility up to 80 ºC). This setup allows for screening one wavelength and intensity at a time. The photoreactor can be paired with a cooling base which can be used for temperatures as low as -30 ºC.
- **Integrity 10 Photoreactor (Asynt)[5]:** A compact batch photoreactor that delivers light directly into the reaction medium, providing uniform photon flux throughout the reaction. The reactor allows for easy exchange of the LED to access a wide range of wavelengths (365 nm to 940 nm) and can work with up to ten different wavelengths in parallel. The temperature of the reactions can be individually modulated for each reaction between -30 ºC and 80 ºC.
- **Solstice and Solstice MAXI (Asynt)[6]:** The Solstice series are parallel photoreactors designed for high-throughput reaction screening with temperature control. The solstice accommodates 12 reaction vessels of up to 8 mL, whereas the Solstice MAXI allows for up to 20 mL. The wavelengths accessible are 365, 385, 405, 420, 455, and 525 nm.
- **Lumidox® II LED arrays (Analytical Sales and Services)[7]:** A parallel high-throughput experimentation (HTE) photoreactor supporting 24-, 48-, or 96-well plates with LED panels (255 nm to 730 nm, and white light), adjustable light intensity, and optional cooling for microscale photoredox screening. The single wavelength LED arrays allow screening one wavelength and intensity at a time. However, the discover LED arrays provide the ability to examine multiple wavelengths (up to 4/array) using LED panels with preset wavelengths per column. The temperature-controlled reactors for 24- and 48-well plates provide precise thermal control across the plate and are designed for temperatures between -40 ºC and 82 ºC.
- **PX9 Parallel Screening Photoreactor (Peschl)[8]:** A photoreactor with interchangeable LED cassettes (365 nm to 625 nm) to irradiate reaction vials (0.5 mL to 8.0 mL). One of the LED cassettes allows for wavelength screening by irradiating with 9 different wavelengths (1 wavelength per position) in a single experiment. The intensity of each LED can be adjusted independently. This setup is mounted on a shaker for mixing reaction content and allows for temperature control via a heat transfer fluid system (-20 ºC to 80 ºC).

- **Photochemistry LED Illuminator, PHIL (GSK, Pacer Components Ltd. (now Solsta), and Rosper Engineering Co.)[9]:** A parallel HTE photoreactor supporting 24- and 48-well configurations operating at six different wavelengths (365 nm to 525 nm). The LEDs can be interchanged via cassettes, and one option is the six multiwavelength screening plate which allows screening the six wavelengths in a single experiment (1 wavelength/column).
- **The PhotoCube Plus (ThalesNano)[10]:** A photoreactor with interchangeable LEDs (anywhere between 257 nm to 650 nm) to irradiate 4 reaction vials (4 mL or 30 mL) at up to 4 different wavelengths per experiment. The irradiation intensity, stirring speed, and temperature (10 ºC to 50 ºC) can be adjusted by the user.

**Selected Custom-built Photoreactor Examples:**

- **Chemtech Blue LED Reactor (Novartis)[11]:** An in-house 455 nm LED reactor enabling up to 24 parallel reactions in 1-dram vials with fan cooling for photoredox screening.
- **OSRAM OSLON 455 nm LEDs (AstraZeneca and König)[12]:** A custom-built water-cooled aluminum block for temperature control using a plate of OSRAM OSLON LEDs. Up to six reactions can be examined in 1 experiment using 5 mL vials.
- **Spirochem's Photoreactor (Spirochem)[13]:** Spirochem developed a photoreactor capable of running up to 10 parallel reactions in 5 mL crimp-top vials on a standard stirring plate. Each vial is irradiated with an individual blue LED. Water-cooling and fans help dissipate heat.
- **Boehringer Ingelheim's 3D printed photoreactor (Boehringer Ingelheim)[14]:** Boehringer Ingelheim developed a 3D-printed photoreactor that utilizes Kessil or EvoluChem lamps and can run up to eight parallel reactions or a single reaction (up to 100 mL) using interchangeable vial, flask, and flow holders. The reactor is compatible with standard stirrer hot plates and temperature control between -20 ºC to 80 ºC is achieved through heat sinks, water cooling, and a thermoelectric cooler.
- **Merck's multiuse reactor (Merck & Co.)[15]:** Merck & Co. developed a photoreactor for chemical and biological applications that accommodates up to 24 vials with uniform light distribution and excellent temperature control. The reactor features interchangeable LEDs (455 nm, 555 nm, and 660 nm) and Bluetooth-controlled light intensity and cooling.

## III. Design of the Tunable Photoreactor

### A. Design Strategy

There were three requirements that were considered in designing a 24-well tunable well plate for photochemistry: (1) tunability of wavelengths, intensities, and duration of irradiation; (2) compatibility with existing equipment in synthetic laboratories; and (3) standardization of temperature and stirring.

### B. Design Advances

The 24-well photoreactor was designed to accommodate 1-dram vials and offer enhanced flexibility and control of irradiation for synthetic applications. Each well can be independently

irradiated at distinct wavelengths, UV ($\lambda_{max}$ = 395 nm), blue ($\lambda_{max}$ = 454 nm), green ($\lambda_{max}$ = 525 nm), red ($\lambda_{max}$ = 623 nm), and broad-spectrum white, enabling parallel and modular screening in a single plate. These wavelengths collectively span the spectral range most relevant to photochemical applications and reaction discovery. The inclusion of a broad-spectrum white LED further enhances functionality for exploratory screening. In contrast to fixed monochromatic commercial systems, each well in this platform can be individually programmed to deliver wavelength of irradiation at tunable intensity values (maximum irradiance, 116–720 mW/cm$^2$) which are comparable to or exceed the output of commonly used benchtop photoreactors (Kessil PR160 which operates at an average maximum irradiance of 399 mW/cm$^2$). This contrasts with monochromatic Analytical Sales photoreactors, which operate at a maximum irradiance of 185–530 mW/cm$^2$, thereby expanding the accessible parameter space for low-quantum-yield photochemistry.

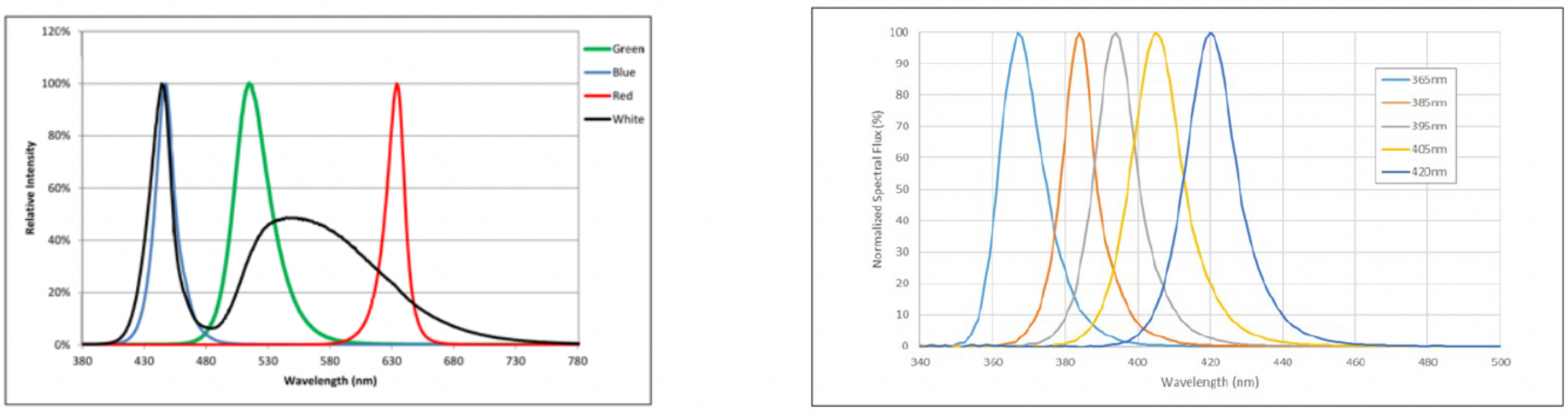


**Figure S1.** Emission spectrum for the RGBW LED (LED) and the UV LED (395 nm).

### C. Plate Design

The well-plate is composed of an aluminum base that provides uniform thermal conductivity across all wells. The base dimensions and vial spacing of the plate are standard microtiter plate sizes, consistent with the commercially available Analytical Sales 24-well plates. This allows for seamless integration with existing high-throughput automation platforms. Reactions can be conducted in a manual format and introduced to the plate with solid or septa caps sealing individual vials. Alternatively, the plate can be sealed using a clamped lid. A custom LED panel equipped with a high-power RGBW array (3.5 mm × 3.5 mm) supplemented with an additional UV LED (3.5 mm × 3.5 mm) at each position was fitted under the 24-well plate. To mitigate overheating of the block, especially upon long periods of irradiation, a cooling block was placed in between the 24-well and the LED panel (Figure S2). The cooling block was integrated to the plate design by passing copper tubing through an aluminum block, which can be connected to a chiller to maintain uniform temperature across the 24-well plate. Additionally, to prevent overheating of the LED panel, a cooling fan at the bottom of the LED panel was installed.

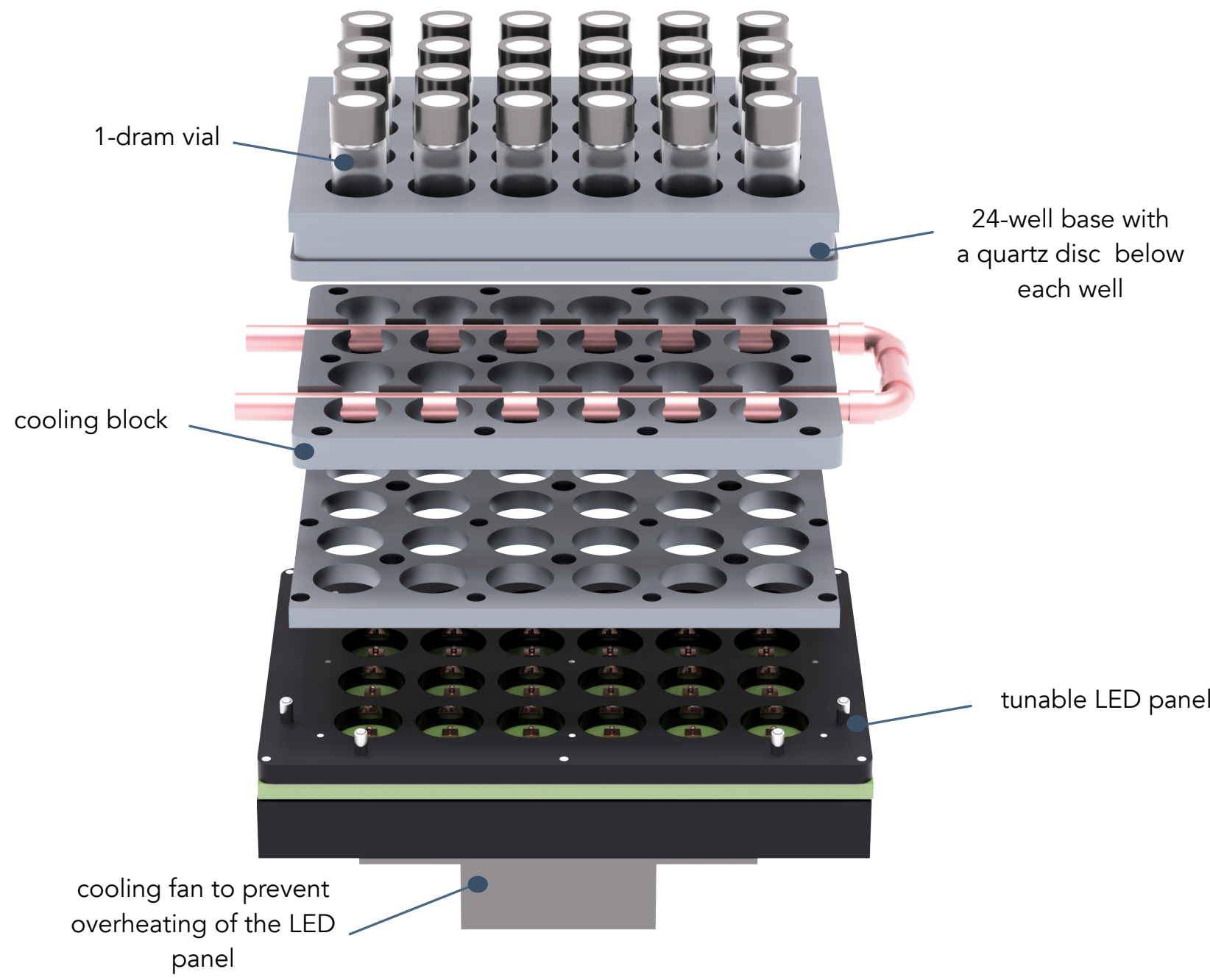


**Figure S2.** Full design scheme of the photoreactor equipped with a tunable LED panel, fan, cooling block, and 24-well base.

### D. Plate Use

To ensure effective stirring, two stirring plates were placed to the left and to the right of the photoreactor and set to 1000 rpm. With this setup, yields comparable to commercial setups were achieved. It is worth noting that with this configuration, the reactions can be conducted on the benchtop sparging with nitrogen without relying on a glovebox for inert atmosphere.

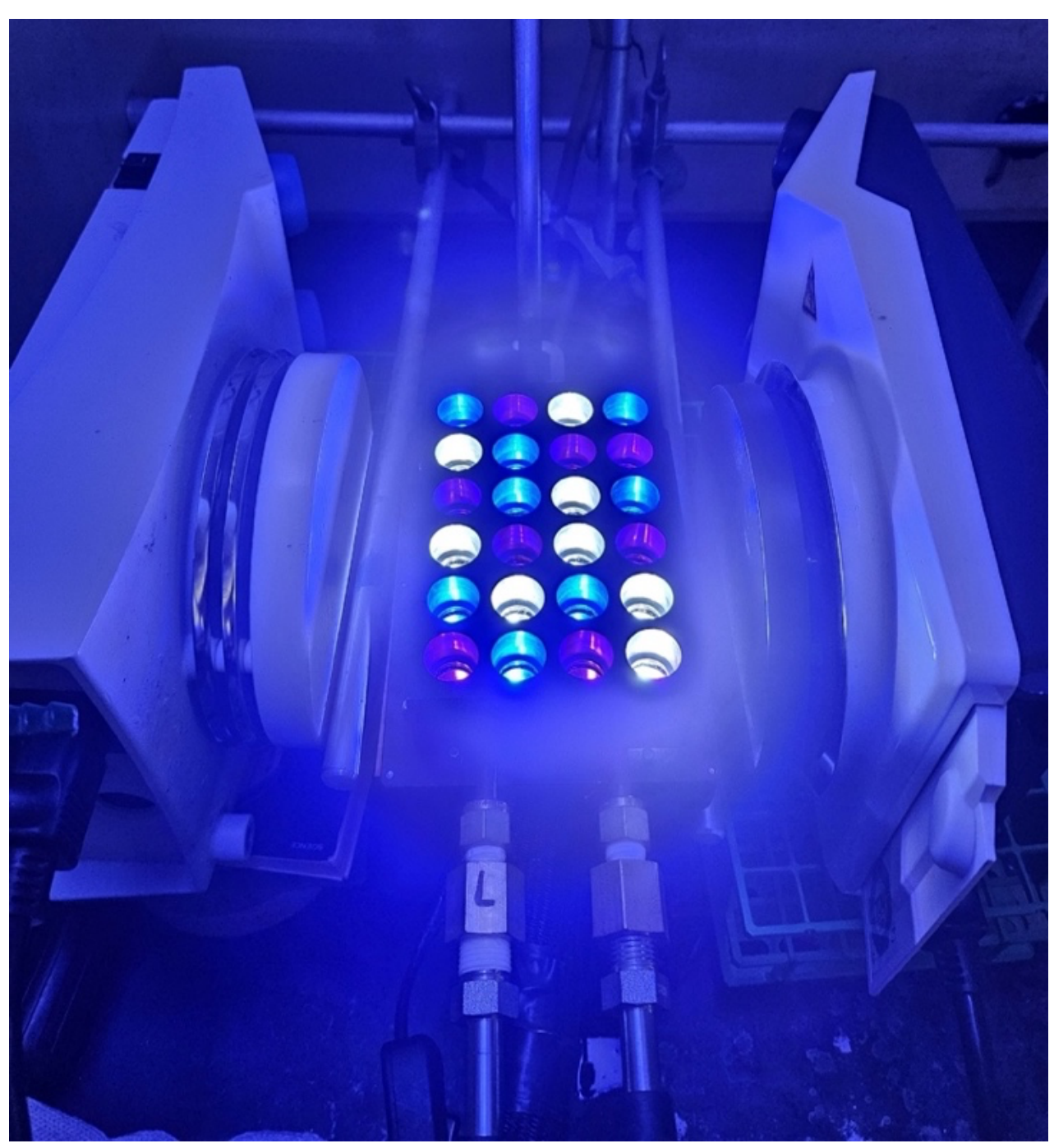

**Figure S3.** Use of stir plates for uniform stirring of reactions in the 24-well photoreactor.

## E. Software Integration

The operation of the LED array is managed by integrated software (compatible with both Mac and Windows) that allows independent programming of the wavelength, intensity, and duration of irradiation on a per-well basis. This is managed through a drag-and-drop workflow builder that allows for the design of multi-step protocols. During operation, a digital display screen provides a real-time visual map of the experiment, including active wells, irradiation settings, and elapsed time for quick monitoring. As different LEDs can be activated individually, simultaneously, or in sequence, the program offers a flexible platform for studying kinetic, mechanistic, and wavelength-dependent phenomena. Methods can also be saved and adapted for each unique experiment.

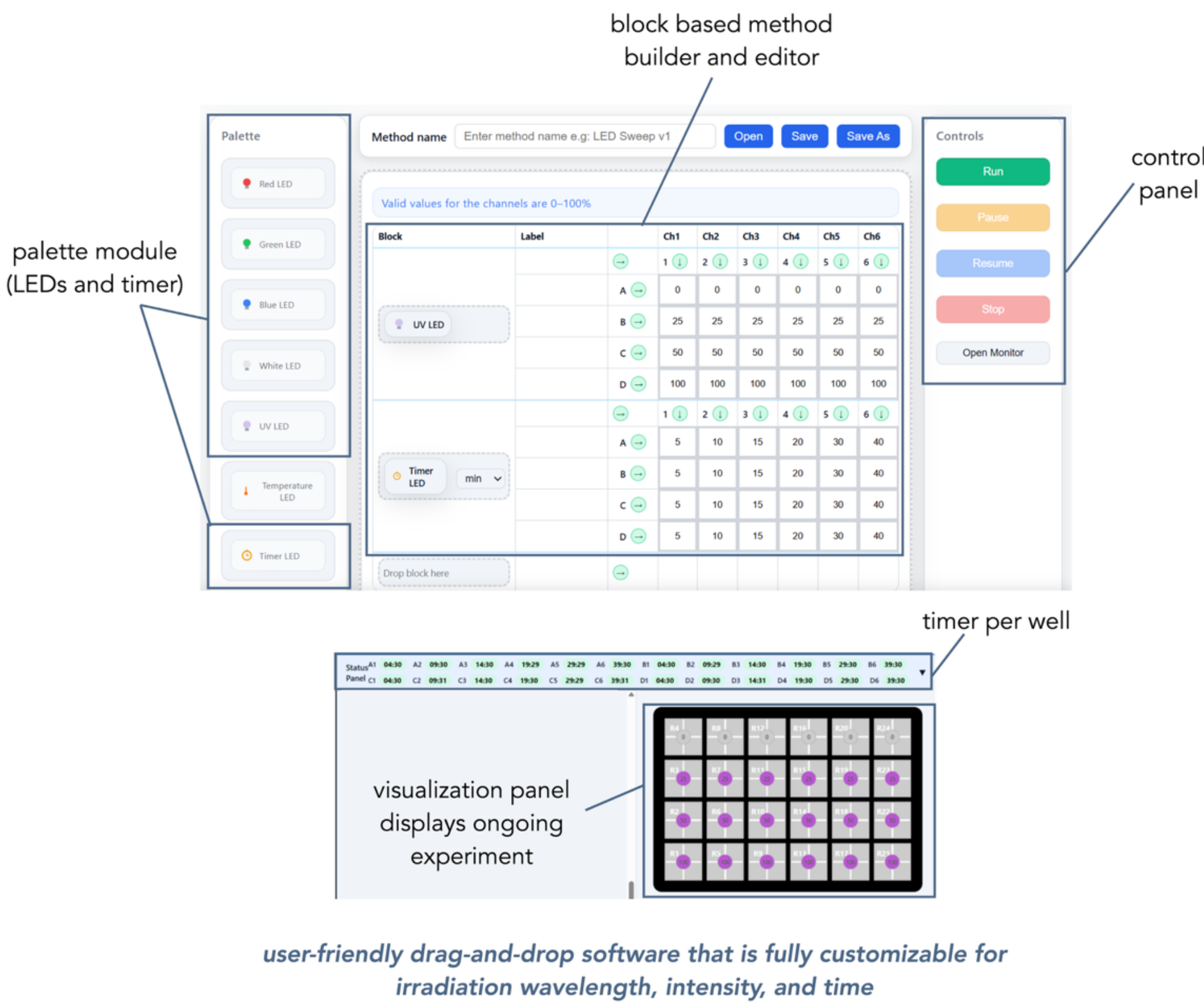


**Figure S4.** Overview of the software program used to control the LED panel of the 24-well tunable photoreactor. This software provides a user-friendly drag-and-drop interface to program irradiation wavelength, intensity, and time on a per-well basis.

## F. Example Procedure for Building a Method Using This Software

Goal: Run an experiment where the reactions are exposed to varied intensity and different reaction times using blue LEDs.

Method Building Instructions:

1. From the palette module on the left, drag a blue LED block into the method builder and editor section. This will open a 24-well block.
2. In the channel columns and rows of this 24-well block, enter the intensity values as required per each position (values range from 0–100%).
3. From the palette module, drag the “timer LED” block below the Blue LED block.

4. Set the timer unit to second (s), minute (min), or hour (h).
5. Enter the desired irradiation time per channel (for example, Ch1A: 5, Ch2A: 10, Ch3A: 30, etc).
6. Click Run in the control panel to execute this method. Use Pause, Resume, or Stop if adjustments are needed mid-experiment.IV. Experimental Procedures Using the Tunable Photoreactor

## A. Standardization and Reproducibility

### A1. Procedure for Evaluating Temperature Upon Irradiation of Solvent in the Tunable Photoreactor

A series of 24 vials were irradiated concurrently. The 1-dram vials were equipped with a PTFE-coated stir bar and charged with acetonitrile (2.00 mL). The vials were fitted into the base of the photoreactor which was connected to a chiller with the temperature set to 25 °C. The reactions were irradiated using the blue LEDs at 100% intensity. The stirrers on each side of the plate were set at 1000 rpm to ensure efficient stirring at the corners and the center of the plate (Figure S3). The temperature of the solution was recorded over 6 h using a digital thermometer.

**Table S1.** Summary of temperature of MeCN solution at different time points upon irradiation using the blue LED in the tunable photoreactor

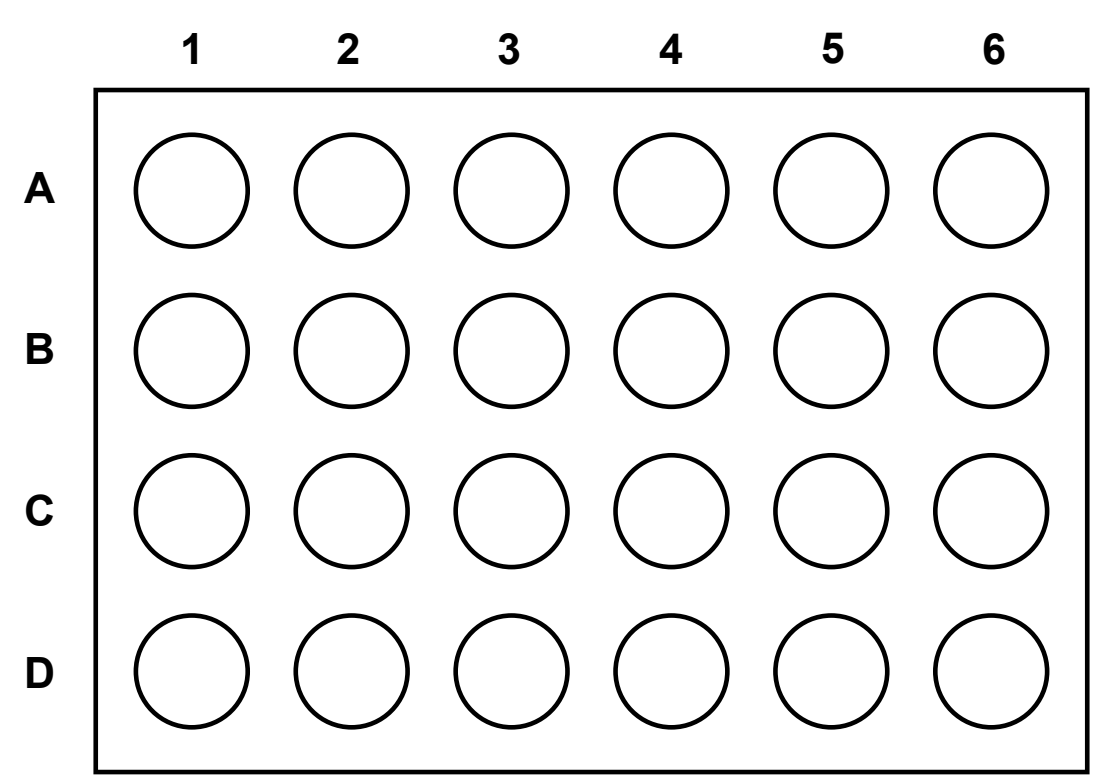

| Position | 0h | 0.5h | 1h | 1.5h | 2 h | 4h | 6h |
|---|---|---|---|---|---|---|---|
| A1 | 23.4 | 29.5 | 30.2 | 30.3 | 29.4 | 29.9 | 29.8 |
| A2 | 23.4 | 29.7 | 30.1 | 30.2 | 29.3 | 30.2 | 29.8 |
| A3 | 23.4 | 29.7 | 30.3 | 30.5 | 29.9 | 30.2 | 30.2 |
| A4 | 23.4 | 29.9 | 30.4 | 30.8 | 30.2 | 30.1 | 30.5 |
| A5 | 23.4 | 29.8 | 30.5 | 30.8 | 30.0 | 30.3 | 30.4 |
| A6 | 23.4 | 29.8 | 30.6 | 30.6 | 29.9 | 30.5 | 30.5 |
| B1 | 23.4 | 29.3 | 30.7 | 30.2 | 29.3 | 30.0 | 29.9 |
| B2 | 23.4 | 29.5 | 30.5 | 30.3 | 29.5 | 30.2 | 30.0 |
| B3 | 23.4 | 29.7 | 30.6 | 30.5 | 29.6 | 30.5 | 30.5 |
| B4 | 23.4 | 29.8 | 30.8 | 30.6 | 29.8 | 30.6 | 30.5 |
| B5 | 23.4 | 29.6 | 30.3 | 30.9 | 29.3 | 30.5 | 30.4 |
| B6 | 23.4 | 29.9 | 30.5 | 30.4 | 29.8 | 30.4 | 30.2 |
| C1 | 23.4 | 29.5 | 30.6 | 30.0 | 29.2 | 29.7 | 29.8 |
| C2 | 23.4 | 29.7 | 30.2 | 30.2 | 29.4 | 29.9 | 30.1 |
| C3 | 23.4 | 29.8 | 30.5 | 30.5 | 29.5 | 30.2 | 30.2 |
| C4 | 23.4 | 29.9 | 30.6 | 30.4 | 29.7 | 30.4 | 30.3 |
| C5 | 23.4 | 29.9 | 30.6 | 30.5 | 29.9 | 30.2 | 30.2 |
| C6 | 23.4 | 30.0 | 30.6 | 30.2 | 29.9 | 30.5 | 30.4 |
| D1 | 23.4 | 29.4 | 30.1 | 29.7 | 28.9 | 29.6 | 29.6 |
| D2 | 23.4 | 29.6 | 30.4 | 30.0 | 29.4 | 30.1 | 29.9 |
| D3 | 23.4 | 29.6 | 30.7 | 30.4 | 29.5 | 30.0 | 29.9 |
| D4 | 23.4 | 29.8 | 30.5 | 30.5 | 29.8 | 30.2 | 30.1 |
| D5 | 23.4 | 30.1 | 30.5 | 30.2 | 29.9 | 29.9 | 30.0 |
| D6 | 23.4 | 29.8 | 30.5 | 30.2 | 29.6 | 29.8 | 29.9 |

## A2. Procedure for Evaluating Robustness of Stirring for the Tunable Photoreactor

### A2.1. Procedure for Evaluating Effect of Stir Bar Sizes

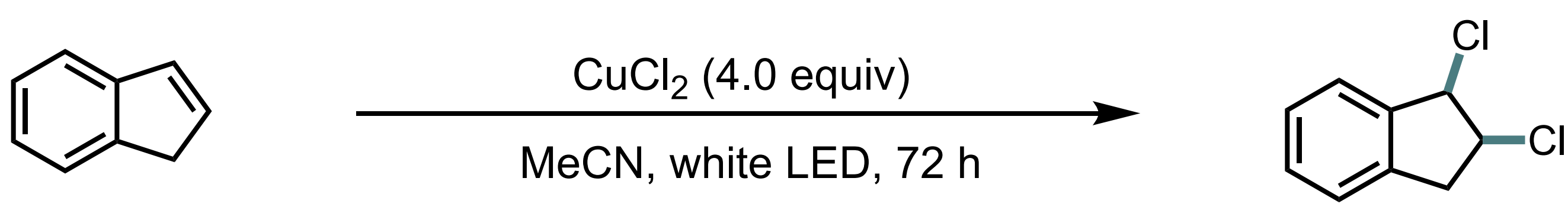


A series of 5 reactions was conducted concurrently according to the general procedure for Cu–mediated dichlorination (IV.B5). To evaluate the robustness of stirring and reproducibility for a heterogeneous reaction, 5 different stir bar sizes were employed (Figure S5) with the stir plate configuration utilized for the tunable photoreactor (Figure S3). While SB3 and SB4 appeared to stir most vigorously and uniformly, effective stirring was still achieved with all stir bar selections.

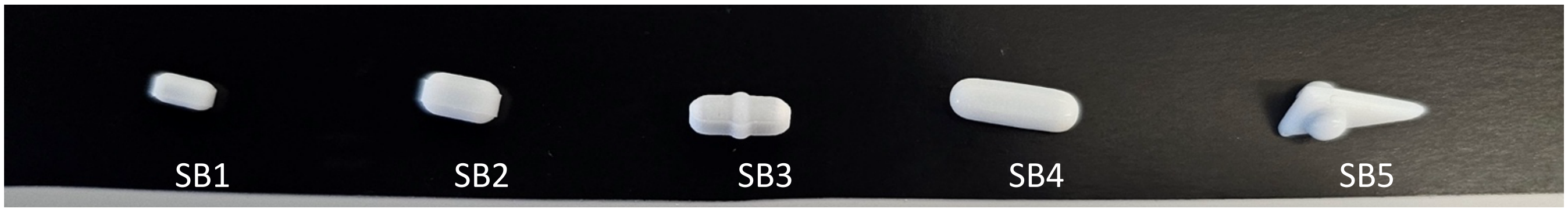


**Figure S5.** Sizes of the 5 stir bars chosen for the reproducibility and standardization of stirring experiment. Dimensions of the stir bars were the following: SB1 (5 x 1.5 mm), SB2 (6 x 3 mm), SB3 (8 x 3 mm), SB4 (10 x 3 mm), and SB5 (11 x 4 x 4.5 mm). SB4 is the stir bar size that was typically used for all experiments with the tunable photoreactor.

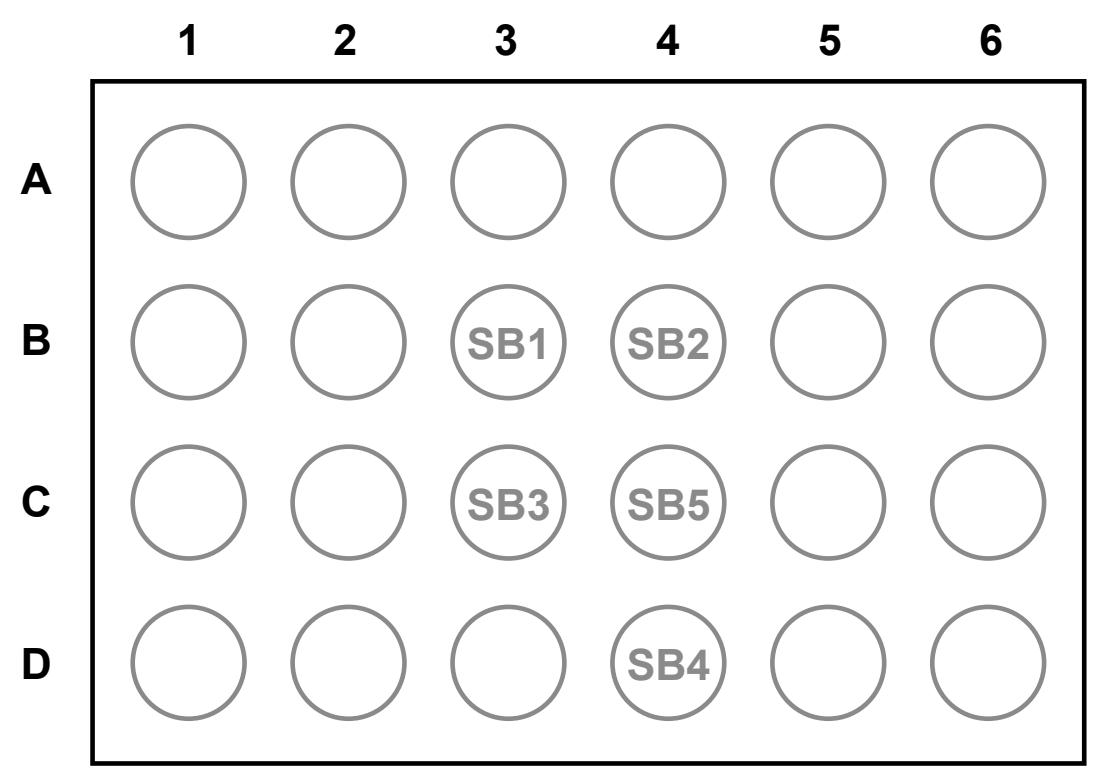


| Stir Bar Used | Reaction Yield (%) |
|---|---|
| SB1 | 62 |
| SB2 | 75 |
| SB3 | 68 |
| SB4 | 71 |
| SB5 | 70 |

**Figure S6.** Plate design and summary of reaction yields using different sized stir bars.

### A2.2. Procedure for Evaluating Effect of Reaction Volume

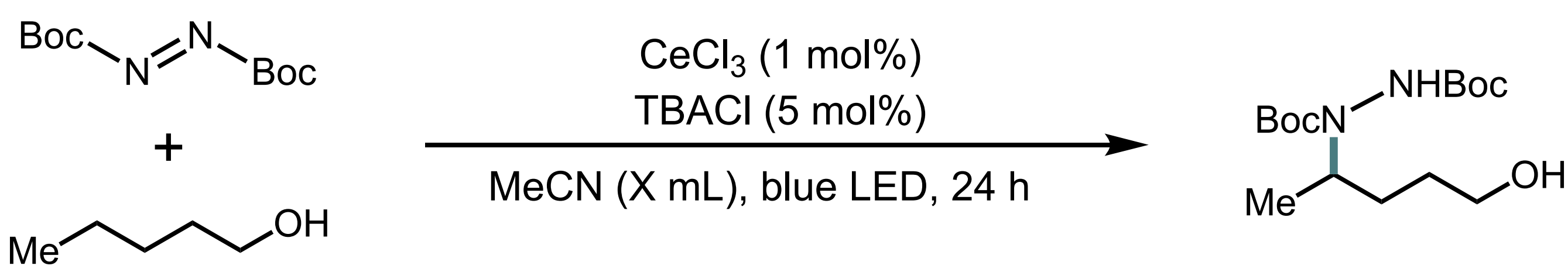


A series of 5 reactions was conducted concurrently according to the general procedure for Ce–catalyzed C–H amination (IV.B4). This experiment evaluated the robustness of the plate at different concentrations of a homogeneous reaction. The volume of the reactions were varied while maintaining a molarity of 0.1 M and using the stir plate configuration utilized for the tunable photoreactor (Figure S3).

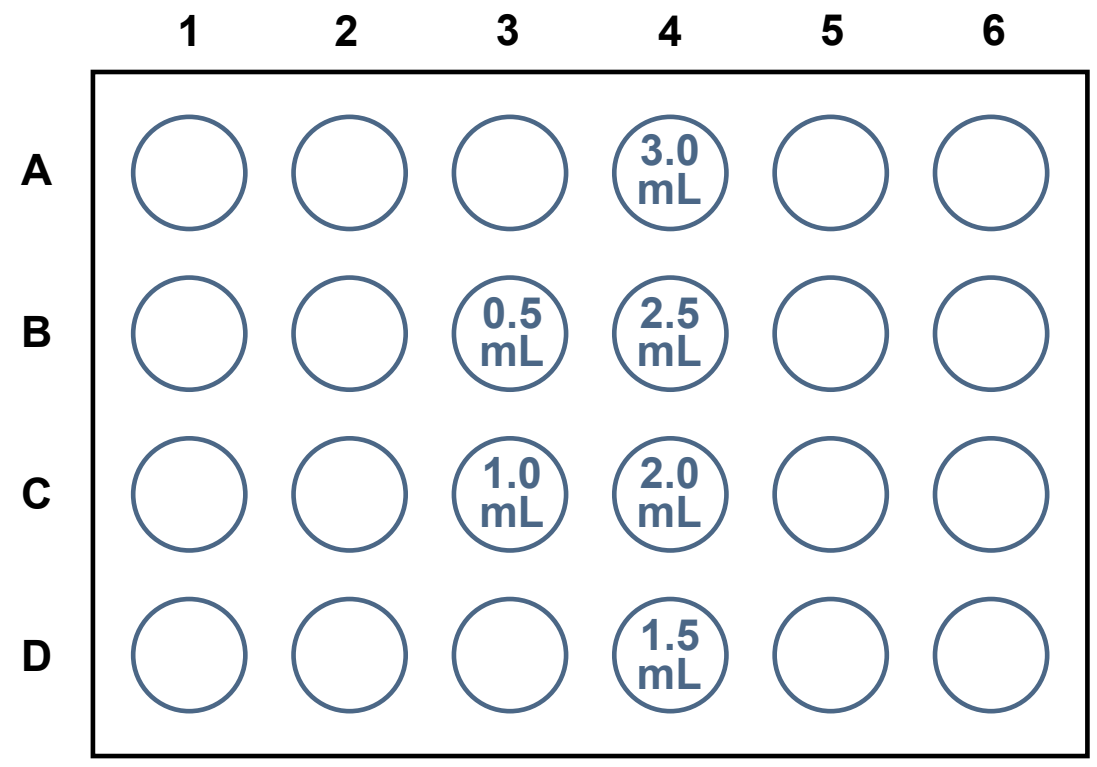


| Volume of Reaction | Reaction Yield (%) |
|---|---|
| 0.5 mL | 72 |
| 1.0 mL | 80 |
| 1.5 mL | 83 |
| 2.0 mL | 85 |
| 2.5 mL | 83 |
| 3.0 mL | 86 |

**Figure S7.** Plate design and summary of reaction yields using different reaction volumes for Ce–catalyzed C–H amination.

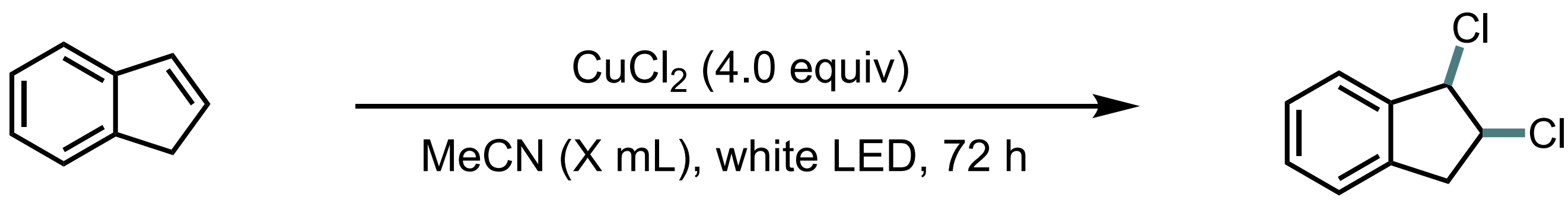


A series of 5 reactions was conducted concurrently according to the general procedure for Cu–mediated dichlorination (IV.B5). The volume was varied while keeping the original reaction concentration constant (0.04 M). This was examined to evaluate the robustness of stirring with the stir plate configuration utilized for the tunable photoreactor (Figure S3). This reaction is heterogeneous in nature.

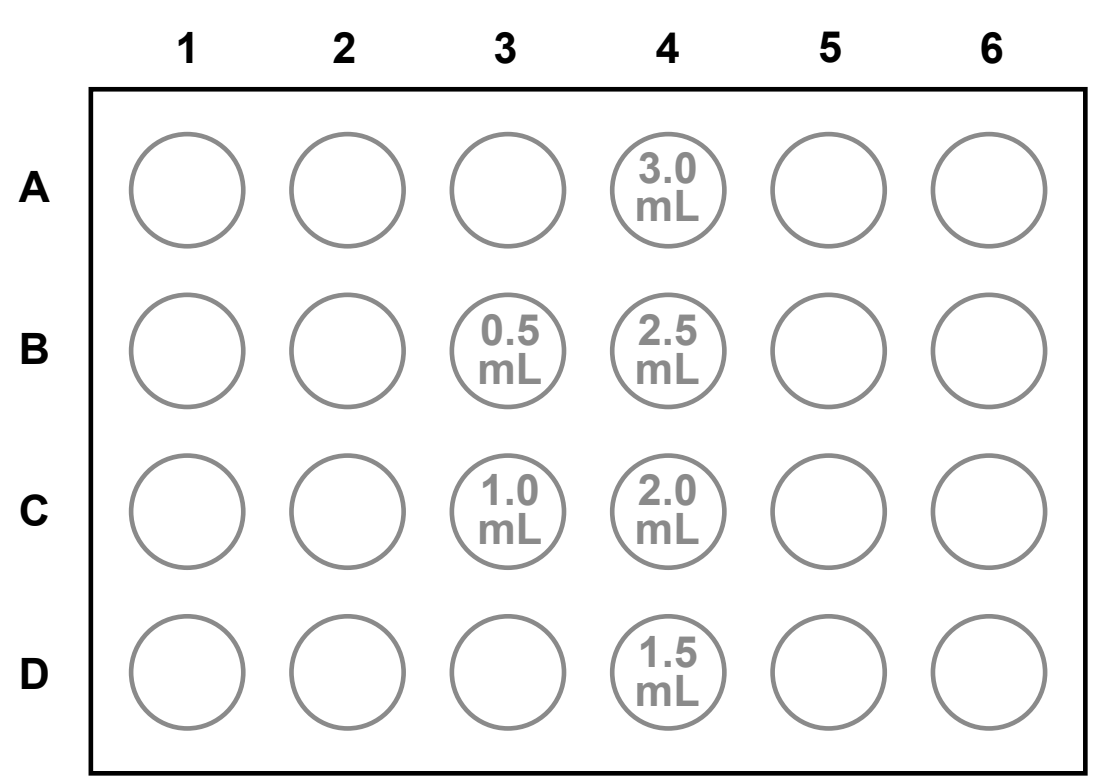


| Volume of Reaction | Reaction Yield (%) |
|---|---|
| 0.5 mL | 52 |
| 1.0 mL | 56 |
| 1.5 mL | 60 |
| 2.0 mL | 64 |
| 2.5 mL | 71 |
| 3.0 mL | 68 |

**Figure S8.** Plate design and summary of reaction yields using different reaction volumes for the Cu–mediated dichlorination reaction.

### A3. Procedure for Reproducing the Ce–Catalyzed C–H Amination

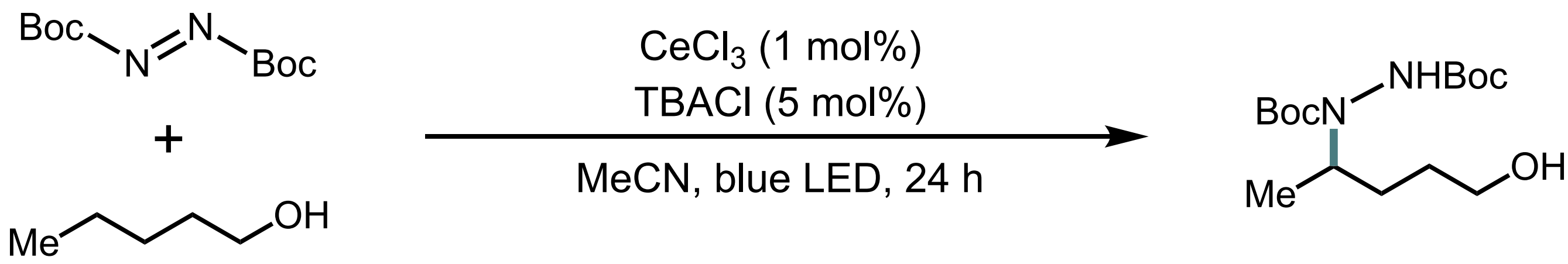


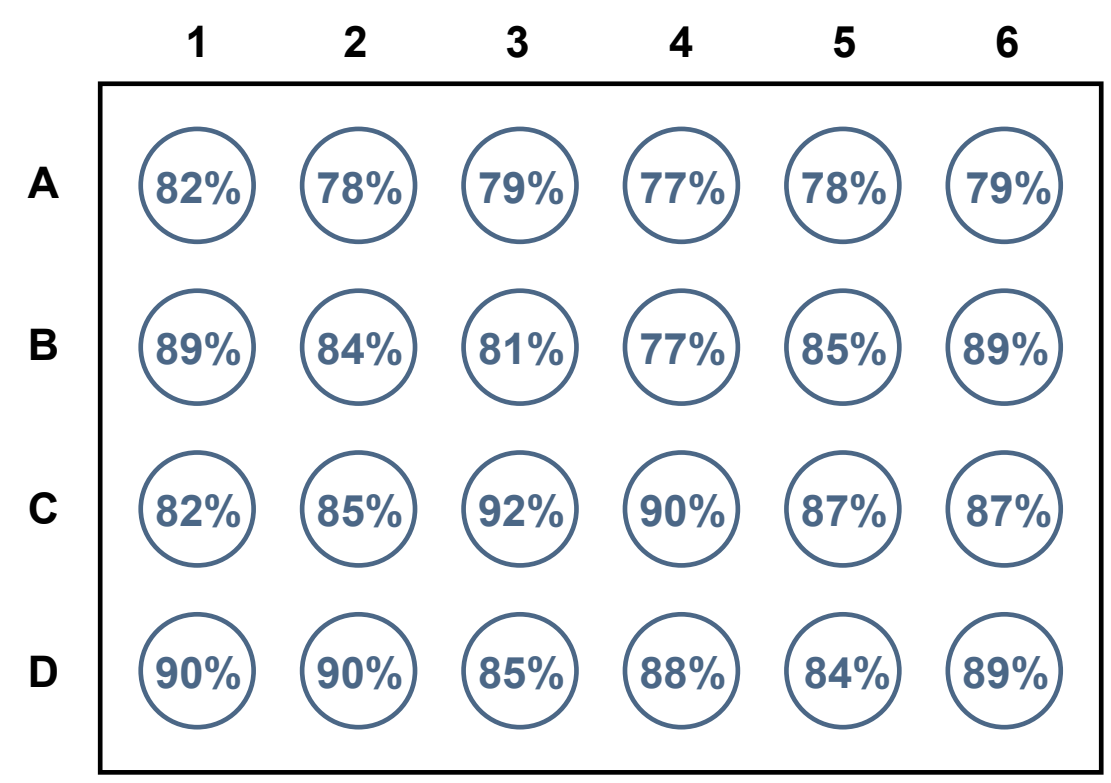


Average yield ± std = 84% ± 5%
(n = 24)

**Figure S9.** Summary of the results obtained using the tunable photoreactor for the reproduced Ce–catalyzed C–H amination.

A series of 24 reactions was conducted concurrently according to the general procedure for Ce–catalyzed C–H amination (IV.B4). This was a rerun of the Ce–Catalyzed C–H Amination in the tunable photoreactor to demonstrate robustness of the tunable photoreactor and its reproducibility across identical plates.

## B. Benchmarking and Comparison to Conventional Light Sources

### B1. Procedure for Fe–Catalyzed Decarboxylative Giese Addition Using Kessil Lamp

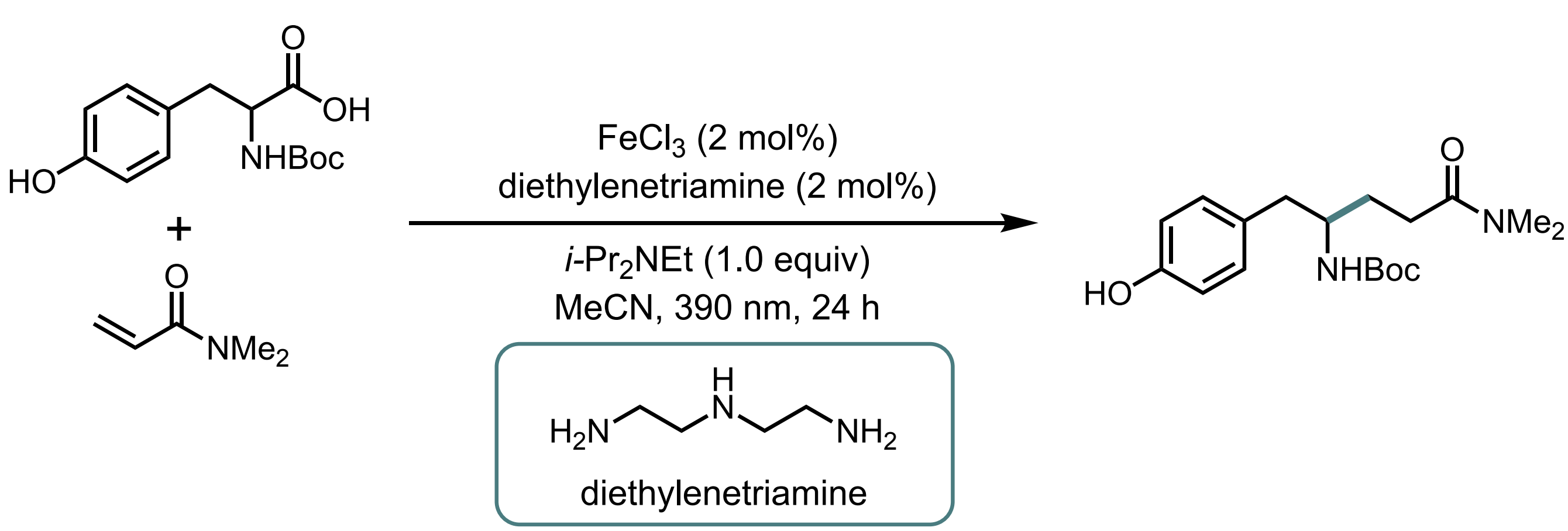

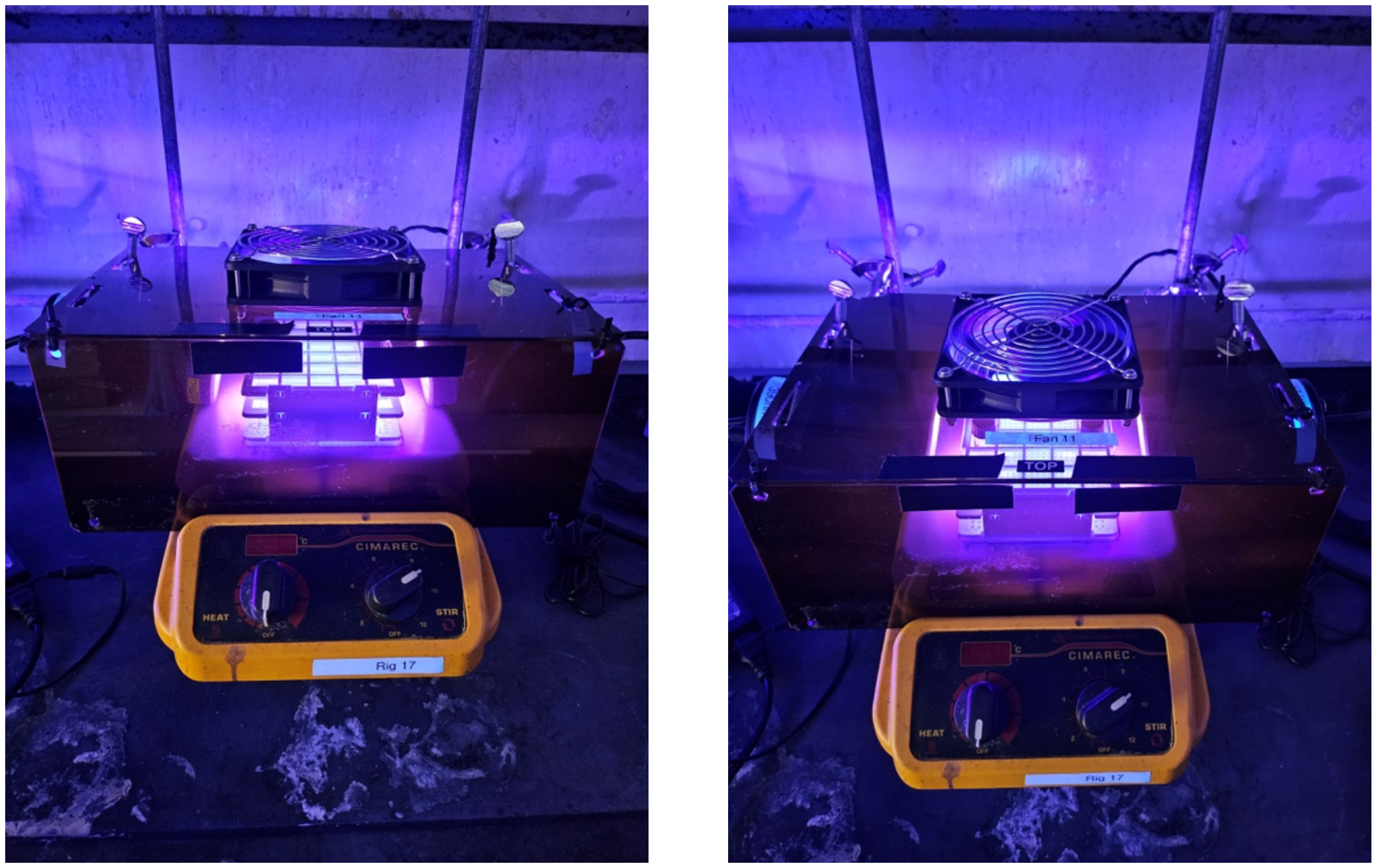

**Figure S10.** Kessil lamp (390 nm) rig setup for reaction screening.

**Table S2.** Reaction yields for the 10 individual Fe–catalyzed reactions using the 390 nm Kessil lamps.

| Reaction | Reaction yield (%) | Temperature (ºC) |
|---|---|---|
| Reaction 1 | 81 | 34.8 |
| Reaction 2 | 86 | 33.5 |
| Reaction 3 | 86 | 34.1 |
| Reaction 4 | 87 | 33.1 |
| Reaction 5 | 72 | 35.0 |
| Reaction 6 | 89 | 33.6 |
| Reaction 7 | 85 | 32.1 |
| Reaction 8 | 86 | 32.2 |
| Reaction 9 | 89 | 33.9 |
| Reaction 10 | 80 | 33.2 |

Average yield ± std = 80% ± 8%
Average temperature ± std = 33.5 °C ± 1 °C
(n = 10)

A series of 10 reactions was conducted concurrently and spread over 5 rigs as shown in Figure S10. A representative procedure for each reaction is communicated below:

A 1-dram vial equipped with a PTFE-coated stir bar was charged with dry acetonitrile (1.80 mL). To the reaction vial, Boc-Tyr-OH (30.9 mg, 0.110 mmol, 1.10 equiv) was added. This was followed by addition of Fe salt (0.00200 mmol, 0.0200 equiv) and ligand (0.00200 mmol, 0.0200 equiv), both as 0.0200 M stock solutions in dry acetonitrile. Subsequently, *N,N*-

diisopropylethylamine (17.4 μL, 0.100 mmol, 1.00 equiv) and *N,N*-dimethylacrylamide (10.3 μL, 0.100 mmol, 1.00 equiv) were added. The vial was fitted with a septum cap, and a 21-gauge needle connected to a positive pressure of nitrogen was submerged in the solution along with another 21-gauge needle vent to allow the reaction to sparge for 3 min. The needles were removed, and the septum cap was wrapped with electrical tape to ensure sealing. The vial was placed about 4 cm away from a 390 nm Kessil lamp and irradiated for 24 h while stirring at 800 rpm. A Kessil fan rig was used to prevent reactions from overheating. At the end of each reaction, the temperature of the reaction was recorded using a digital thermometer. After 24 h, the reaction was diluted with EtOAc, filtered through a plug of silica eluting with EtOAc, and concentrated under reduced pressure. The crude reaction mixture was dissolved in $CDCl_3$ (1.00 mL) and $CH_2Br_2$ (3.50 μL, 0.0500 mmol) was added as an external standard. The yield of the desired product was analyzed using $^1$H NMR spectroscopy.

### B2. Procedure for Fe–Catalyzed Decarboxylative Giese Addition Using the Analytical Sales Photoreactor

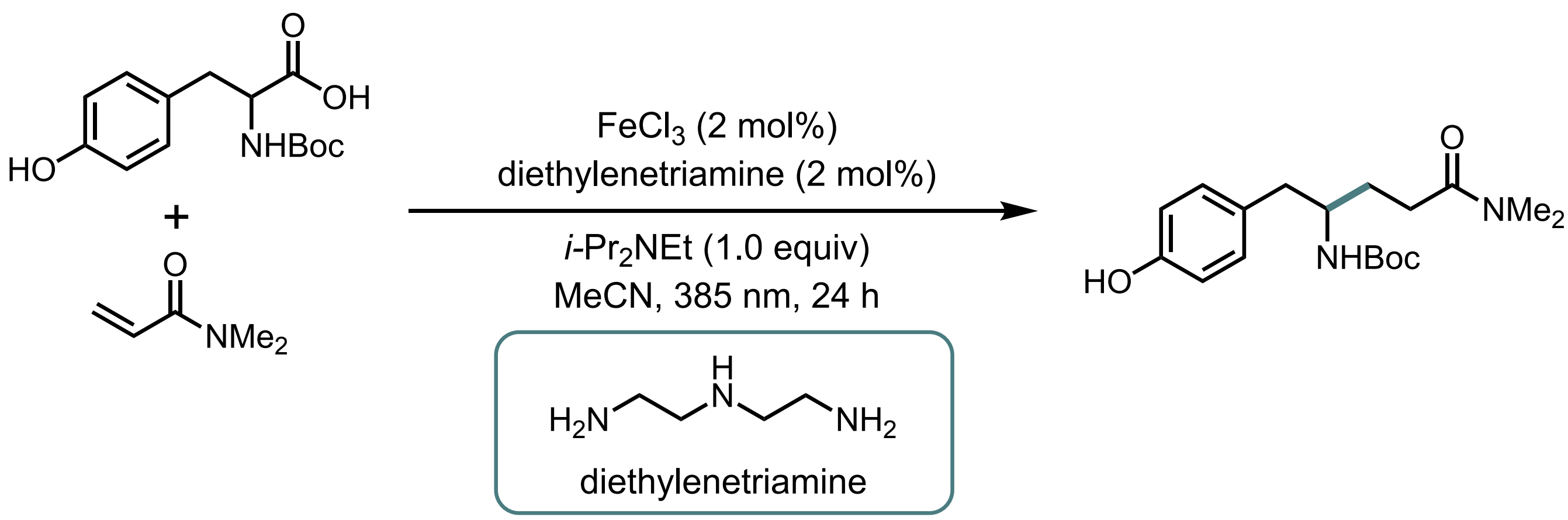


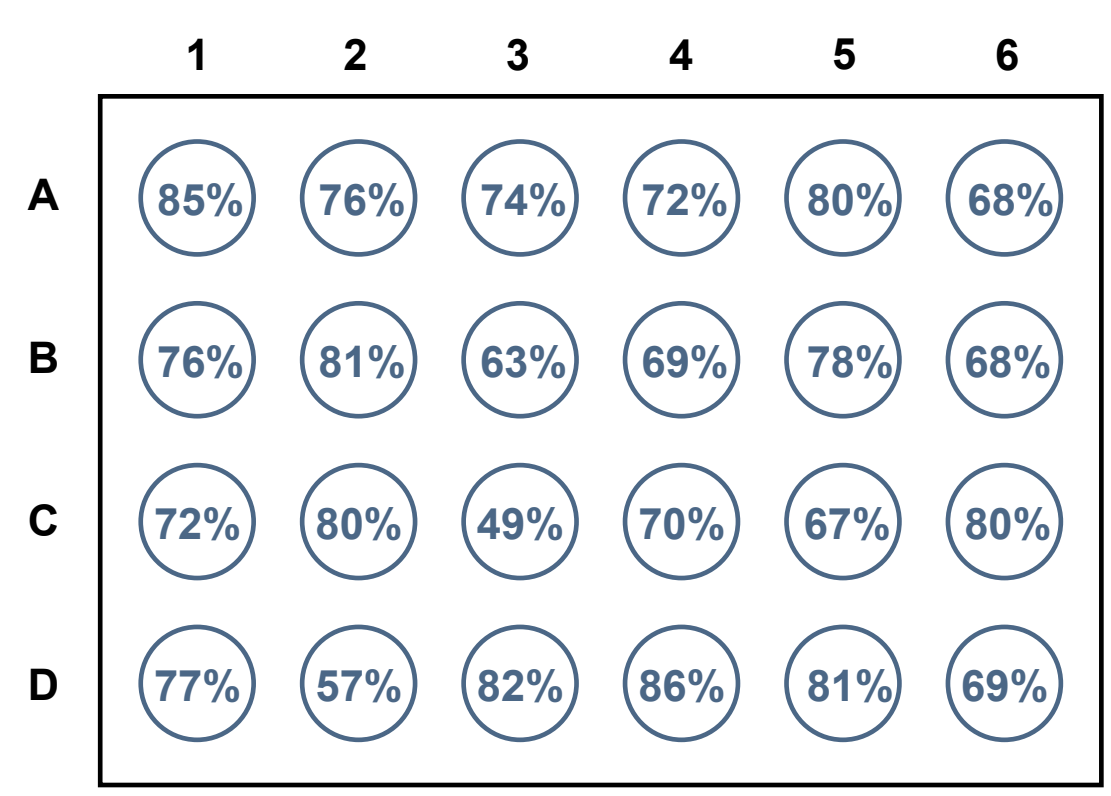


Average yield ± std = 73% ± 9%
(n = 24)

**Figure S11.** Summary of the results obtained using the Analytical Sales photoreactor for the Fe–catalyzed decarboxylative Giese addition reaction.

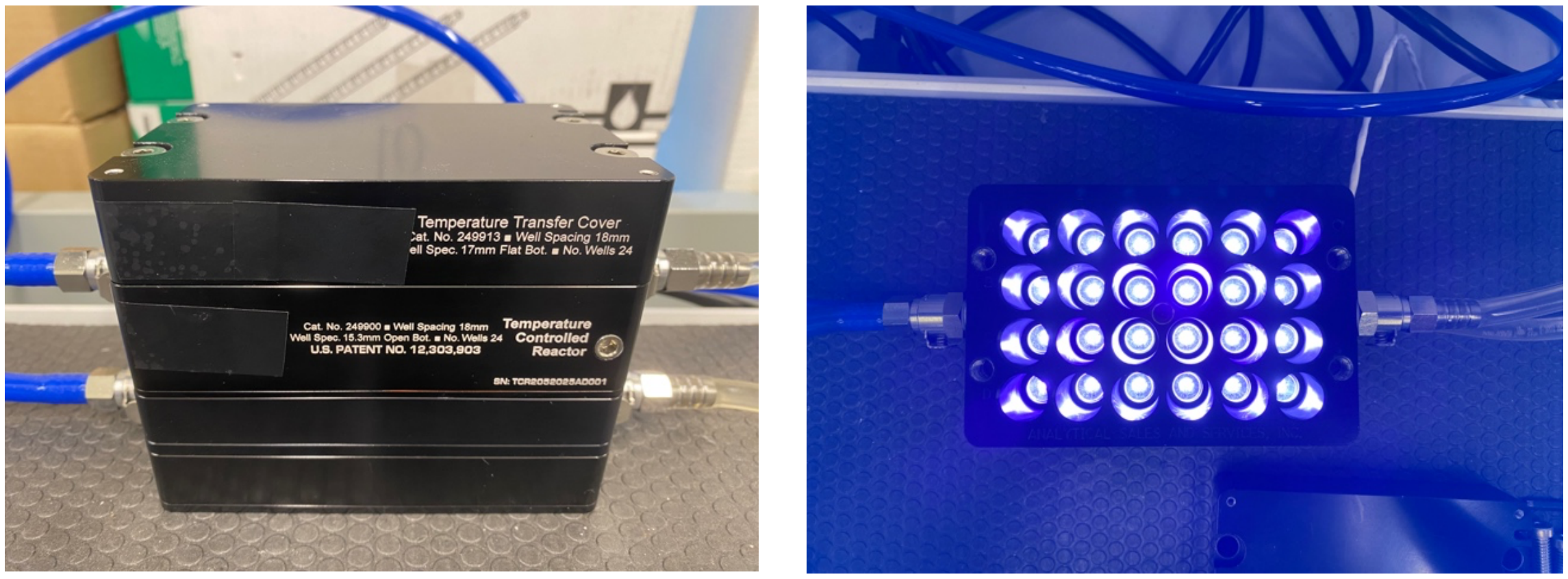


**Figure S12.** Analytical Sales (385 nm) temperature-controlled reactor setup for reaction screening.

A series of 24 reactions was conducted concurrently. A representative reaction procedure for each reaction is communicated below:

To a 1-dram vial equipped with a PTFE-coated stir bar was added Boc-Tyr-OH (30.9 mg, 0.110 mmol, 1.10 equiv) and dry acetonitrile (1.80 mL). This was followed by adding Fe salt (0.00200 mmol, 0.0200 equiv) and ligand (0.00200 mmol, 0.0200 equiv), both as 0.0200 M stock solutions in dry acetonitrile. Subsequently, *N,N*-diisopropylethylamine (17.4 $\mu$L, 0.100 mmol, 1.00 equiv) and *N,N*-dimethylacrylamide (10.3 $\mu$L, 0.100 mmol, 1.00 equiv) were added. The vial was fitted with a septum cap, and a 21-gauge needle connected to a positive pressure of nitrogen was submerged in the solution along with another 21-gauge needle vent to allow the reaction to sparge for 3 min. The needles were removed, and the septum cap was wrapped with electrical tape to ensure sealing. The reaction was placed in the 24-well temperature-controlled reactor with the chiller set to 15 °C and irradiated using the 385 nm LED panel at full intensity for 24 h. A shaker at 400 rpm was utilized for mixing the reaction. At the end of each reaction, the temperature of the reaction was recorded using a digital thermometer. After 24 h, the reaction was diluted with EtOAc, filtered through a plug of silica eluted with EtOAc, and concentrated under reduced pressure. The crude reaction mixture was dissolved in $CDCl_3$ (1.00 mL) and $CH_2Br_2$ (3.50 $\mu$L, 0.0500 mmol) was added as an external standard. The yield of the desired product was analyzed using $^1$H NMR spectroscopy.

**Table S3.** Summary of reaction yield and temperature of reaction after 24 h upon irradiation using the blue LED in the Analytical Sales Photoreactor.

| Position | Reaction yield (%) | Temperature (ºC) |
|---|---|---|
| A1 | 85 | 20.9 |
| A2 | 76 | 20.5 |
| A3 | 74 | 20.6 |
| A4 | 72 | 20.7 |
| A5 | 80 | 20.8 |
| A6 | 68 | 20.7 |
| B1 | 76 | 20.8 |
| B2 | 81 | 20.6 |
| B3 | 63 | 20.5 |
| B4 | 69 | 20.4 |
| B5 | 78 | 20.5 |
| B6 | 68 | 20.9 |
| C1 | 72 | 20.8 |
| C2 | 80 | 20.6 |
| C3 | 49 | 20.4 |
| C4 | 70 | 20.5 |
| C5 | 67 | 20.5 |
| C6 | 80 | 20.6 |
| D1 | 77 | 21.0 |
| D2 | 57 | 20.8 |
| D3 | 82 | 20.7 |
| D4 | 86 | 20.7 |
| D5 | 81 | 20.6 |
| D6 | 69 | 21.0 |

Average yield ± std = 73% ± 9%
Average temperature ± std = 20.7 °C ± 0.2 °C
(n = 24)

## B3. Procedure for Fe–Catalyzed Decarboxylative Giese Addition Using the Tunable Photoreactor

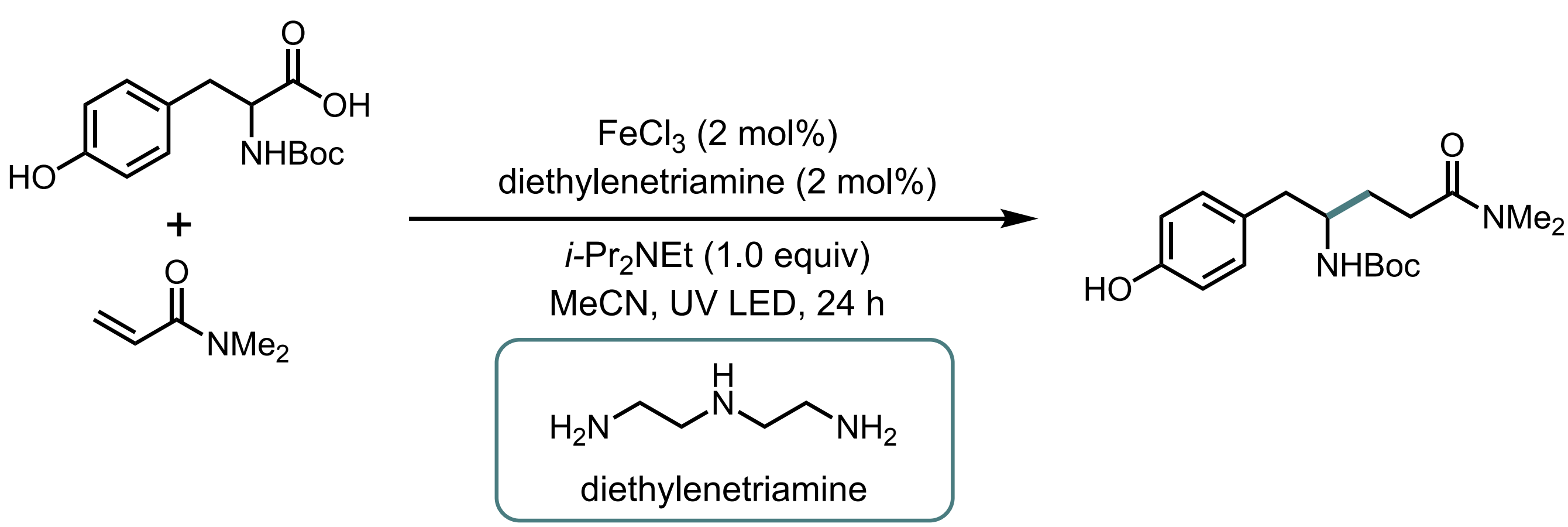

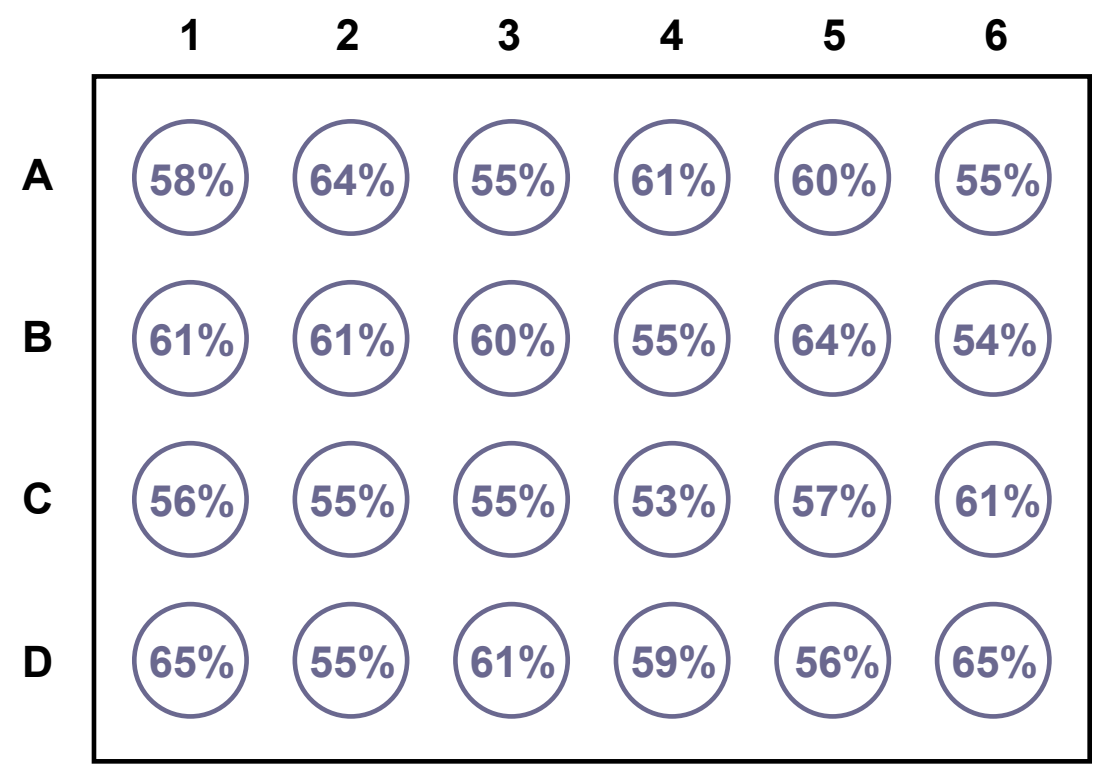


Average yield ± std = 58% ± 4%
(n = 24)

**Figure S13.** Summary of the results obtained using the photoreactor for the Fe–catalyzed decarboxylative Giese addition reaction.

A series of 24 reactions was conducted concurrently. A representative procedure for each reaction is communicated below:

A 1-dram vial equipped with a PTFE-coated stir bar was charged with dry acetonitrile (1.80 mL). To the reaction vial, Boc-Tyr-OH (30.9 mg, 0.110 mmol, 1.10 equiv) was added. This was followed by adding Fe salt (0.00200 mmol, 0.0200 equiv) and ligand (0.00200 mmol, 0.0200 equiv), both as 0.0200 M stock solutions in dry acetonitrile. Subsequently, *N,N*-diisopropylethylamine (17.4 $\mu$L, 0.100 mmol, 1.00 equiv) and *N,N*-dimethylacrylamide (10.3 $\mu$L, 0.100 mmol, 1.00 equiv) were added. The vial was fitted with a septum cap, and a 21-gauge needle connected to a positive pressure of nitrogen was submerged in the solution along with another 21-gauge needle vent to allow the reaction to sparge for 3 min. The needles were removed, and the septum cap was wrapped with electrical tape for sealing. The vial was fitted into the base of the photoreactor which was connected to a chiller with the temperature set to 25 °C. The reactions were irradiated at 395 nm at 100% intensity. The stirrers on each side of the plate were set at 1000 rpm to ensure efficient stirring at the corners and the center of the plate (Figure S3). After 24 h, the reaction was diluted with EtOAc, filtered through a plug of silica eluting with EtOAc, and concentrated under reduced pressure. The crude reaction mixture was dissolved in $CDCl_3$ (1.00 mL) and $CH_2Br_2$ (3.50 $\mu$L, 0.0500 mmol) was added as an external standard. The yield of the desired product was analyzed using $^1$H NMR spectroscopy.

### B4. Procedure for Ce–Catalyzed C–H Amination

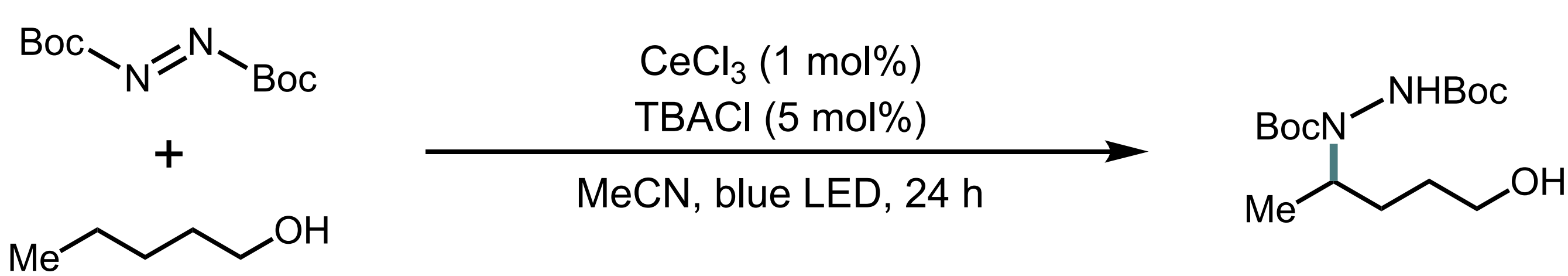

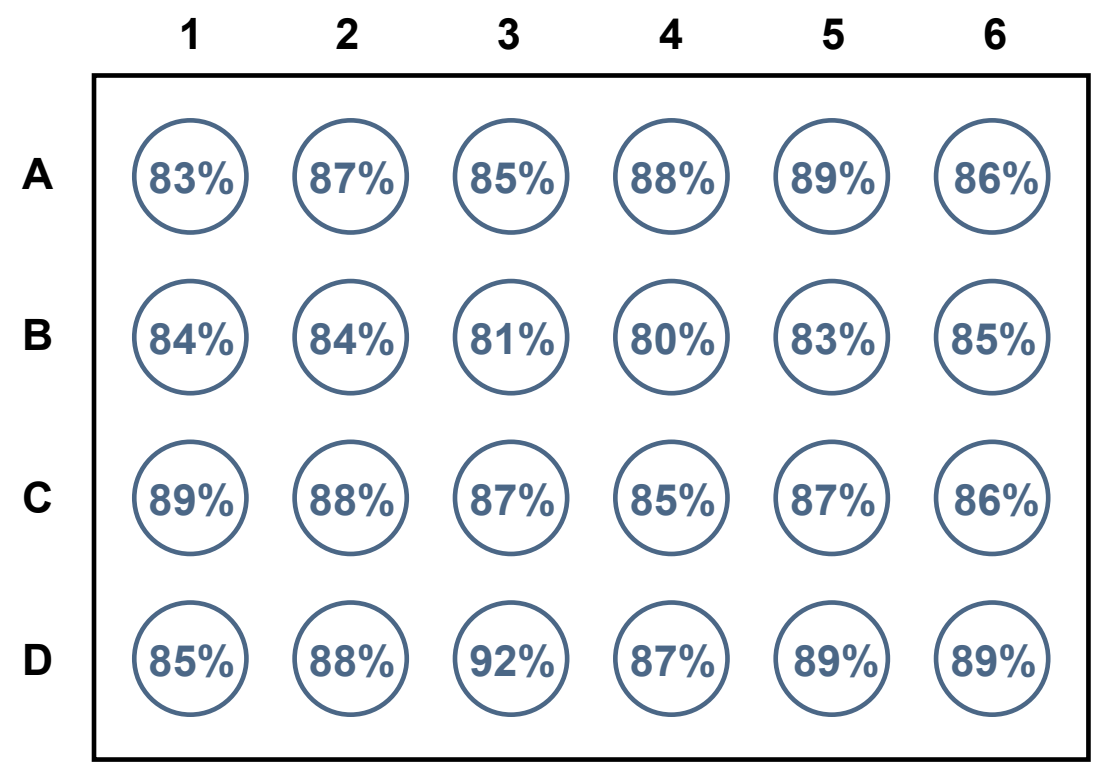


Average yield ± std = 86% ± 3%
(n = 24)

**Figure S14.** Summary of the results obtained using the tunable photoreactor for the Ce–catalyzed C–H amination reaction.

A series of 24 reactions was conducted concurrently. A representative reaction procedure for each reaction is communicated below:

To a 1-dram vial equipped with a PTFE-coated stir bar was added $CeCl_3$ (0.500 mg, 0.00200 mmol, 0.0100 equiv), tetrabutylammonium chloride (2.80 mg, 0.0100 mmol, 0.0500 equiv), di-*tert*-butyl azodicarboxylate (46.0 mg, 0.2000 mmol, 1.00 equiv), and 1-pentanol (64.5 $\mu$L, 0.600 mmol, 3.00 equiv) followed by 2.00 mL of MeCN. The vial was fitted with a septum cap, and a 21-gauge needle connected to a positive pressure of nitrogen was submerged in the solution along with another 21-gauge needle vent to allow the reaction to sparge for 3 min. The needles were removed, and the septum cap was wrapped with electrical tape to ensure sealing. The vial was fitted into the base of the photoreactor which was connected to a chiller with the temperature set to 25 °C. The reactions were irradiated at 454 nm at 100% intensity. The stirrers on each side of the plate were set at 1000 rpm to ensure efficient stirring at the corners and the center of the plate (Figure S3). After 24 h, the reaction was diluted with EtOAc, filtered through a plug of silica eluting with EtOAc, and concentrated under reduced pressure. The crude reaction mixture was dissolved in $CDCl_3$ (1.00 mL) and $CH_2Br_2$ (7.00 $\mu$L, 0.100 mmol) was added as an external standard. The yield of the desired product was analyzed using $^1$H NMR spectroscopy.

Additionally, for comparison a series of 10 Kessil reactions were conducted following the above procedure except that the vial was placed about 4 cm away from a 456 nm Kessil lamp and irradiated for 24 h while stirring at 800 rpm (Figure S15). A Kessil fan rig was used to prevent reactions from overheating. At the end of each reaction, the temperature of the reaction was recorded using a digital thermometer.

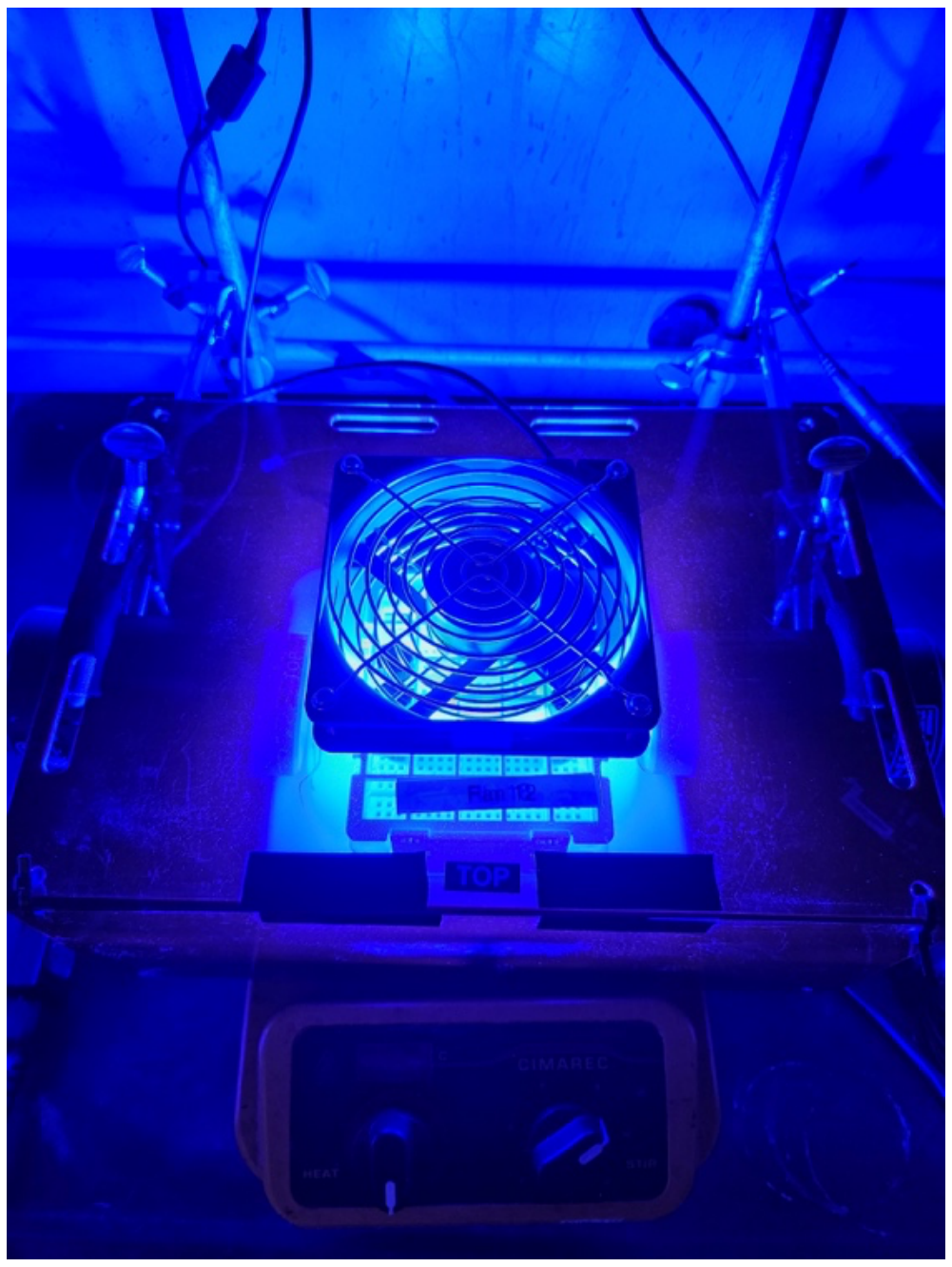

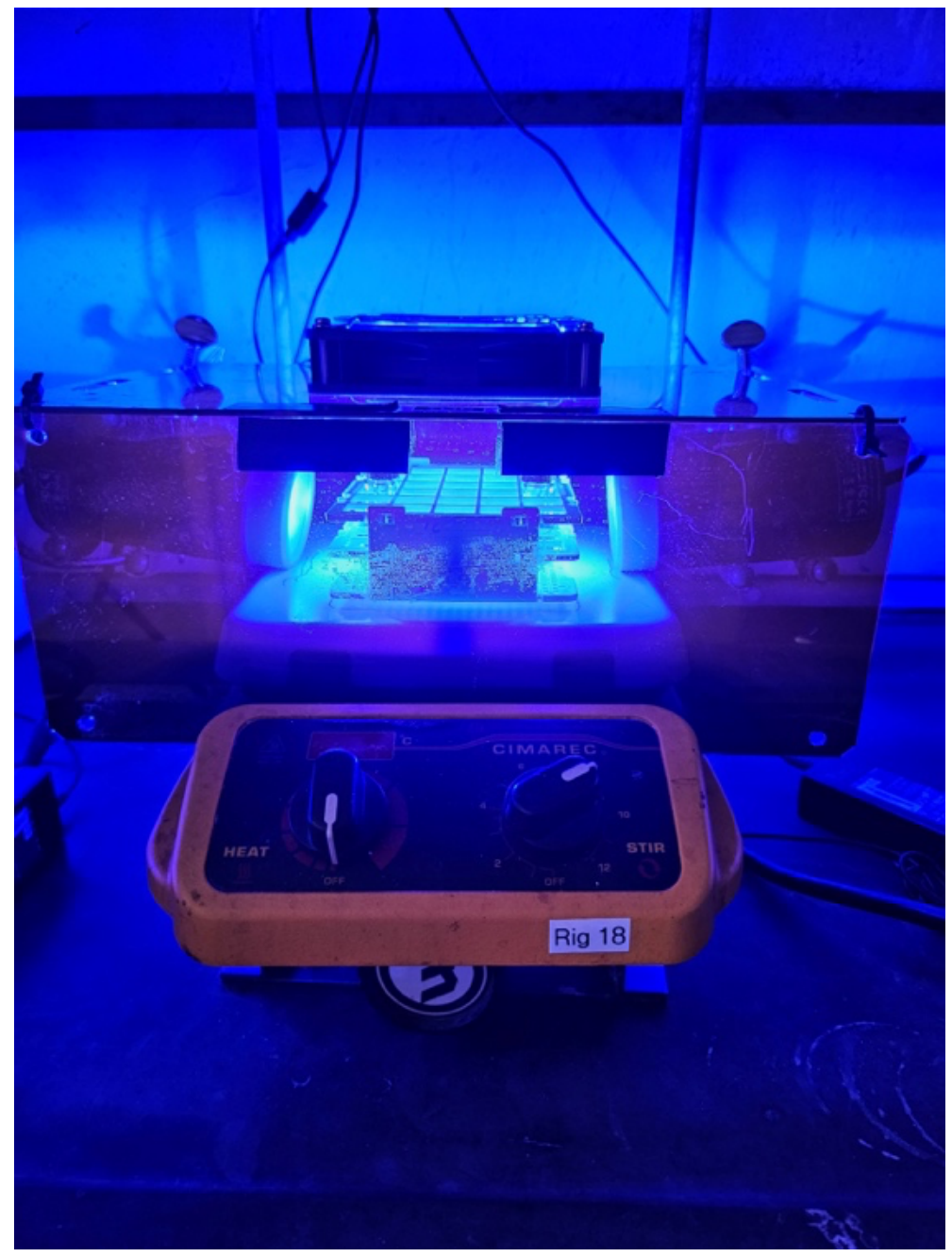

**Figure S15.** Kessil lamp (456 nm) rig setup for reaction screening.

**Table S4.** Reaction yields for the 10 individual Ce–catalyzed reactions using the 456 nm Kessil lamps.

| Reaction | Reaction yield (%) | Temperature (°C) |
|---|---|---|
| Reaction 1 | 91 | 27.5 |
| Reaction 2 | 85 | 27.1 |
| Reaction 3 | 86 | 26.1 |
| Reaction 4 | 81 | 26.4 |
| Reaction 5 | 75 | 26.0 |
| Reaction 6 | 74 | 27.0 |
| Reaction 7 | 82 | 26.7 |
| Reaction 8 | 87 | 26.5 |
| Reaction 9 | 84 | 26.8 |
| Reaction 10 | 90 | 27.2 |

Average yield ± std = 84% ± 6%
Average temperature ± std = 26.7 °C ± 0.5 °C
(n = 10)

### B5. Procedure for Cu–Mediated Dichlorination

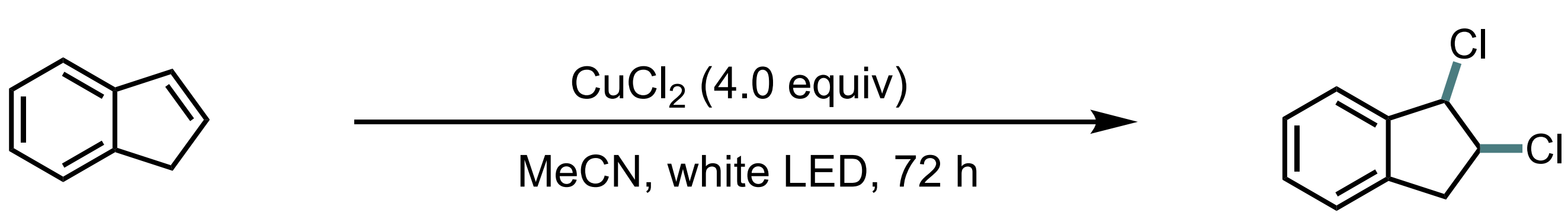

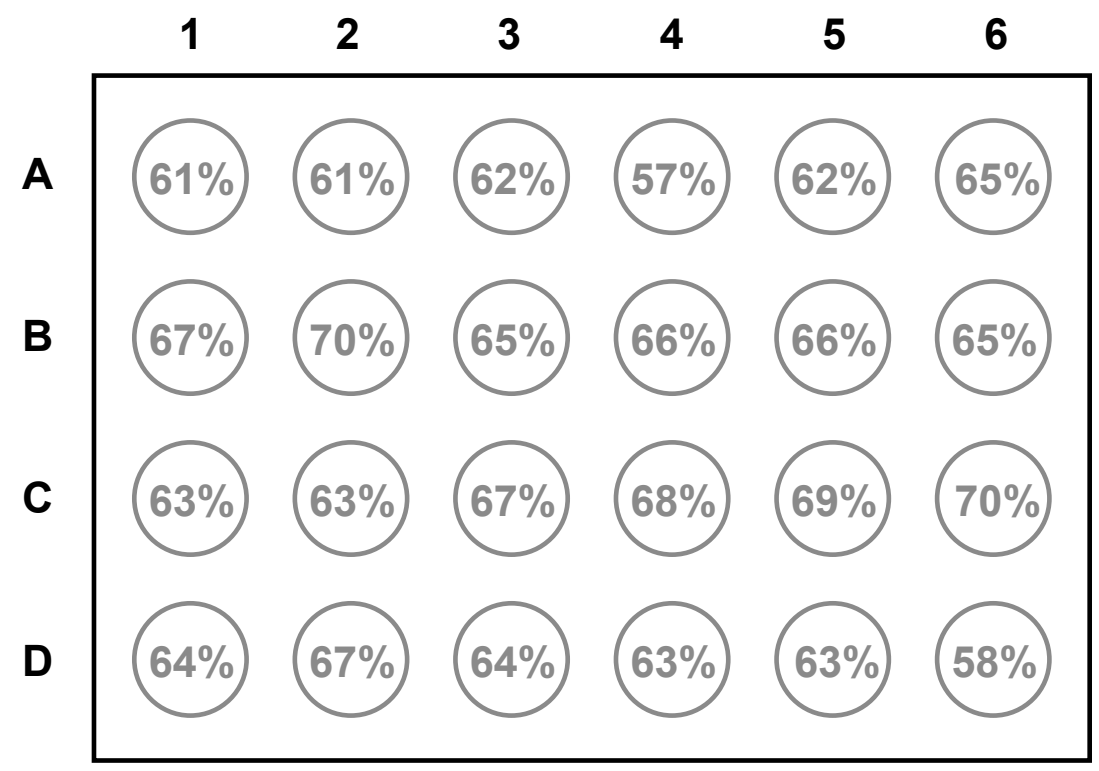


Average yield ± std = 64% ± 3%
(n = 24)

**Figure S16.** Summary of the results obtained using the tunable photoreactor for the Cu–mediated dichlorination of alkenes.

A series of 24 reactions was conducted concurrently. A representative reaction procedure for each reaction is communicated below:

To a 1-dram vial equipped with a PTFE-coated stir bar was added $CuCl_2$ (53.8 mg, 0.400 mmol, 4.00 equiv) and indene (11.7 $\mu$L, 0.100 mmol, 1.00 equiv) followed by 2.50 mL of MeCN. The vial was fitted with a septum cap, and a 21-gauge needle connected to a positive pressure of nitrogen was submerged in the solution along with another 21-gauge needle vent to allow the reaction to sparge for 3 min. The needles were removed, and the septum cap was wrapped with electrical tape to ensure sealing. The vial was fitted into the base of the photoreactor which was connected to a chiller with the temperature set to 25 °C. The reactions were irradiated with white light at 100% intensity. The stirrers on each side of the plate were set at 1000 rpm to ensure efficient stirring at the corners and the center of the plate (Figure S3). After 72 h, the reaction was diluted with Hexane/EtOAc (70:30), filtered through a plug of silica eluting with Hexane/EtOAc (70:30), and concentrated under reduced pressure. The crude reaction mixture was dissolved in $CDCl_3$ (1.00 mL) and $CH_2Br_2$ (3.50 $\mu$L, 0.0500 mmol) was added as an external standard. The yield of the desired product was analyzed using $^1H$ NMR spectroscopy.

Additionally, for comparison a series of 10 reactions were conducted following the above procedure except that the vial was irradiated using a CFL bulb and irradiated for 72 h while stirring at 800 rpm (Figure S17). A 10-inch table fan rig was used to prevent reactions from overheating. At the end of each reaction, the temperature of the reaction was recorded using a digital thermometer.

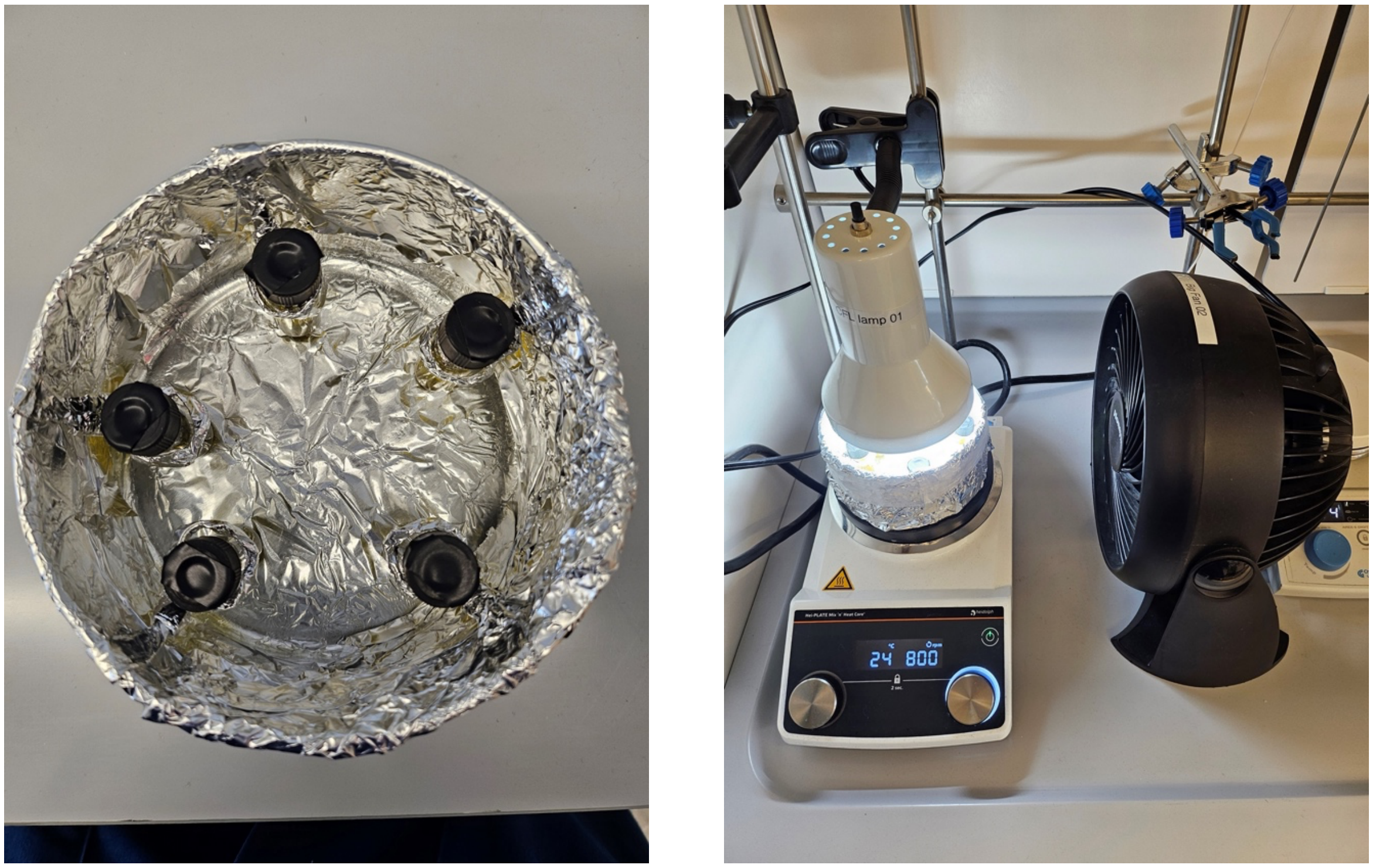


**Figure S17.** CFL bulb rig setup for reaction screening.

**Table S5.** Reaction yields for the 10 individual Cu–mediated reactions using the CFL bulb.

| Reaction | Reaction yield (%) | Temperature (°C) |
|---|---|---|
| Reaction 1 | 61 | 25.8 |
| Reaction 2 | 61 | 26.4 |
| Reaction 3 | 62 | 26.7 |
| Reaction 4 | 68 | 26.2 |
| Reaction 5 | 61 | 25.9 |
| Reaction 6 | 54 | 26.6 |
| Reaction 7 | 52 | 26.0 |
| Reaction 8 | 59 | 26.1 |
| Reaction 9 | 58 | 27.2 |
| Reaction 10 | 49 | 27.3 |

Average yield ± std = 59% ± 6%
Average temperature ± std = 26.4 °C ± 0.5 °C
(n = 10)

## B6. Procedure for Examining the Three Model Reaction in A Single Experiment Using the Tunable Photoreactor

A series of 24 reactions was conducted concurrently: 8 identical reactions for the Fe–catalyzed reaction (UV LED) were prepared according to the general procedure for Fe–photocatalyzed decarboxylative Giese addition using the tunable photoreactor (IV.B3), 8 identical reactions for

the Ce–catalyzed reaction (blue LED) were prepared according to the general procedure for Ce–catalyzed C–H amination (IV.B4), and 8 identical reactions for the Cu–mediated reaction (white LED) were prepared according to the general procedure for Cu–mediated dichlorination (IV.B5). The reactions were distributed randomly in the plate as shown in Figure S18. The reactions were irradiated at their respective wavelengths, and the LED panel was programmed to stop the LEDs at specific reaction times (Fe reactions: 24 h, Ce reactions: 24 h, and Cu reactions: 72 h). The stirrers on each side of the plate were set at 1000 rpm to ensure efficient stirring at the corners and the center of the plate (Figure S3). After irradiation, the reaction was diluted with EtOAc (for Fe and Ce reactions) or Hexane/EtOAc (70:30, for Cu reactions), filtered through a plug of silica eluting with EtOAc or Hexane/EtOAc (70:30), and concentrated under reduced pressure. The crude reaction mixture was dissolved in $CDCl_3$ (1.00 mL) and $CH_2Br_2$ (7.00 $\mu$L, 0.100 mmol for Ce reactions, or 3.50 $\mu$L, 0.0500 mmol for Fe and Cu reactions) was added as an external standard. The yield of the desired product was analyzed using $^1$H NMR spectroscopy.

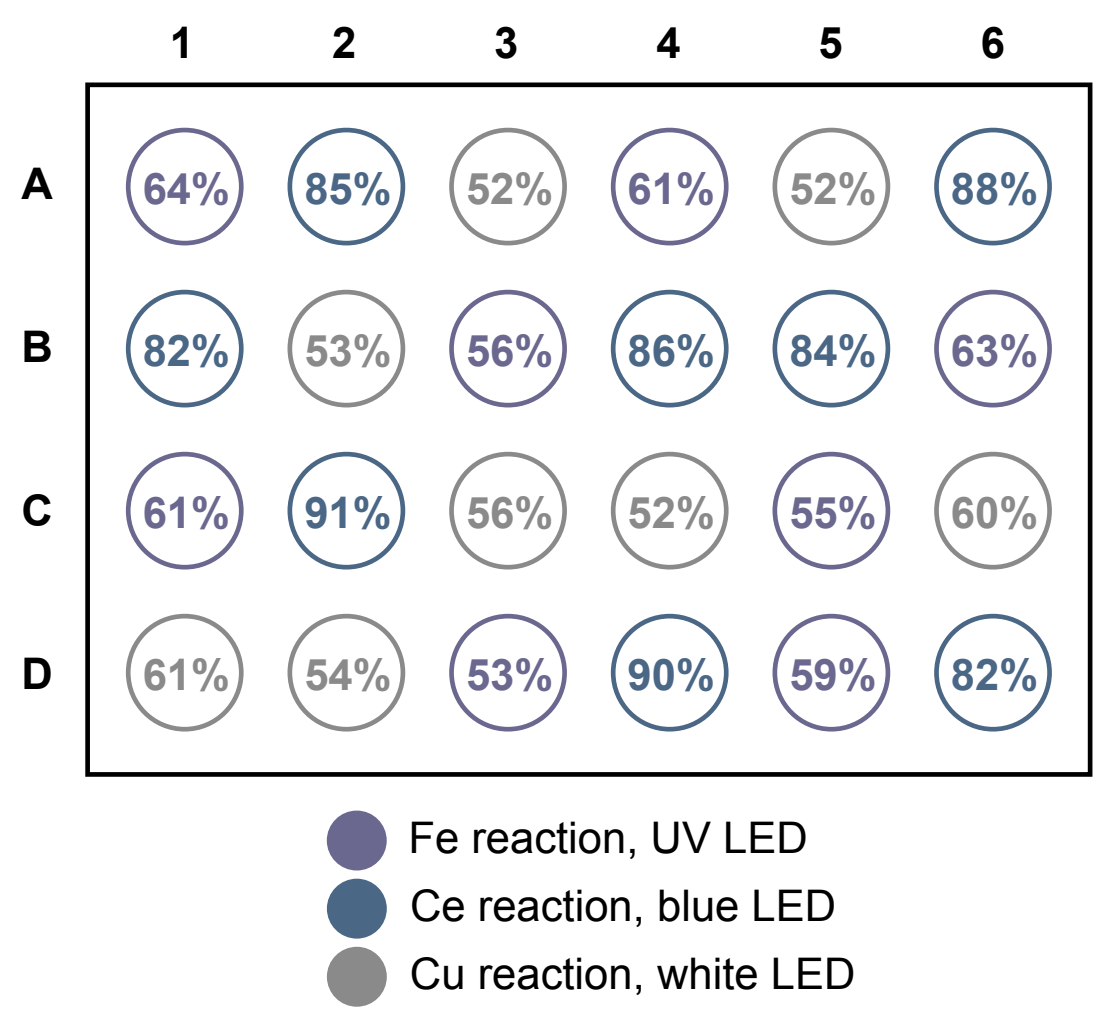


Fe reaction: Average yield ± std = 59% ± 4% (n = 8)
Ce reaction: Average yield ± std = 85% ± 3% (n = 8)
Cu reaction: Average yield ± std = 57% ± 3% (n = 8)

**Figure S18.** Plate design and summarized yields for examining Fe–catalyzed reaction (UV LED), Ce–catalyzed reaction (blue LED), and Cu–mediated reaction (white LED) in one single experiment.

## C. Reaction Progress

### C1. Procedure for Examining Reaction Progress of the Three Model Reactions

A series of 24 reactions was conducted concurrently: 7 identical reactions for the Fe–catalyzed reaction (UV LED) were prepared according to the general procedure for Fe–photocatalyzed decarboxylative Giese addition using the tunable photoreactor (IV.B3), 7 identical reactions for the Ce–catalyzed reaction (blue LED) were prepared according to the general procedure for Ce–catalyzed C–H amination (IV.B4), and 10 identical reactions for the Cu–mediated reaction (white LED) were prepared according to the general procedure for Cu–mediated dichlorination (IV.B5). The reactions were distributed in the plate as shown in Figure S19. The reactions were irradiated at their respective wavelengths. The LED panel was programmed to stop the LEDs at different time intervals varied as shown in Figure S19 and for each time point, the temperature

of the reaction was recorded using a digital thermometer. The stirrers on each side of the plate were set at 1000 rpm to ensure efficient stirring at the corners and the center of the plate (Figure S3). After irradiation, the reaction was diluted with EtOAc (for Fe and Ce reactions) or Hexane/EtOAc (70:30, for Cu reactions), filtered through a plug of silica eluting with EtOAc or Hexane/EtOAc (70:30), and concentrated under reduced pressure. The crude reaction mixture was dissolved in $CDCl_3$ (1.00 mL) and $CH_2Br_2$ (7.00 $\mu$L, 0.100 mmol for Ce reactions, or 3.50 $\mu$L, 0.0500 mmol for Fe and Cu reactions) was added as an external standard. The yield of the desired product was analyzed using $^1$H NMR spectroscopy.

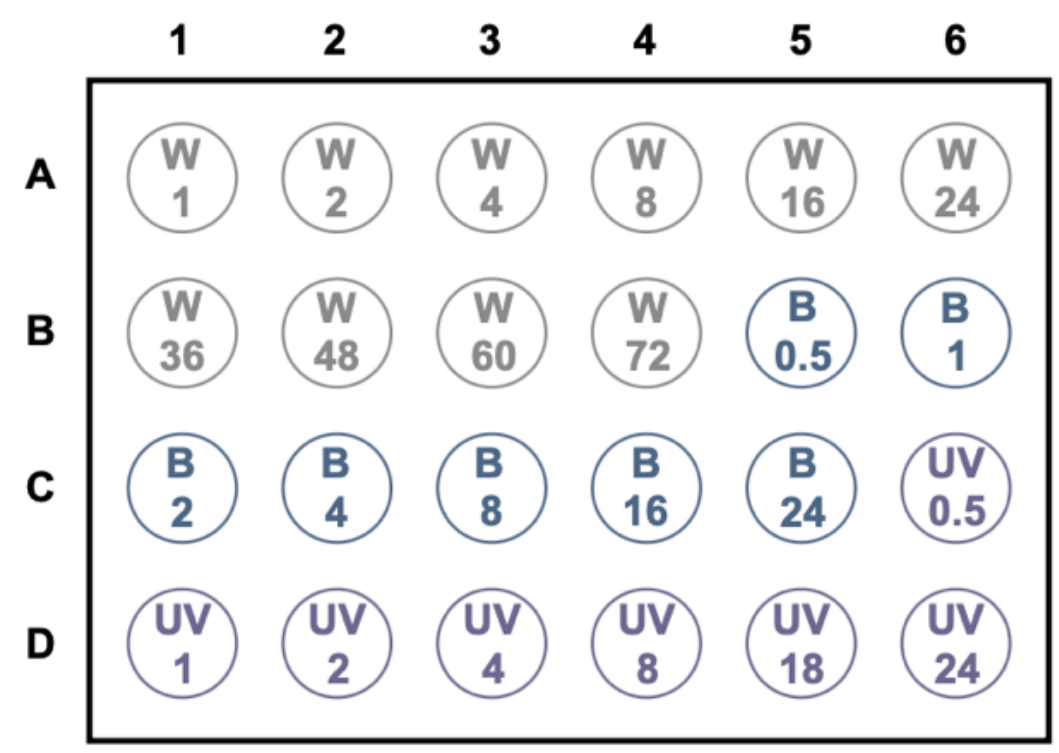


**Figure S19.** Plate design for examining reaction progress of Cu–mediated (W), Ce–catalyzed (B), and Fe–catalyzed (UV) reactions in one single experiment. The numbers represent the reaction time in hours.

**Table S6.** Summary of reaction yields and their corresponding reaction temperature upon irradiation for the reaction progress of Cu–mediated (white), Ce–catalyzed (blue), and Fe–catalyzed (UV) reactions in a single experiment.

| LED/Position | Time (h) | Reaction yield (%) | Temperature (°C) |
|---|---|---|---|
| White | 0 | 0 | 22.3 |
| White/A1 | 1 | 21 | 30.3 |
| White/A2 | 2 | 31 | 30.1 |
| White/A3 | 4 | 44 | 30.2 |
| White/A4 | 8 | 46 | 28.2 |
| White/A5 | 16 | 52 | 27.6 |
| White/A6 | 24 | 54 | 27.0 |
| White/B1 | 36 | 57 | 26.8 |
| White/B2 | 48 | 59 | 26.5 |
| White/B3 | 60 | 62 | 26.9 |
| White/B4 | 72 | 61 | 26.7 |
| Blue (454 nm) | 0 | 0 | 21.3 |
| Blue/B5 | 0.5 | 1 | 30.5 |
| Blue/B6 | 1 | 5 | 30.4 |
| Blue/C1 | 2 | 31 | 30.0 |
| Blue/C2 | 4 | 59 | 30.2 |
| Blue/C3 | 8 | 76 | 28.3 |
| Blue/C4 | 16 | 83 | 27.6 |
| Blue/C5 | 24 | 89 | 27.8 |

| | | | |
|---|---|---|---|
| UV (395 nm) | 0 | 0 | 22.1 |
| UV/C6 | 0.5 | 3 | 28.8 |
| UV/D1 | 1 | 8 | 28.9 |
| UV/D2 | 2 | 9 | 28.7 |
| UV/D3 | 4 | 19 | 28.4 |
| UV/D4 | 8 | 30 | 28.0 |
| UV/D5 | 16 | 55 | 27.3 |
| UV/D6 | 24 | 59 | 26.9 |

### C2. Procedure for Reaction Progress of Ce–Catalyzed C–H Amination at Different Light Intensity

A series of 24 reactions was conducted concurrently according to the general procedure for Ce–catalyzed C–H amination (IV.B4). The reactions were irradiated at 454 nm at 25–100% intensity (the intensity was varied across each row, see Figure S20). The LED panel was programmed to stop the LEDs at different time intervals varied across columns (1 h, 2 h, 4 h, 8 h, 16 h, and 24 h). The stirrers on each side of the plate were set at 1000 rpm to ensure efficient stirring at the corners and the center of the plate (Figure S3). At the end of each reaction, the temperature of the reaction was recorded using a digital thermometer. After irradiation, the reaction was diluted with EtOAc, filtered through a plug of silica eluting with EtOAc, and concentrated under reduced pressure. The crude reaction mixture was dissolved in $CDCl_3$ (1.00 mL) and $CH_2Br_2$ (7.00 $\mu$L, 0.100 mmol) was added as an external standard. The yield of the desired product was analyzed using $^1$H NMR spectroscopy.

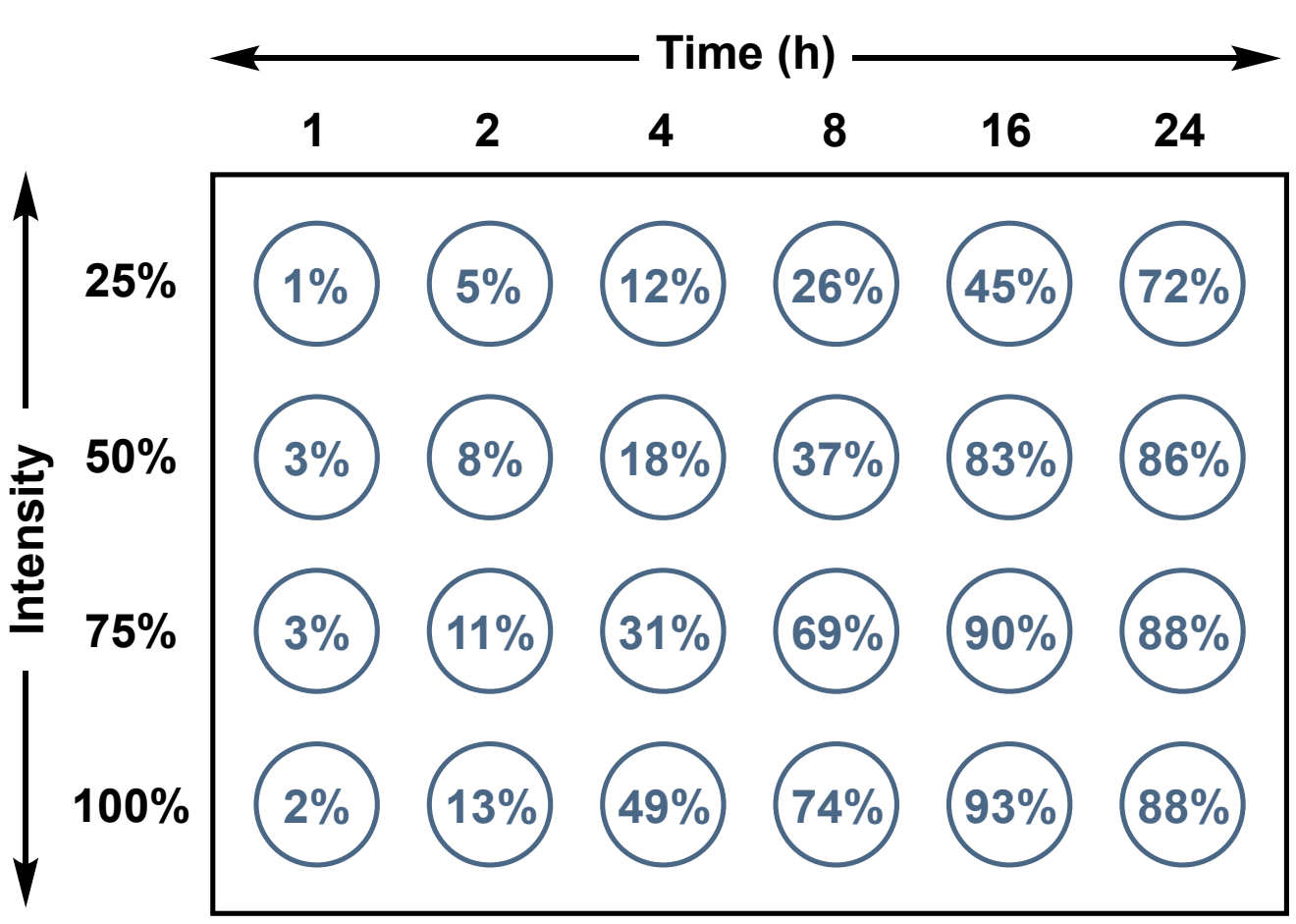


**Figure S20.** Plate design and summarized yields for examining reaction progress of Ce–catalyzed C–H amination at different light intensity.

**Table S7.** Summary of reaction yields and their corresponding reaction temperature upon irradiation for the reaction progress of Ce–catalyzed reactions under different intensities in one single experiment.

| LED/Position | Time (h) | Reaction yield (%) | Temperature (°C) |
|---|---|---|---|
| Blue (454 nm) | 0 | 0 | 22.2 |
| A1, 25% intensity | 1 | 1 | 28.2 |

| | | | |
|---|---|---|---|
| A2, 25% intensity | 2 | 5 | 27.5 |
| A3, 25% intensity | 4 | 12 | 27.6 |
| A4, 25% intensity | 8 | 26 | 27.0 |
| A5, 25% intensity | 16 | 45 | 26.6 |
| A6, 25% intensity | 24 | 72 | 26.5 |
| B1, 50% intensity | 1 | 3 | 28.9 |
| B2, 50% intensity | 2 | 8 | 28.0 |
| B3, 50% intensity | 4 | 18 | 28.1 |
| B4, 50% intensity | 8 | 37 | 27.9 |
| B5, 50% intensity | 16 | 83 | 26.7 |
| B6, 50% intensity | 24 | 86 | 26.8 |
| C1, 75% intensity | 1 | 3 | 29.1 |
| C2, 75% intensity | 2 | 11 | 28.7 |
| C3, 75% intensity | 4 | 31 | 28.7 |
| C4, 75% intensity | 8 | 69 | 28.2 |
| C5, 75% intensity | 16 | 90 | 26.7 |
| C6, 75% intensity | 24 | 88 | 27.0 |
| D1, 100% intensity | 1 | 2 | 29.0 |
| D2, 100% intensity | 2 | 13 | 28.9 |
| D3, 100% intensity | 4 | 49 | 28.9 |
| D4, 100% intensity | 8 | 74 | 28.3 |
| D5, 100% intensity | 16 | 93 | 27.1 |
| D6, 100% intensity | 24 | 88 | 26.9 |

## D. Procedure for Catalyst Selection for Fe–Catalyzed Derivatization of Gemfibrozil

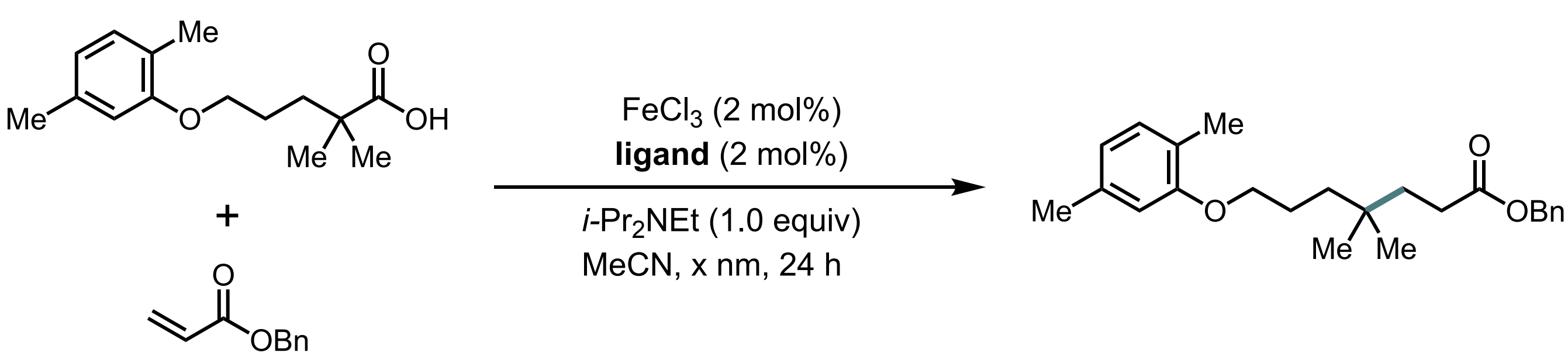


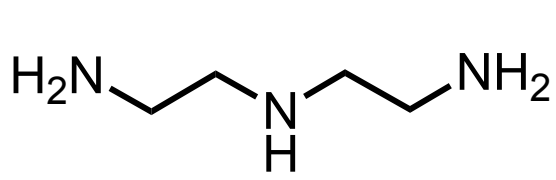


**L1, dien**

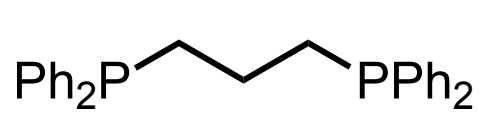


**L2, dppp**

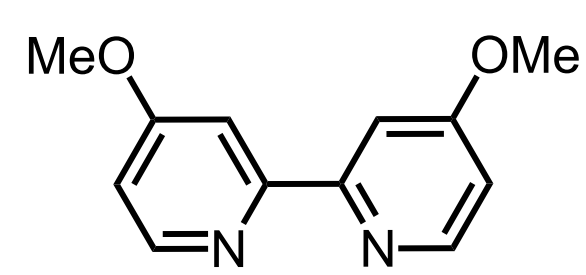


**L3, 4,4'-(OMe)$_2$bpy**

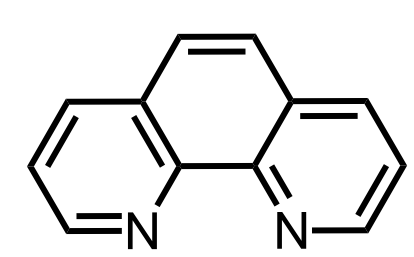


**L4, phen**

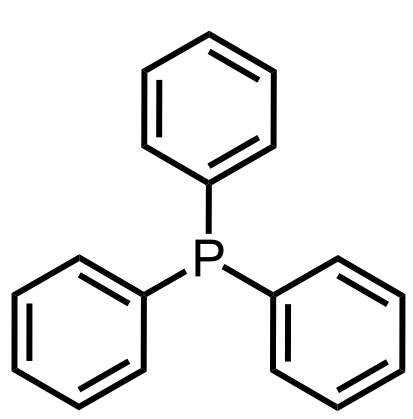


**L5, PPh$_3$**

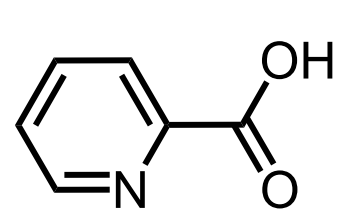


**L6, picolinic acid**

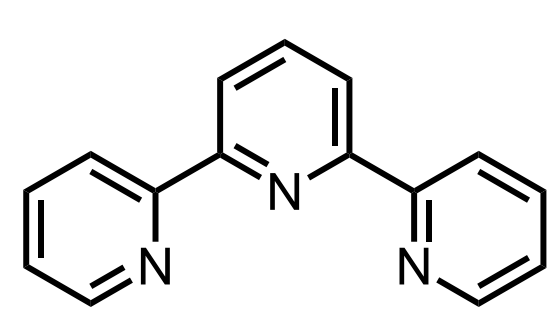


**L7, terpy**

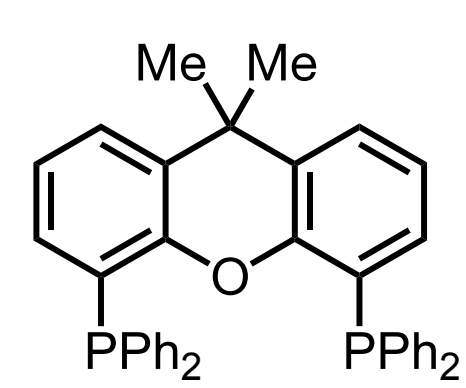


**L8, xantphos**

A series of 24 reactions was conducted concurrently: 8 different Fe-L combinations irradiated at 3 different wavelengths were prepared, with each set assigned to the same ligand. The reaction procedure for the plate is shown below:

A 1-dram vial equipped with a PTFE-coated stir bar was charged with dry acetonitrile (1.80 mL). To the reaction vial, Gemfibrozil (27.5 mg, 0.110 mmol, 1.10 equiv) was added, followed by the addition of $FeCl_3$ (0.00200 mmol, 0.0200 equiv) and ligand (0.00200 mmol, 0.0200 equiv), both as 0.0200 M stock solutions in dry acetonitrile. Subsequently, *N,N*-diisopropylethylamine (17.4 $\mu$L, 0.100 mmol, 1.00 equiv) and benzyl acrylate (15.4 $\mu$L, 0.100 mmol, 1.00 equiv) were added. The vial was fitted with a septum cap, and a 21-gauge needle connected to a positive pressure of nitrogen was submerged in the solution along with another 21-gauge needle vent to allow the reaction to sparge for 3 min. The needles were removed, and the septum cap was wrapped with electrical tape for sealing. The vial was fitted into the base of the photoreactor, which was connected to a chiller with the temperature set to 25 °C. Each Fe-L combination set of reactions was irradiated at UV, blue, and white wavelength at 100% intensity as shown in Figure S21. The stirrers on each side of the plate were set at 1000 rpm to ensure efficient stirring at the corners and the center of the plate (Figure S3). After 24 h, the reaction was diluted with EtOAc, filtered through a plug of silica eluting with EtOAc, and concentrated under reduced pressure. The crude reaction mixture was dissolved in $CDCl_3$ (1.00 mL) and $CH_2Br_2$ (3.50 $\mu$L, 0.0500 mmol) was added as an external standard. The yield of the desired product was analyzed using $^1$H NMR spectroscopy.

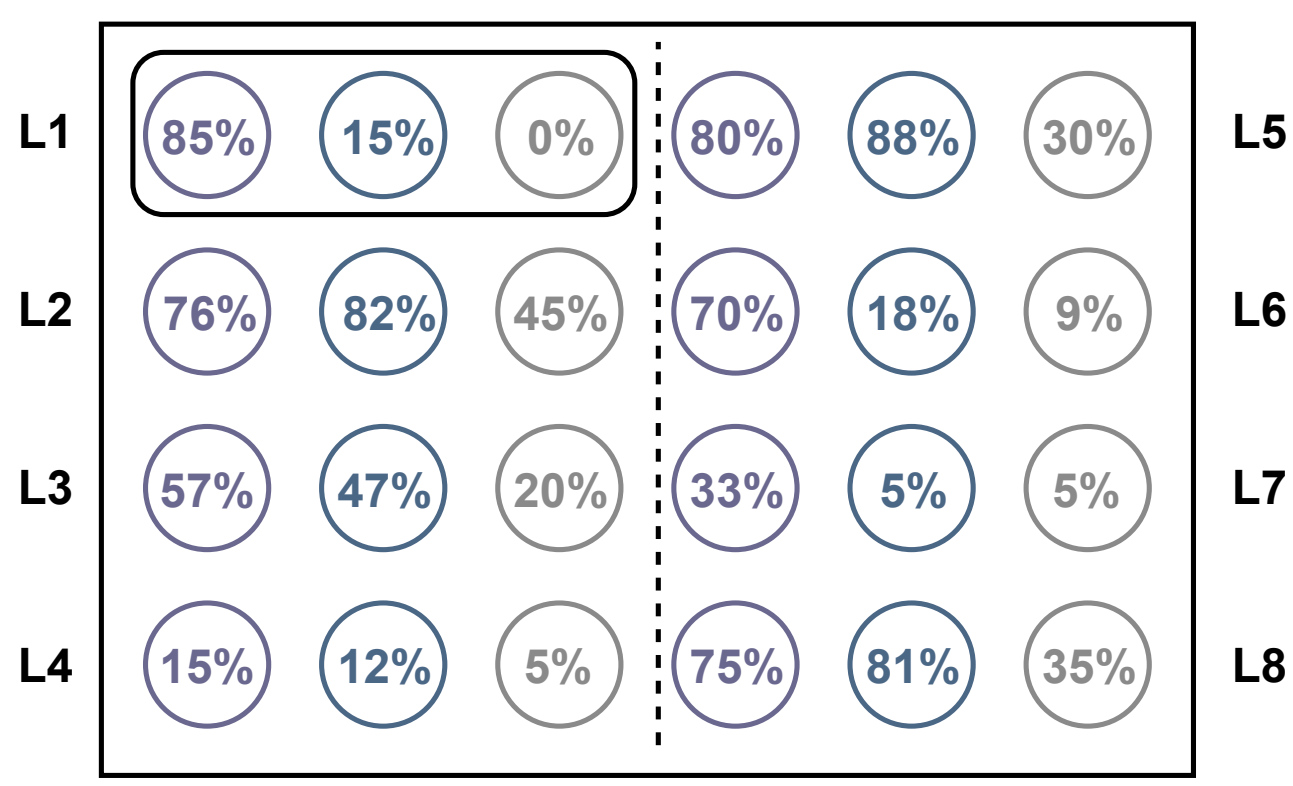


**Figure S21.** Plate design and summarized yields for catalyst selection for the Fe–catalyzed decarboxylative Giese addition of Gemfibrozil using 8 Fe-ligand combinations under UV, blue, and white irradiation. Reaction with L5 was conducted using a 1:1 ratio of Fe salt and ligand.

For L5, 1:1 and 1:2 ratios of Fe salt to ligand were examined and controls were run in the absence of the ligand following the same procedure described above (Table S8).

**Table S8.** Summary of reaction using different ratios of ligand L5.

| Ligand Conditions | UV LED (395 nm) | Blue LED (454 nm) | White LED |
|---|---|---|---|
| No ligand | 45% | 40% | 20% |
| Fe: L5 = 1:1 | 80% | 88% | 30% |
| Fe: L5 = 1:2 | 88% | 80% | 29% |

## V. UV–Vis Spectroscopy

UV–Vis spectroscopy was performed to support the ligation of the best performing ligands under the different wavelengths of irradiation when mixed with $FeCl_3$.

### A. $FeCl_3$ + dien (L1)

$FeCl_3$ stock solution: In a 1-dram vial, 1.62 mg of $FeCl_3$ (0.01 mmol) was added and diluted with 0.5 mL of MeCN. A 10.0 $\mu$L aliquot of the solution was taken from the mixture and diluted with MeCN to 2.00 mL in a quartz cuvette.

Dien stock solution: In a 1-dram vial, 1.10 $\mu$L dien (0.01 mmol) was added and diluted with 0.5 mL of MeCN. A 10.0 $\mu$L aliquot of the solution was taken from the mixture and diluted with MeCN to 2.00 mL in a quartz cuvette.

$FeCl_3$, dien stock solution: In a 1-dram vial, 0.1 mL of the $FeCl_3$ stock solution was combined with 0.1 mL of the dien stock solution. A 10.0 $\mu$L aliquot of the solution was taken from the mixture and diluted with MeCN to 2.00 mL in a quartz cuvette.

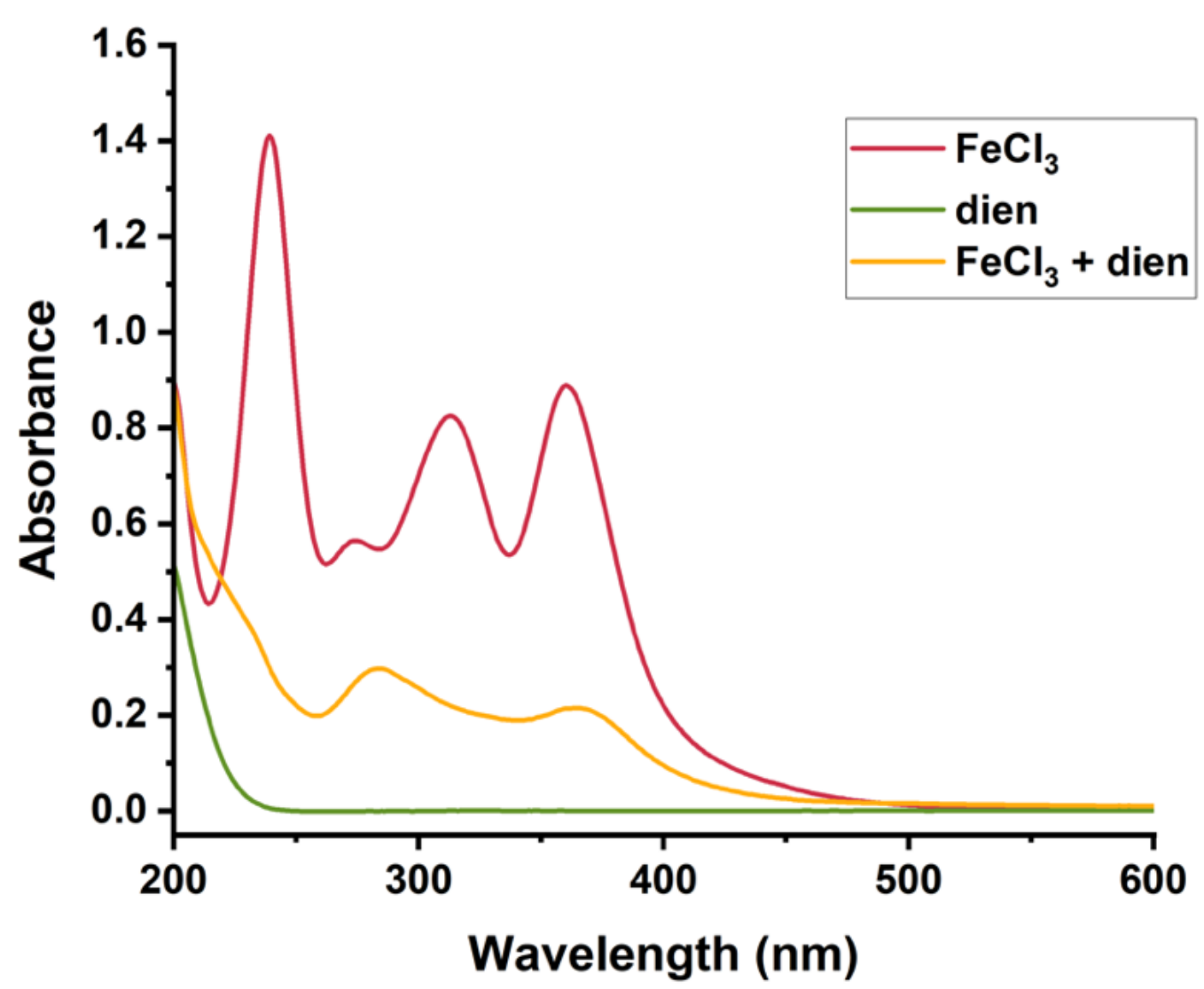


**Figure S22.** UV–Vis spectra for $FeCl_3$ with dien. UV–Vis spectra of $FeCl_3$ solution (red), dien solution (green), and $FeCl_3$ with dien solution (yellow).

**$FeCl_3$ + dppp (L2)**

$FeCl_3$ stock solution: In a 1-dram vial, 1.62 mg of $FeCl_3$ (0.01 mmol) was added and diluted with 0.5 mL of MeCN. A 10.0 $\mu$L aliquot of the solution was taken from the mixture and diluted with MeCN to 2.00 mL in a quartz cuvette.

Dppp stock solution: In a 1-dram vial, 4.12 mg of dppp (0.01 mmol) was added and diluted with 0.5 mL of MeCN. A 10.0 $\mu$L aliquot of the solution was taken from the mixture and diluted with MeCN to 2.00 mL in a quartz cuvette.

$FeCl_3$, dppp stock solution: In a 1-dram vial, 0.1 mL of the $FeCl_3$ stock solution was combined with 0.1 mL of the dien stock solution. A 10.0 $\mu$L aliquot of the solution was taken from the mixture and diluted with MeCN to 2.00 mL in a quartz cuvette.

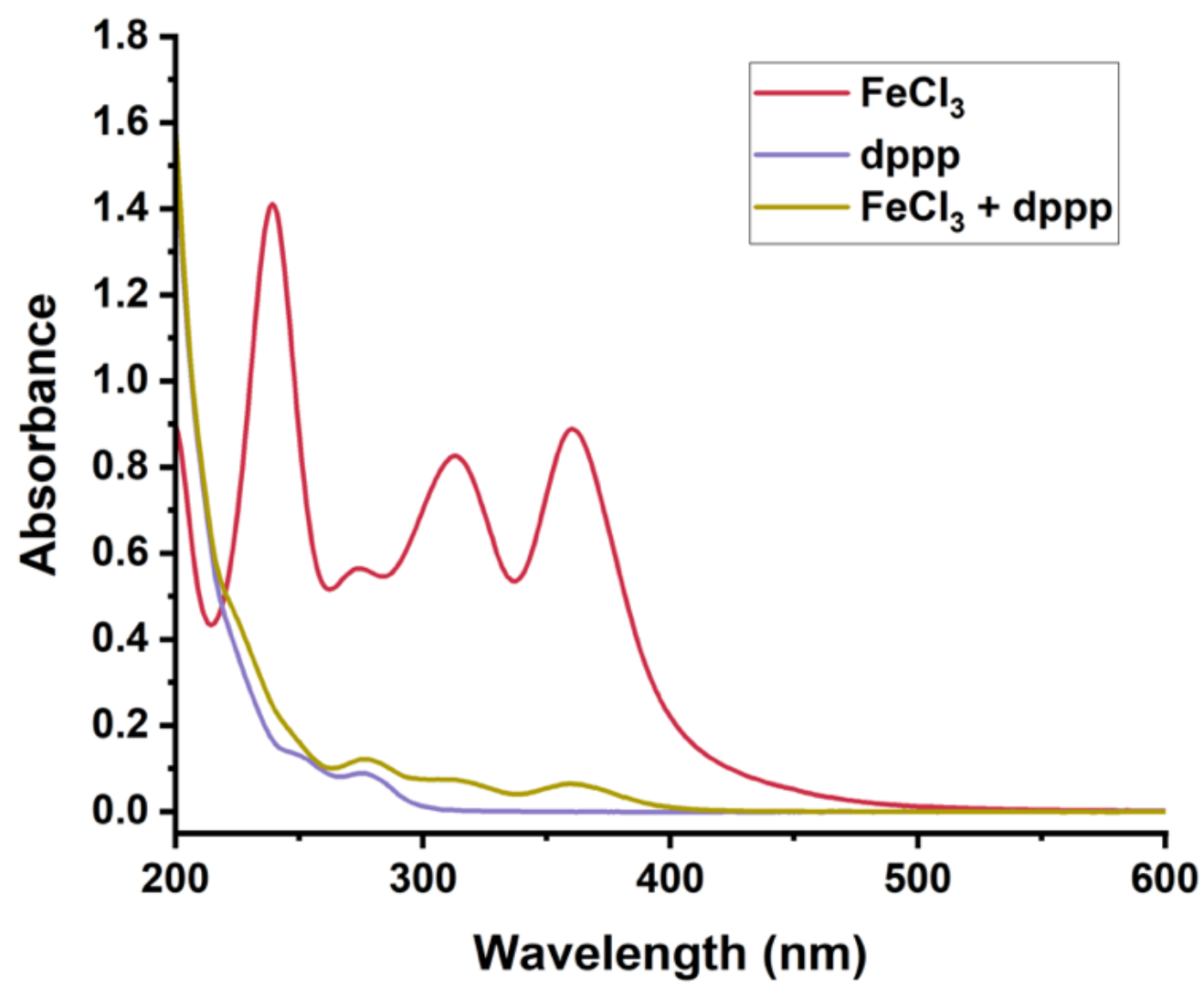


**Figure S23.** UV–Vis spectra for $FeCl_3$ with dppp. UV–Vis spectra of $FeCl_3$ solution (red), dppp solution (purple), and $FeCl_3$ with dppp solution (olive green).

**B. $FeCl_3$ + $PPh_3$ (L5)**

$FeCl_3$ stock solution: In a 1-dram vial, 1.62 mg of $FeCl_3$ (0.01 mmol) was added and diluted with 0.5 mL of MeCN. A 10.0 $\mu$L aliquot of the solution was taken from the mixture and diluted with MeCN to 2.00 mL in a quartz cuvette.

$PPh_3$ stock solution 1: In a 1-dram vial, 2.62 mg of $PPh_3$ (0.01 mmol) was added and diluted with 0.5 mL of MeCN. A 10.0 $\mu$L aliquot of the solution was taken from the mixture and diluted with MeCN to 2.00 mL in a quartz cuvette.

$PPh_3$ stock solution 2: In a 1-dram vial, 5.24 mg of $PPh_3$ (0.02 mmol) was added and diluted with 0.5 mL of MeCN. This solution was used to form a solution of Fe:$PPh_3$ = 1:2.

$FeCl_3$, $PPh_3$ (1:1) stock solution: In a 1-dram vial, 0.1 mL of the $FeCl_3$ stock solution was combined with 0.1 mL of the $PPh_3$ stock solution 1. A 10.0 $\mu$L aliquot of the solution was taken from the mixture and diluted with MeCN to 2.00 mL in a quartz cuvette.

$FeCl_3$, $PPh_3$ (1:2) stock solution: In a 1-dram vial, 0.1 mL of the $FeCl_3$ stock solution was combined with 0.1 mL of the $PPh_3$ stock solution 2. A 10.0 $\mu$L aliquot of the solution was taken from the mixture and diluted with MeCN to 2.00 mL in a quartz cuvette.

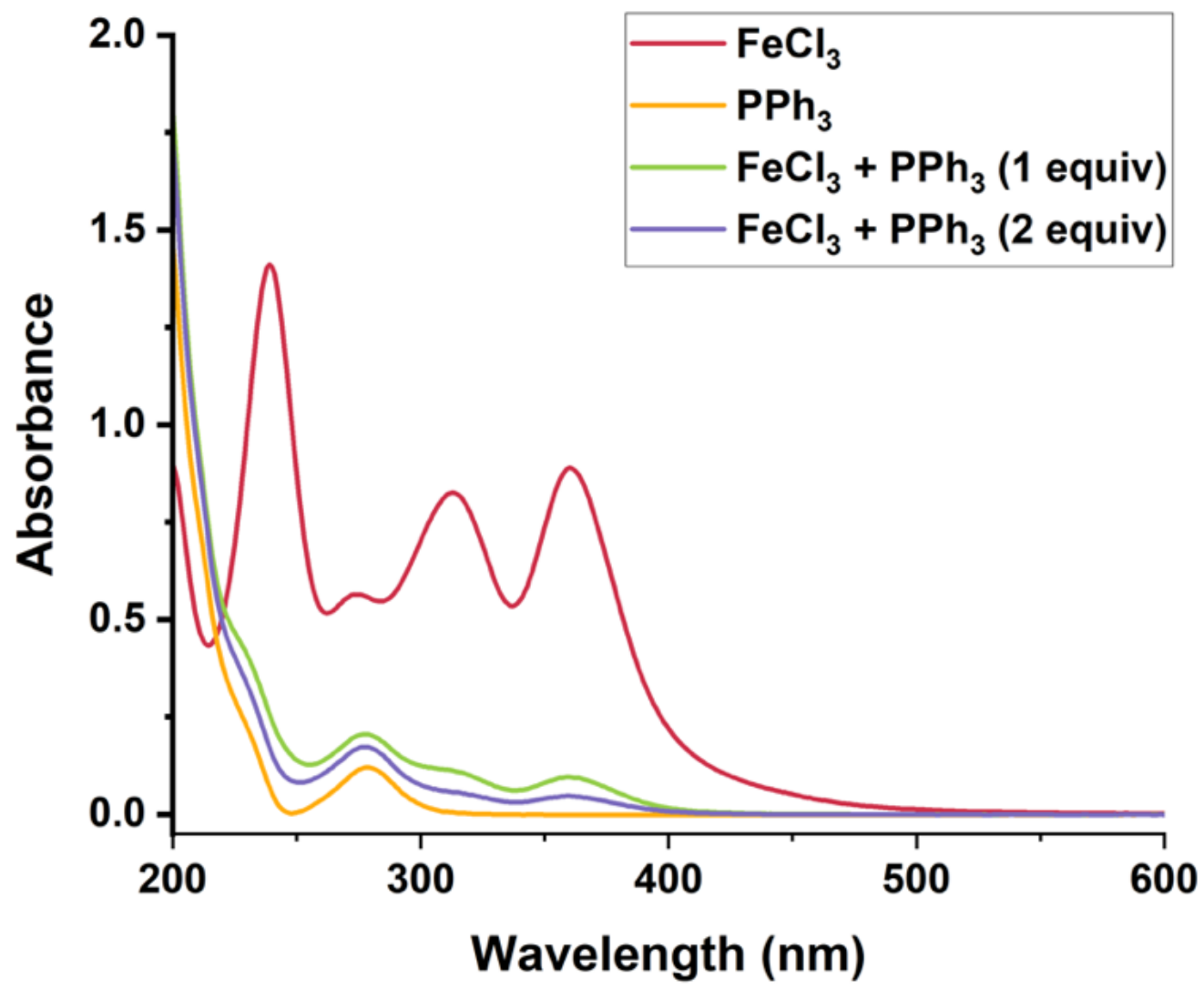


**Figure S24.** UV–Vis spectra for $FeCl_3$ with $PPh_3$. UV–Vis spectra of $FeCl_3$ solution (red), $PPh_3$ solution (orange), $FeCl_3$ with $PPh_3$ (1:1) solution (green), and $FeCl_3$ with $PPh_3$ (1:2) solution (purple).

In all cases new UV–Vis spectral signatures appeared with concomitant disappearance of $FeCl_3$ spectra after introducing ligand to $FeCl_3$, supporting ligation. While the UV–Vis spectra for isolated (dppp)$FeCl_3$ and $(PPh_3)_nFeCl_3$ (n=1-2) are not reported,[16,17] (dien)$FeCl_3$ has been reported by our group previously for which there is an X-ray crystal structure.[18,19]

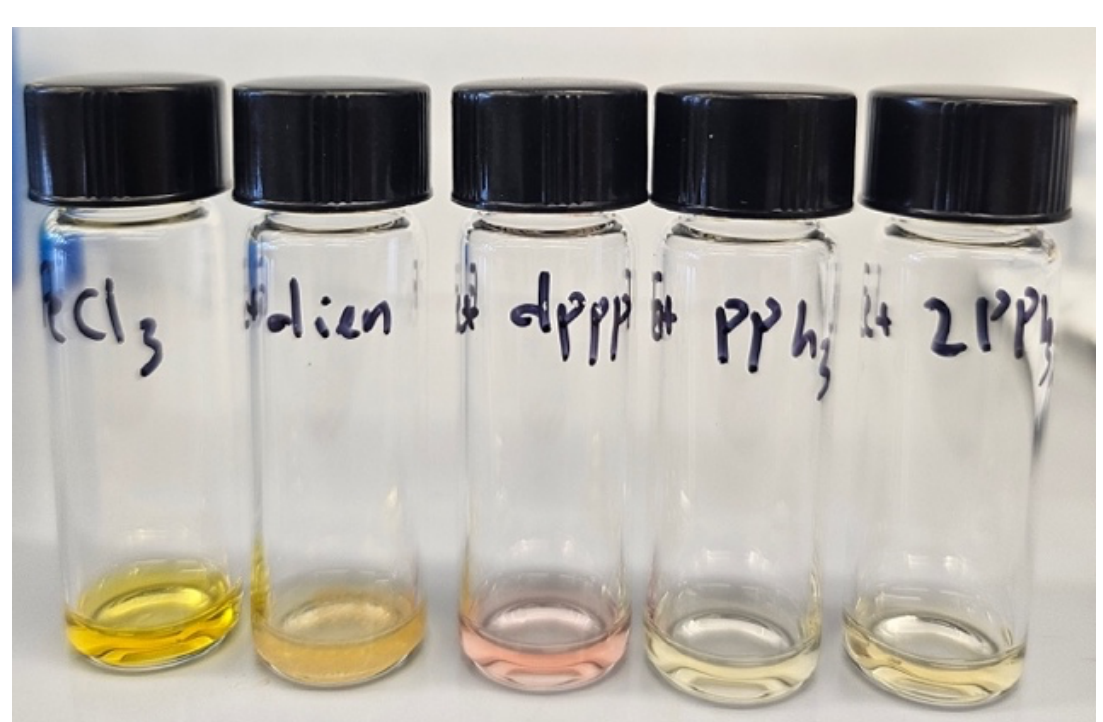

**Figure S25.** Image of the prepared stock solution. From left to right: $FeCl_3$ solution, $FeCl_3$ and dien solution, $FeCl_3$ and dppp solution, $FeCl_3$ and $PPh_3$ (1:1) solution, and $FeCl_3$ and $PPh_3$ (1:2) solution.

## VI. Compound Characterization

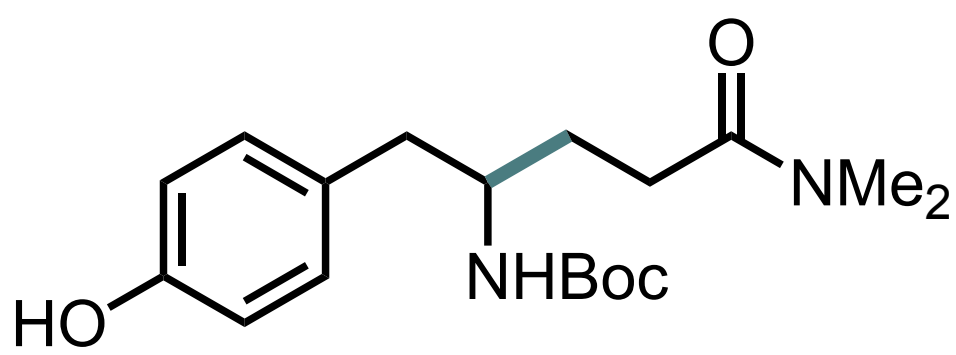

***tert*-butyl (5-(dimethylamino)-1-(4-hydroxyphenyl)-5-oxopentan-2-yl)carbamate**
Prepared according to the general procedure for Fe–photocatalyzed decarboxylative Giese addition using the tunable photoreactor (IV.B3) from Boc-Tyr-OH (30.9 mg, 0.110 mmol, 1.10 equiv) and *N,N*-dimethylacrylamide (10.3 $\mu$L, 0.100 mmol, 1.00 equiv) in MeCN (2.00 mL). The crude reaction was purified by flash column chromatography using EtOAc as the eluent to afford the mixture of titled compound coeluted with *N,N*-dimethylacrylamide. To this mixture, toluene (10 mL) was added, and reduced pressure was applied to remove *N,N*-dimethylacrylamide along with toluene and afford pure titled compound as a white solid in 61% yield (20.5 mg, 0.0610 mmol).

**$^{1}$H NMR (400 MHz, $CDCl_3$):** δ7.01 (d, $J$ = 8.0 Hz, 2H), 6.76–6.72 (m, 2H), 4.60 (d, $J$ = 9.2 Hz, 1H), 3.75 (s, 1H), 2.97 (s, 3H), 2.94 (s, 3H), 2.78 (dd, $J$ = 13.2, 4.8 Hz, 1H), 2.65 (dd, $J$ = 13.6, 7.2 Hz, 1H), 2.36 (t, $J$ = 7.6 Hz, 2H), 1.91–1.82 (m, 1H), 1.69–1.57 (m, 2H), 1.42 (s, 9H).

**$^{13}$C{$^{1}$H} NMR (101 MHz, $CDCl_3$):** δ 173.35, 156.05, 155.19, 130.68, 129.27, 115.45, 79.38, 52.19, 41.33, 37.45, 35.83, 30.31, 29.37, 28.56.

**HRMS-APCI+ (m/z): [M+H]$^+$** calculated for $C_{18}H_{29}N_2O_4^+$, 337.1596; found, 337.2122.

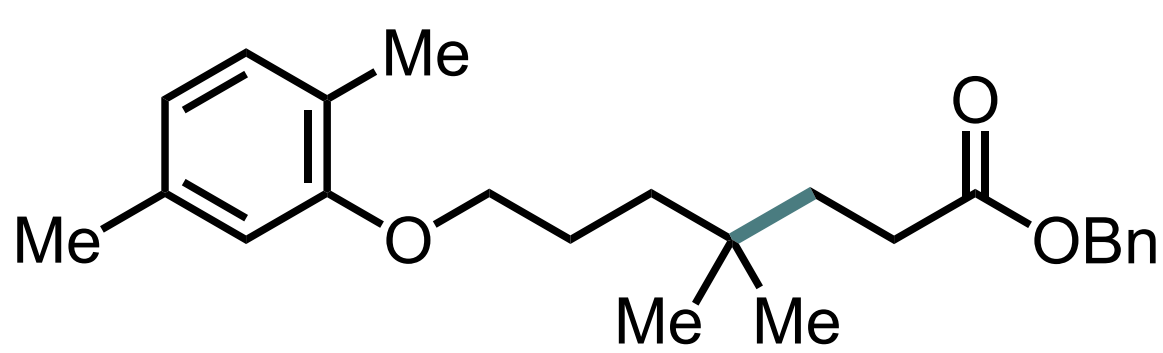

**Benzyl 7-(2,5-dimethylphenoxy)-4,4-dimethylheptanoate**
Prepared according to the general procedure for Fe–photocatalyzed decarboxylative Giese addition using the tunable photoreactor (IV.B3) from gemfibrozil (27.5 mg, 0.110 mmol, 1.10 equiv) and benzyl acrylate (15.4 $\mu$L, 0.100 mmol, 1.00 equiv) in MeCN (2.00 mL). The crude reaction was purified by flash column chromatography using EtOAc as the eluent to afford the titled compound as a colorless oil in 83% yield (29.5 mg, 0.0830 mmol). The NMR spectra were in accordance with those reported in the literature.[18]

**$^{1}$H NMR (400 MHz, $CDCl_3$):** δ 7.39–7.32 (m, 5H), 7.02 (d, $J$ = 7.6 Hz, 1H), 6.67 (*d*, J = 7.6 Hz, 1H), 6.63 (s, 1H), 5.13 (s, 2H), 3.92 (t, $J$ = 6.4 Hz, 2H), 2.38–2.32 (m, 5H), 2.19 (s, 3H), 1.80–1.72 (m, 2H), 1.66–1.62 (m, 2H), 1.40–1.36 (m, 2H), 0.92 (s, 6H).

**$^{13}$C{$^{1}$H} NMR (101 MHz, $CDCl_3$):** δ 174.3, 157.5, 136.6, 136.2, 130.4, 128.7, 128.4, 128.3, 123.7, 120.7, 112.0, 68.5, 66.4, 38.0, 36.4, 32.4, 29.7, 26.9, 24.3, 21.6, 15.9.

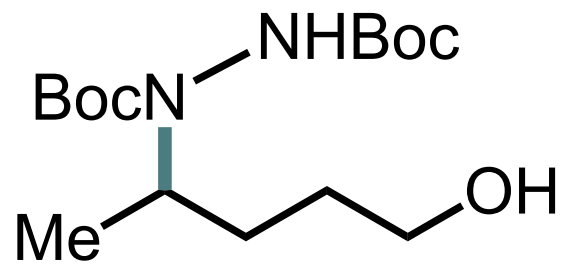


**Di-*tert*-butyl 1-(5-hydroxypentan-2-yl)hydrazine-1,2-dicarboxylate**
Prepared according to the general procedure for Ce–catalyzed C–H amination using 24-well tunable photoreactor (IV.B4) from di-*tert*-butyl azodicarboxylate (46.0 mg, 0.200 mmol, 1.00 equiv), and 1-pentanol (64.5 $\mu$L, 0.600 mmol, 3.00 equiv) in MeCN (2.00 mL). The crude reaction was purified by flash column chromatography using hexanes/acetone = 85:15 as the eluent to afford the titled compound as a colorless oil in 81% yield (51.5 mg, 0.162 mmol). The product gives two sets of NMR signals owing to the presence of rotamers around the amide. The NMR spectra were in accordance with those reported in the literature.[20]

**$^1$H NMR (400 MHz, CDCl$_3$):** δ 6.29–5.97 (m, 1H), 4.26–4.08 (m, 1H), 3.65–3.55 (m, 2H), 2.36–2.31 (m, 1H), 1.62–1.28 (m, 22H), 1.09 (d, *J* = 6.7 Hz, 3H).

**$^{13}$C{$^1$H} NMR (101 MHz, CDCl$_3$):** δ 156.4, 155.2, 81.2, 62.5, 54.4, 52.4, 52.2 (rotameric resonance), 30.4, 29.6, 29.2 (rotameric resonance), 28.4, 28.3, 18.4.

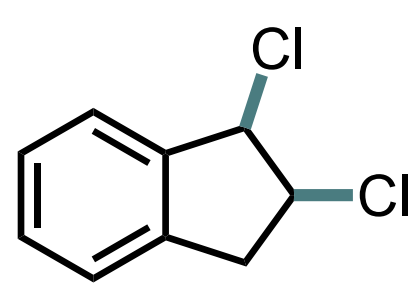


**1,2-dichloro-2,3-dihydro-1H-indene**
Prepared according to the general procedure for Cu–mediated dichlorination of alkene using 24-well tunable photoreactor (IV.B5) from CuCl$_2$ (53.8 mg, 0.400 mmol, 4.00 equiv) and indene (11.7 $\mu$L, 0.100 mmol, 1.00 equiv) in MeCN (2.50 mL). All 24 crude reaction mixtures were combined and purified by flash column chromatography using hexanes as the eluent to afford the titled compound in a 4:1 ratio inseparable colorless oil mixture with 1,2,3-trichloro-2,3-dihydro-1H-indene byproduct (308 mg of mixture, 60% yield with respect to the titled compound). The NMR spectra were in accordance with those reported in the literature[21] and are reported as a mixture with 1,2,3-trichloro-2,3-dihydro-1H-indene.

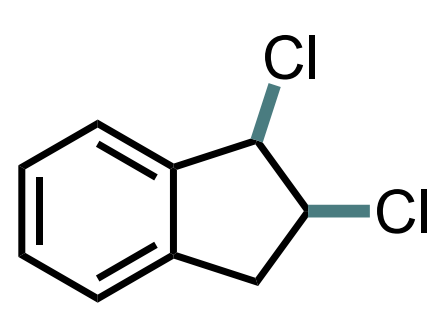


**$^1$H NMR (500 MHz, CDCl$_3$):** δ 7.47–7.28 (m, 4H), 5.35 (d, *J* = 2.5 Hz, 1H), 4.66 (dt, *J* = 6.5, 3.5 Hz, 1H), 3.71 (dd, *J* = 16.5, 6.0 Hz, 1H), 3.19 (dd, *J* = 16.5, 3.5 Hz, 1H).

**$^{13}$C{$^1$H} NMR (101 MHz, CDCl$_3$):** δ 139.94, 139.92, 129.8, 128.0, 125.6, 125.2, 67.7, 64.6, 40.8.

**HRMS-EI** (m/z): $[M]^+$ calculated for $C_9H_8Cl_2$ 186.0004; found 186.0008.

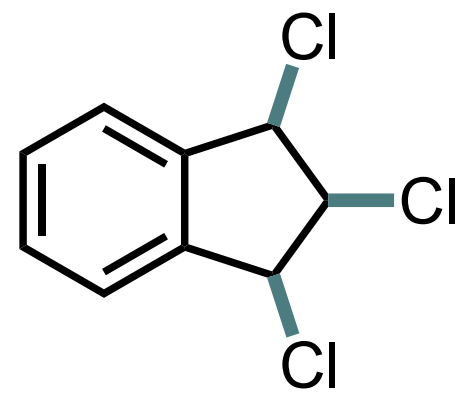


**1,2,3-trichloro-2,3-dihydro-1H-indene**

**$^1$H NMR (500 MHz, $CDCl_3$):** δ 7.47–7.28 (m, 4H), 5.27 (d, *J* = 5.5 Hz, 2H), 4.59 (t, *J* = 5.5 Hz, 1H).

**$^{13}C\{^1H\}$ NMR (101 MHz, $CDCl_3$):** δ 138.8, 130.5, 125.2, 71.7, 64.4.

**HRMS-EI** (m/z): $[M]^+$ calculated for $C_9H_7Cl_3$ 219.9611; found 219.9608.

# VII. NMR Spectra

## $^{1}$H NMR Spectrum of *tert*-butyl (5-(dimethylamino)-1-(4-hydroxyphenyl)-5-oxopentan-2-yl)carbamate (400 MHz, $CDCl_3$)

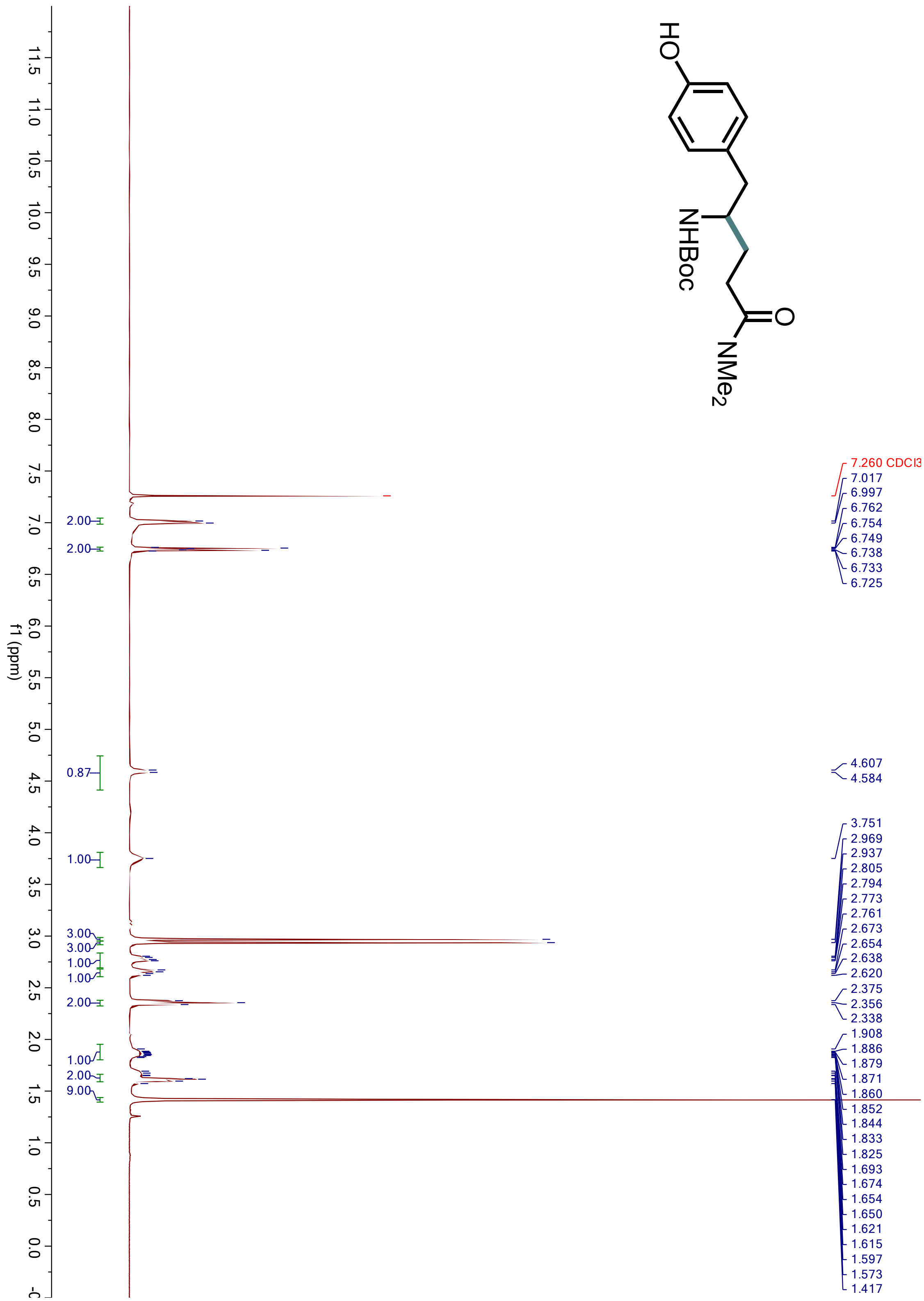

**$^{13}C\{^1H\}$ NMR Spectrum of *tert*-butyl (5-(dimethylamino)-1-(4-hydroxyphenyl)-5-oxopentan-2-yl)carbamate (101 MHz, $CDCl_3$)**

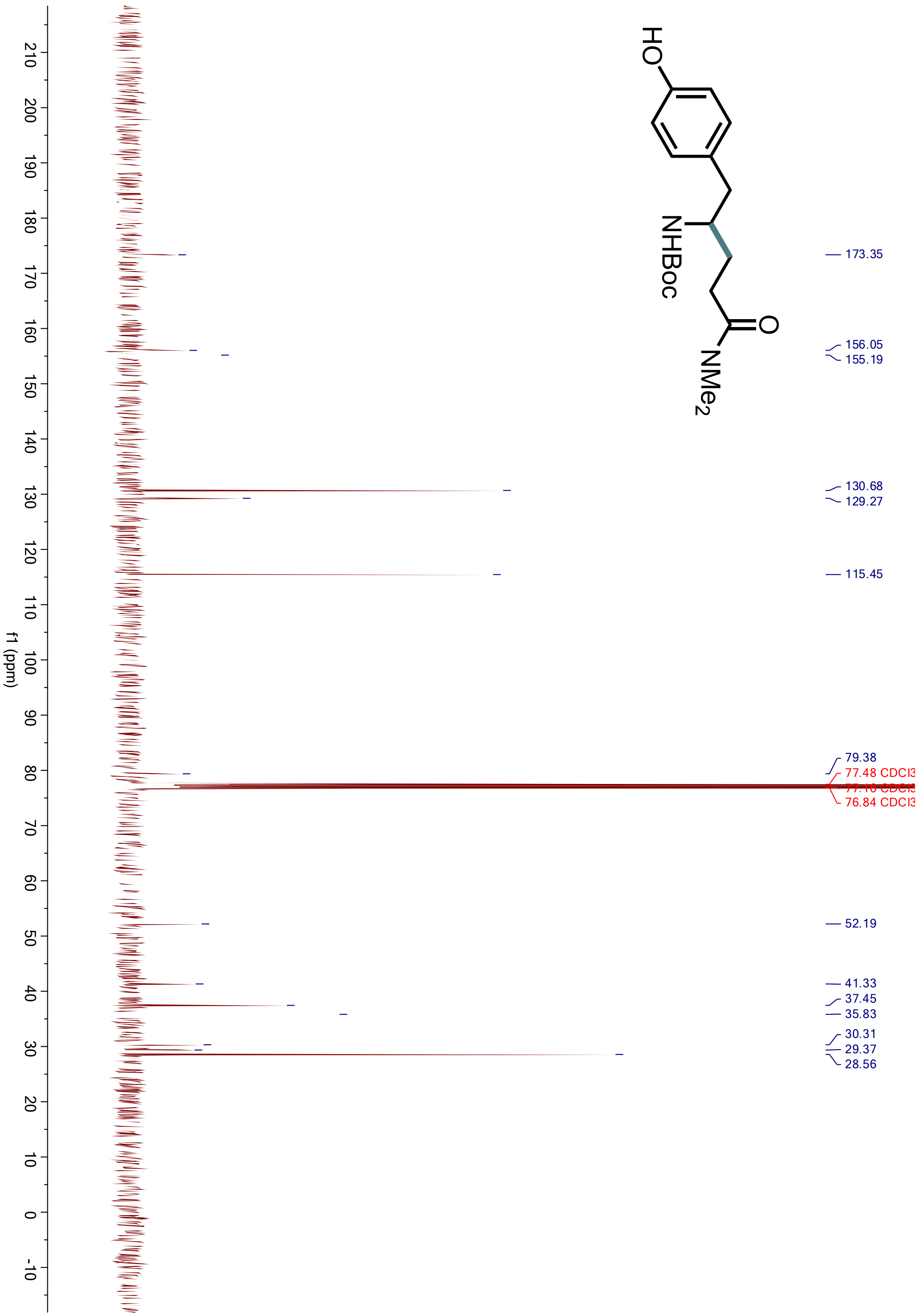

**$^1$H NMR Spectrum of Benzyl 7-(2,5-dimethylphenoxy)-4,4-dimethylheptanoate (400 MHz, $CDCl_3$)**

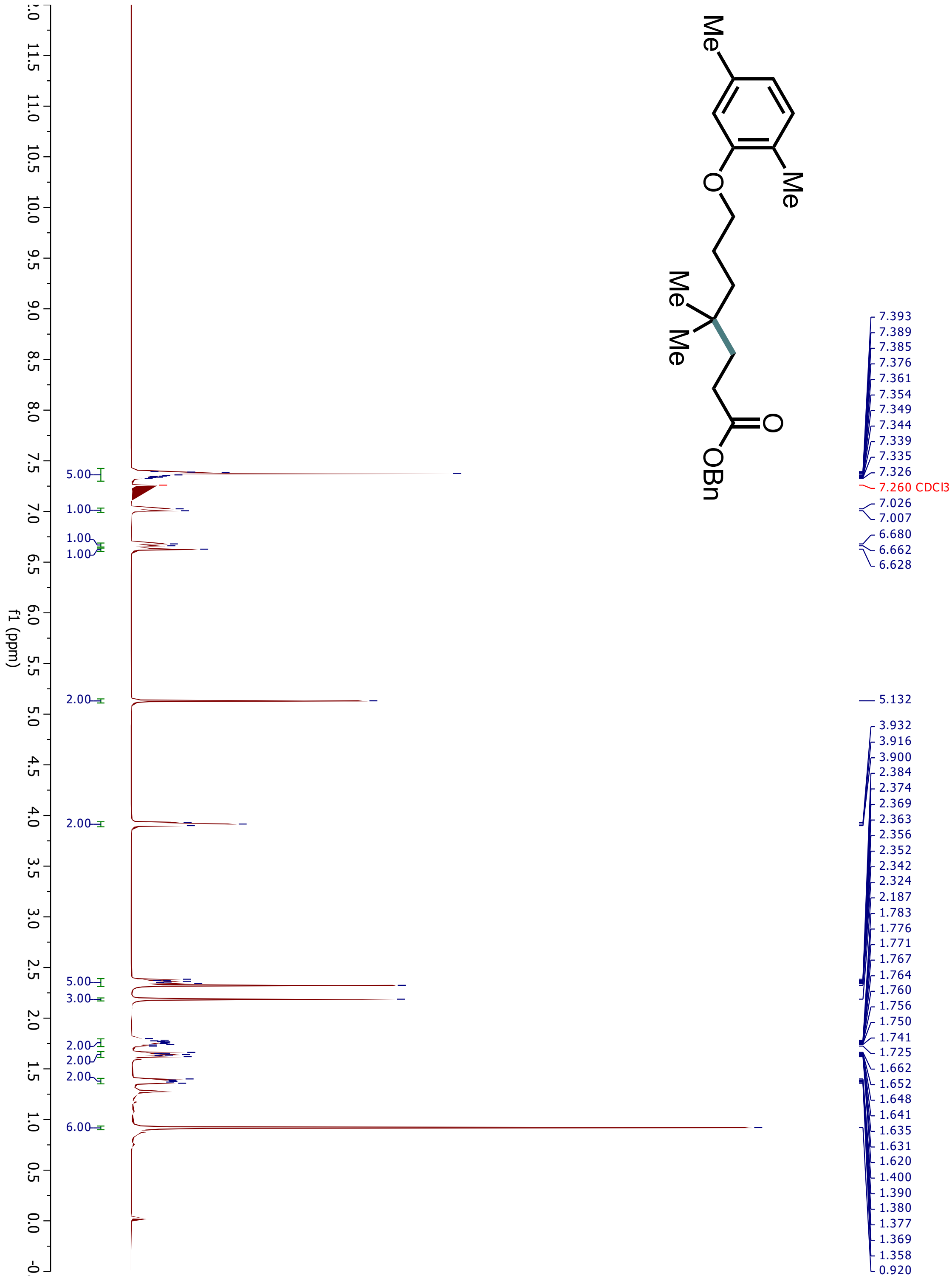

**$^{13}$C NMR Spectrum of Benzyl 7-(2,5-dimethylphenoxy)-4,4-dimethylheptanoate (101 MHz, $CDCl_3$)**

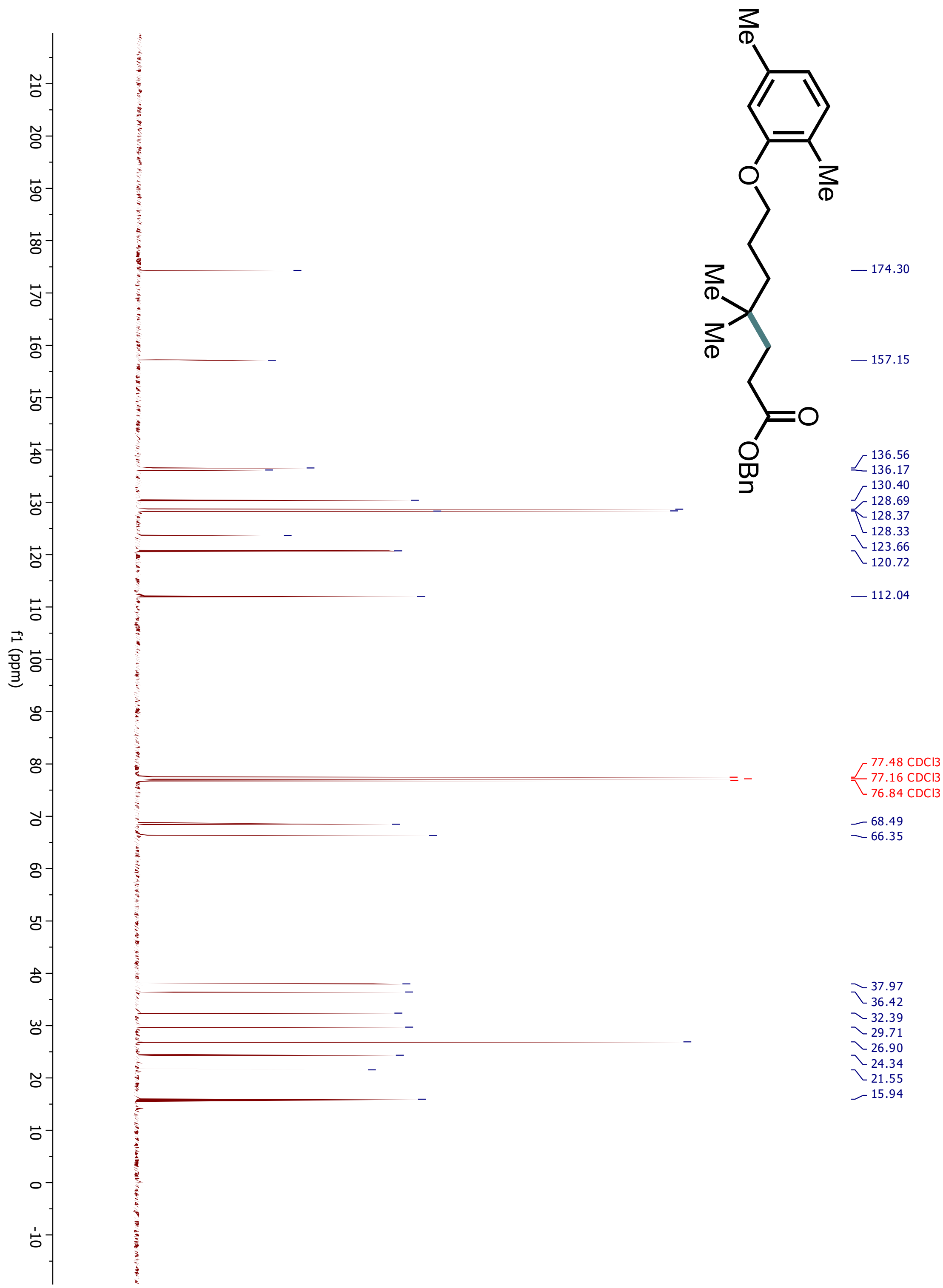

**$^{1}$H NMR Spectrum of *Di-tert-butyl 1-(5-hydroxypentan-2-yl)hydrazine-1,2-dicarboxylate***
**(400 MHz, $CDCl_3$)**

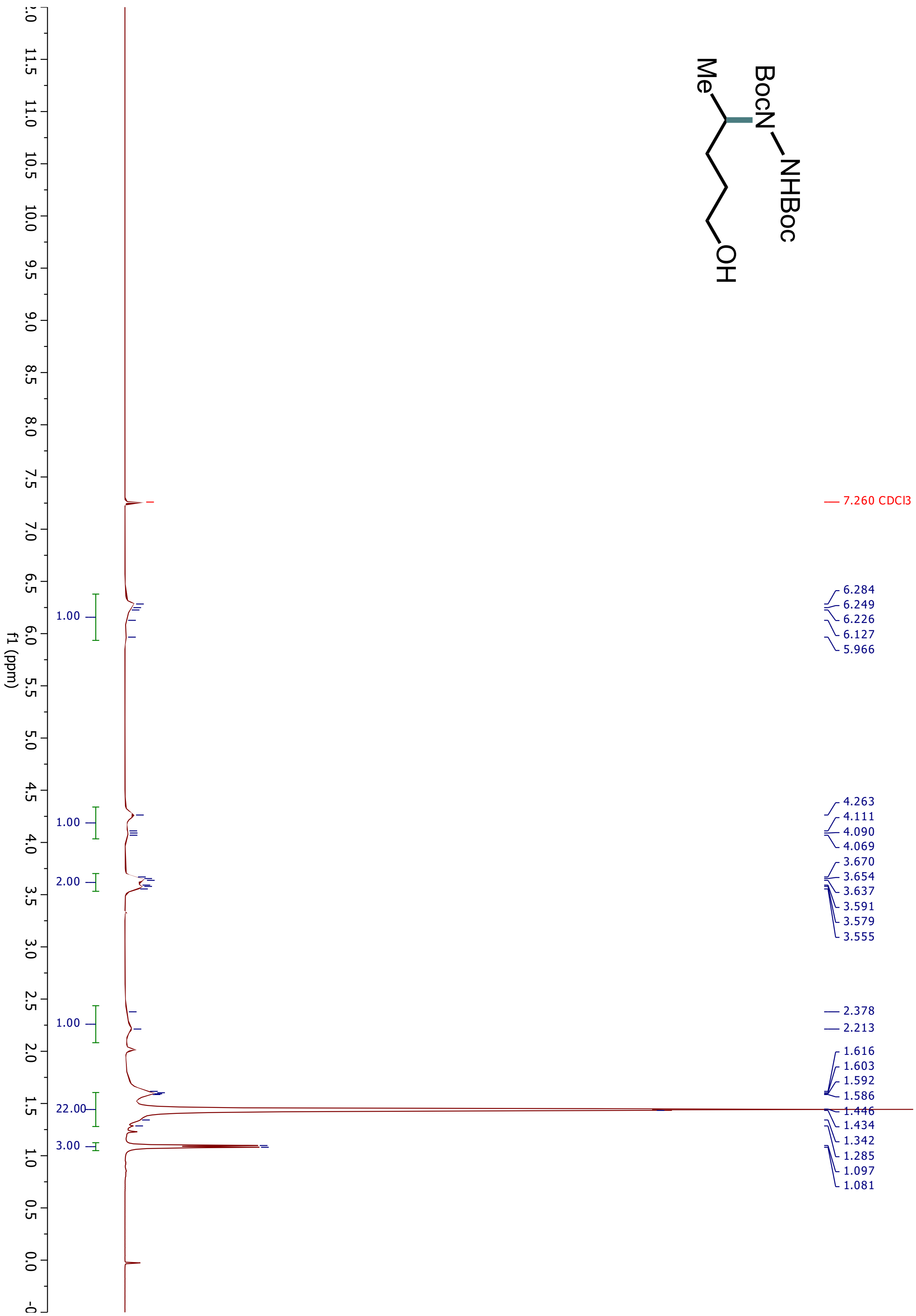

**$^{13}C\{^1H\}$ NMR Spectrum of *Di-tert-butyl 1-(5-hydroxypentan-2-yl)hydrazine-1,2-dicarboxylate* (101 MHz, $CDCl_3$)**

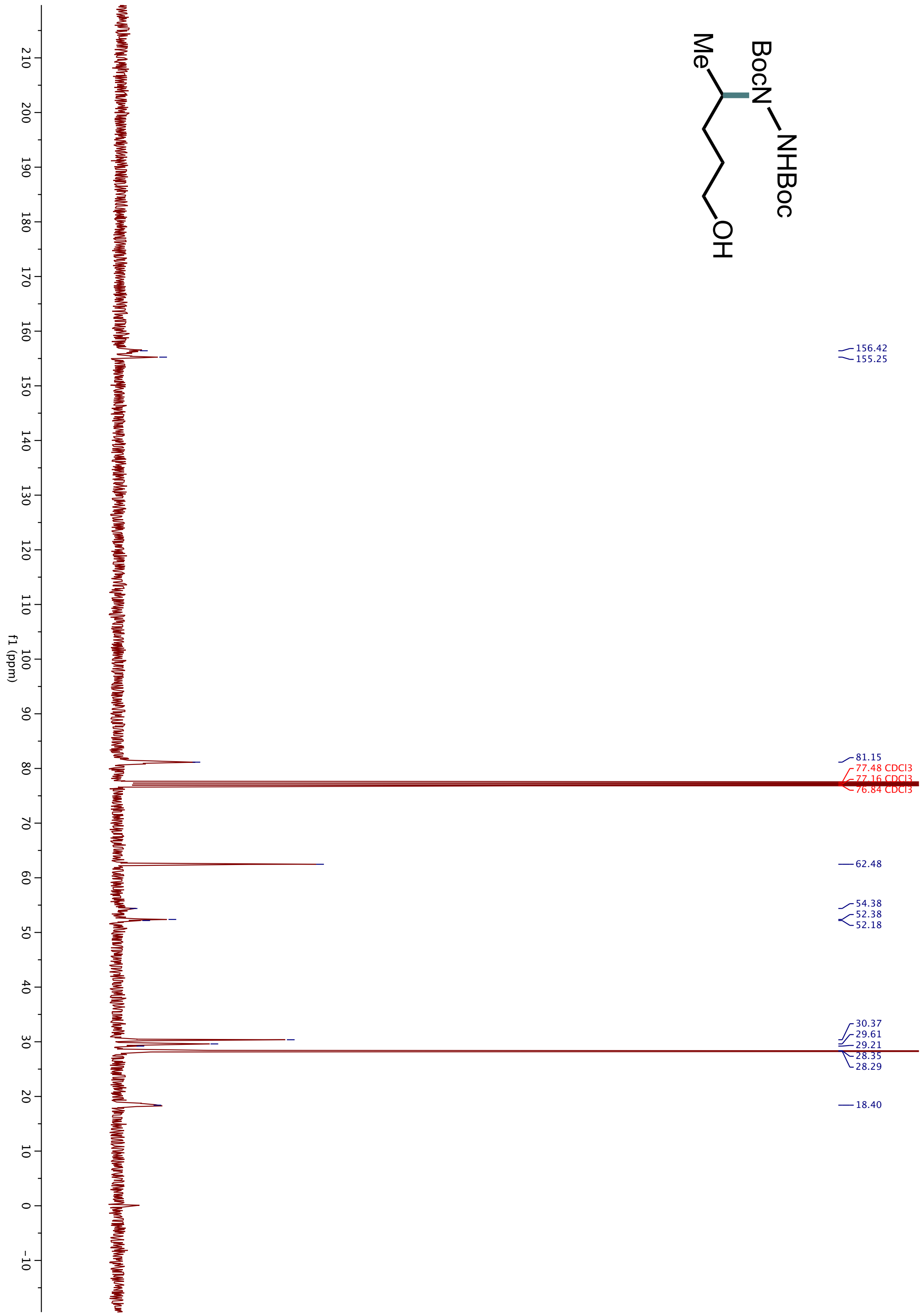

## $^1$H NMR Spectrum of *1,2-dichloro-2,3-dihydro-1H-indene* (500 MHz, $CDCl_3$)

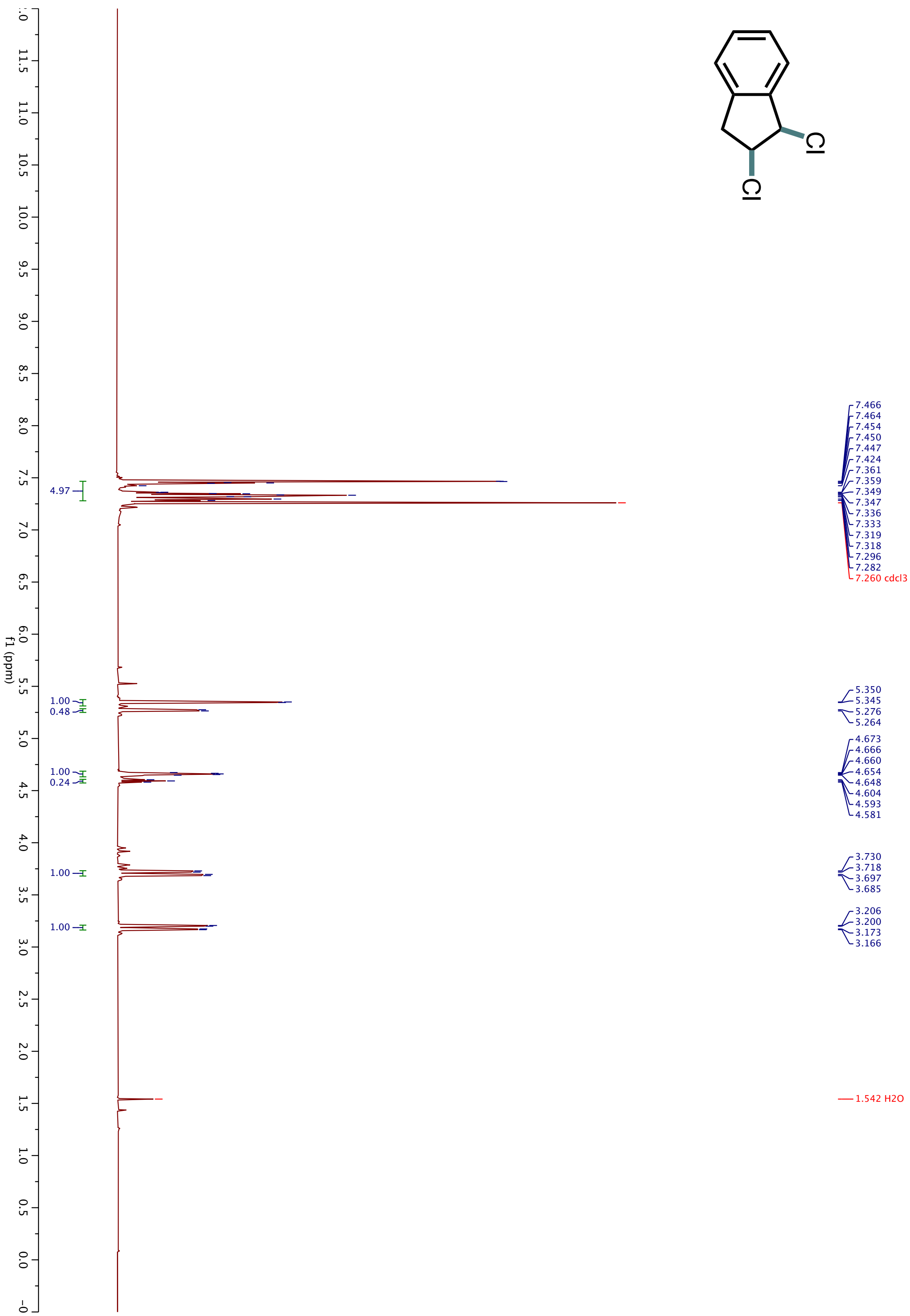

**$^{13}$C NMR Spectrum of *1,2-dichloro-2,3-dihydro-1H-indene* (101 MHz, $CDCl_3$)**

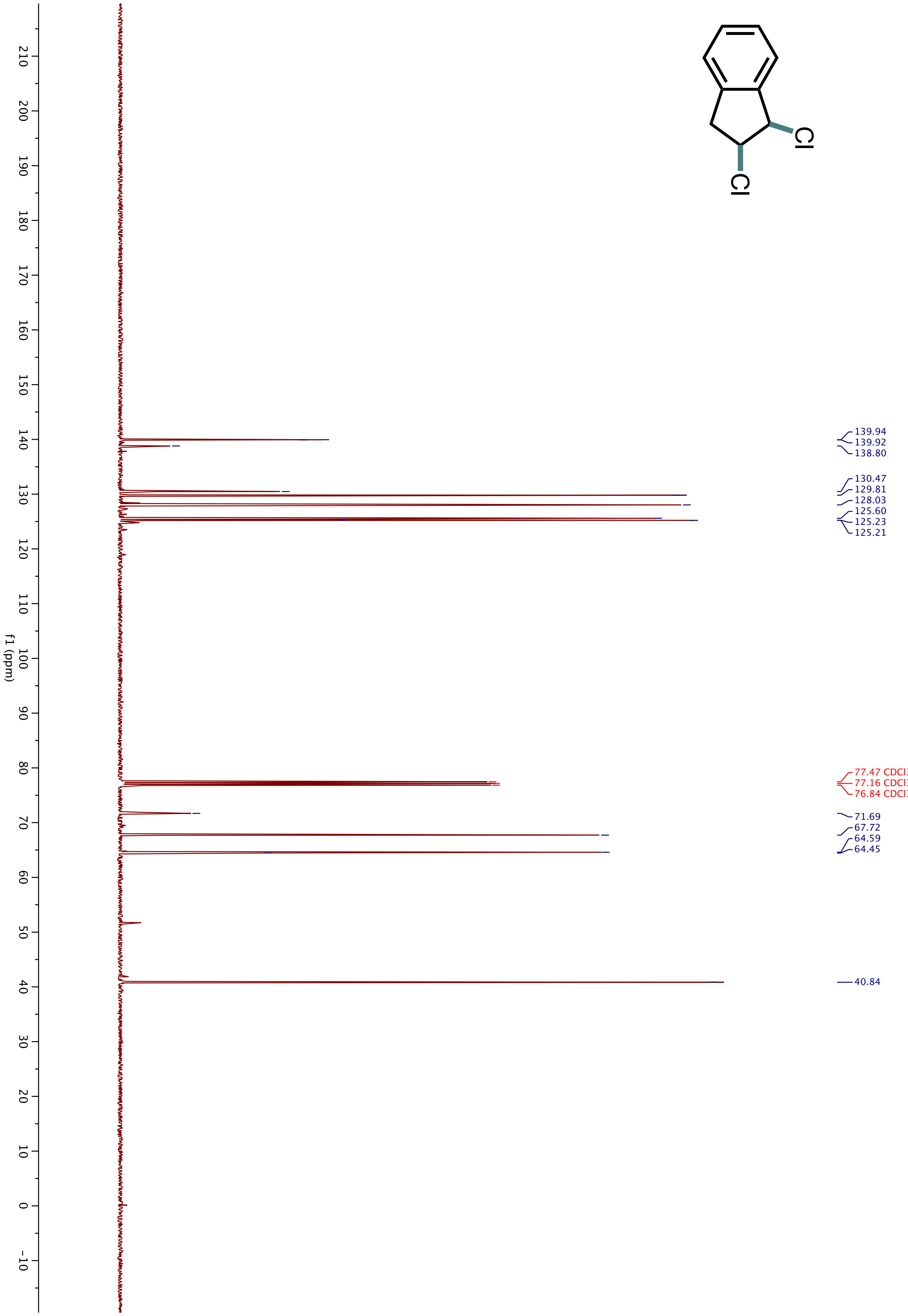

## VIII. References


(1) Buglioni, L.; Raymenants, F.; Slattery, A.; Zondag, S. D. A.; Noël, T. Technological Innovations in Photochemistry for Organic Synthesis: Flow Chemistry, High-Throughput Experimentation, Scale-up, and Photoelectrochemistry. *Chem. Rev.* **2022**, *122*, 2752–2906.

(2) Candish, L.; Collins, K. D.; Cook, G. C.; Douglas, J. J.; Gómez-Suárez, A.; Jolit, A.; Keess, S. Photocatalysis in the Life Science Industry. *Chem. Rev.* **2022**, *122*, 2907–2980.

(3) PhotoRedOx Box. *HepatoChem*. https://hepatochem.com/photoreactors-leds-accessories/photoredox-box/. (accessed March 2026).

(4) Illumin8. *Asynt.* https://www.asynt.com/product/illumin8-parallel-photoreactor (accessed March 2026).

(5) Integrity 10 photoreactor. *Asynt.* https://www.asynt.com/product/integrity-10-reaction-station/ (accessed June 2026).

(6) Solstice. *Asynt.* https://www.asynt.com/product/solstice-parallel-12-position-led-photoreactor/ (accessed June 2026).

(7) Lumidox LED arrays. *Analytical Sales and Services*. https://www.analytical-sales.com/product-category/photoredox-parallel-synthesis/ (accessed March 2026).

(8) PX9 Parallel Screening Photoreactor. *PESCHL Photoreactors* https://photoreactors.com/scale-up/photoreactors-for-screening/ (accessed May 2026).

(9) Bonfield, H. E.; Mercer, K.; Diaz-Rodriguez, A.; Cook, G. C.; McKay, B. S. J.; Slade, P.; Taylor, G. M.; Ooi, W. X.; Williams, J. D.; Roberts, J. P. M.; Murphy, J. A.; Schmermund, L.; Kroutil, W.; Mielke, T.; Cartwright, J.; Grogan, G.; Edwards, L. J. The Right Light: De Novo Design of a Robust Modular Photochemical Reactor for Optimum Batch and Flow Chemistry. *ChemPhotoChem* **2020**, *4*, 45–51.

(10) PhotoCube Plus. *ThalesNano*. https://thalesnano.com/products-and-services/photocube-plus/ (accessed May 2026).

(11) Sun, Z.; Tang, B.; Liu, K. K.-C.; Zhu, H. Y. Direct Photochemical Cross-Coupling between Aliphatic Acids and $BF_3K$ Salts. *Chem. Commun.* **2020**, *56*, 1294–1297.

(12) Schmalzbauer, M.; Svejstrup, T. D.; Fricke, F.; Brandt, P.; Johansson, M. J.; Bergonzini, G.; König, B. Redox-Neutral Photocatalytic C–H Carboxylation of Arenes and Styrenes with $CO_2$. *Chem* **2020**, *6*, 2658–2672.

(13) Lefebvre, Q.; Salomé, C.; Fessard, T. C. Accelerating Fragment-Based Library Generation by Coupling High-Performance Photoreactors with Benchtop Analysis. *Beilstein J. Org. Chem.* **2020**, *16*, 982–988.

(14) Schiel, F.; Peinsipp, C.; Kornigg, S.; Böse, D. A 3D-Printed Open Access Photoreactor Designed for Versatile Applications in Photoredox- and Photoelectrochemical Synthesis. *ChemPhotoChem* **2021**, *5*, 431–437.

(15) Bissonnette, N. B.; Ryu, K. A.; Reyes-Robles, T.; Wilhelm, S.; Tomlinson, J. H.; Crotty, K. A.; Hett, E. C.; Roberts, L. R.; Hazuda, D. J.; Jared Willis, M.; et al. Design of a Multiuse Photoreactor to Enable Visible-Light Photocatalytic Chemical Transformations and Labeling in Live Cells. *ChemBioChem* **2020**, *21*, 3555–3562.

(16) Liu, Y.; Zhou, Y.; Li, D.; Chen, H.; Zhao, J.; Qu, J. Iron-Catalyzed Boration of Cinnamyl Carbonates: A Highly Stereoselective Approach to Cyclopropylboronates. *Org. Chem. Front.* **2019**, *6*, 983–988.

(17) Walker, J. D.; Poli, R. $FeCl_3$-Phosphine Adducts with Trigonal-Bipyramidal Geometry. Influence of the Phosphine on the Spin State. *Inorg. Chem.* **1989**, *28*, 1793–1801.

(18) Crocker, M. S.; Lin, J.-Y.; Nsouli, R.; McLaughlin, N. D.; Musaev, D. G.; Mehranfar, A.; Lopez, E. R.; Ackerman-Biegasiewicz, L. K. G. Transformative Ligand Effects in Fe-Photocatalyzed Giese-type Additions. *Chem Catal.* **2024**, *4*, 101131.
(19) Lin, J.-Y.; Lopez, E. R.; Mehranfar, A.; Bacsa, J.; Ackerman-Biegasiewicz, L. K. G. Elucidation of Mononuclear Fe Photocatalysts for Visible Light-Induced Homolysis (VLIH). *ChemRxiv*. **2025**, doi:10.26434/chemrxiv-2025-djp0r.
(20) Hu, A.; Guo, J.-J.; Pan, H.; Tang, H.; Gao, Z.; Zuo, Z. δ-Selective Functionalization of Alkanols Enabled by Visible-Light-Induced Ligand-to-Metal Charge Transfer *J. Am. Chem. Soc.* **2018**, *140*, 1612–1616.
(21) Lian, P.; Long, W.; Li, J.; Zheng, Y.; Wan, X. Visible-Light-Induced Vicinal Dichlorination of Alkenes through LMCT Excitation of $CuCl_2$. *Angew. Chem. Int. Ed.* **2020**, *59*, 23603–23608.
